# Bioinformatics and systems biology approaches to identify potential common pathogeneses for sarcopenia and osteoarthritis

**DOI:** 10.3389/fmed.2024.1380210

**Published:** 2024-06-18

**Authors:** Jinghong Yang, Jun Zhong, Yimin Du, Zi Wang, Lujun Jiang, Zhong Li, Yanshi Liu

**Affiliations:** ^1^Department of Orthopedics, The Affiliated Hospital, Southwest Medical University, Lu Zhou, China; ^2^Sichuan Provincial Laboratory of Orthopaedic Engineering, Southwest Medical University, Lu Zhou, China; ^3^Stem Cell Immunity and Regeneration Key Laboratory of Luzhou, Southwest Medical University, Lu Zhou, China

**Keywords:** sarcopenia, osteoarthritis, hub genes, bioinformatics, pathogenesis, disease markers

## Abstract

Sarcopenia, a geriatric syndrome characterized by progressive loss of muscle mass and strength, and osteoarthritis, a common degenerative joint disease, are both prevalent in elderly individuals. However, the relationship and molecular mechanisms underlying these two diseases have not been fully elucidated. In this study, we screened microarray data from the Gene Expression Omnibus to identify associations between sarcopenia and osteoarthritis. We employed multiple statistical methods and bioinformatics tools to analyze the shared DEGs (differentially expressed genes). Additionally, we identified 8 hub genes through functional enrichment analysis, protein–protein interaction analysis, transcription factor-gene interaction network analysis, and TF-miRNA coregulatory network analysis. We also discovered potential shared pathways between the two diseases, such as transcriptional misregulation in cancer, the FOXO signalling pathway, and endometrial cancer. Furthermore, based on common DEGs, we found that strophanthidin may be an optimal drug for treating sarcopenia and osteoarthritis, as indicated by the Drug Signatures database. Immune infiltration analysis was also performed on the sarcopenia and osteoarthritis datasets. Finally, receiver operating characteristic (ROC) curves were plotted to verify the reliability of our results. Our findings provide a theoretical foundation for future research on the potential common pathogenesis and molecular mechanisms of sarcopenia and osteoarthritis.

## 1 Introduction

Sarcopenia is a systemic condition characterized by progressive loss of muscle mass and strength in elderly individuals and is closely associated with falls, fractures, and decreased bone density (1). This condition is distinguished by an age-associated, gradual decline in skeletal muscle mass and strength, leading to the manifestation of muscle weakness, limited mobility, and heightened vulnerability to injuries (2). Despite being a relatively recent syndrome initially described in the 1980s (3), sarcopenia has emerged as a prevalent condition, with an estimated prevalence varying between 12.9 and 40.4%, depending on the diagnostic criteria employed (4, 5). Sarcopenia is becoming more common as the older population ages. The number of sarcopenic individuals is predicted to increase to 500 million by the year 2050, from an estimated 50 million individuals today (6). Petermann-Rocha et al. (7) conducted a comprehensive review encompassing 151 studies and concluded that the global prevalence of sarcopenia among individuals aged 60 years and older varied between 10.00 and 27.00%, with the prevalence of severe sarcopenia ranging from 2.00 to 9.00%. It has been reported that the direct cost of medical spending due to sarcopenia was approximately $18.5 billion (i.e., 1.5% of the total health care spending) in 2000 in the United States (8) and that the economic burden of this progressive and generalized skeletal muscular disorder has grown substantially since then (9). Hence, sarcopenia is escalating as a significant burden on public health.

Osteoarthritis (OA), the most prevalent degenerative joint disorder, currently stands as a primary contributor to physical disability (10, 11). As the main cause of disability, OA has gradually increased healthcare and social costs in older adults (12). Therefore, it is important to have a comprehensive understanding of the pathogenesis and potential risk factors for OA (13). Multiple risk factors have been demonstrated to be associated with the pathogenesis of OA (14), among which muscle weakness is considered one of the major risk factors (12, 15). Some scholars believe that functional exercises and strength training can be effective interventions for patients with OA and can reverse the progression of OA to a certain extent. Previous studies have consistently demonstrated a bidirectional association between sarcopenia and OA, indicating that sarcopenia may contribute to the progression of OA, and conversely, OA may exacerbate sarcopenia. On the one hand, muscle weakness weakens the protection of the knee, increasing susceptibility to cartilage damage and increasing the prevalence of OA (16). On the contrary, the pain and stiffness associated with OA joints promote physical inactivity among patients, ultimately leading to adipose tissue accumulation and muscle weakness. This, in turn, can exacerbate the progression of OA in a vicious cycle, as muscle loss further contributes to the disease’s severity (17, 18), and the coexistence of sarcopenia and OA is becoming increasingly prevalent as global ageing increases. Therefore, it is necessary to explore the relevance of these two musculoskeletal system disorders.

Given the existence of numerous bidirectional associations between sarcopenia and OA, it is evident that one condition can significantly increase the likelihood of developing the other (17, 18), our study stands as the initial investigation to identify pivotal genes implicated in the pathogenesis of OA complicated by sarcopenia. These findings could lead to new ideas for the diagnosis and treatment of sarcopenia and OA. Through integrated bioinformatics and systems biology analyses, we identified 8 hub genes implicated in sarcopenia and osteoarthritis (OA) and uncovered 32 shared differentially expressed genes (DEGs). Leveraging these DEGs and hub genes, we constructed various functional annotations, including protein–protein interaction (PPI) networks, transcription factor (TF)-gene regulatory networks, microRNA (miRNA)-gene regulatory networks, gene-disease association networks, and immune infiltration networks. Furthermore, we utilized the Drug Signatures database to identify the top 10 candidate drugs, advancing the potential therapeutic options for sarcopenia and OA. These hub genes offer novel insights into the biological mechanisms underlying OA and sarcopenia, potentially paving the way for the discovery of innovative therapeutic targets for patients suffering from these conditions.

## 2 Results

### 2.1 Analysis of differential gene expression in sarcopenia and osteoarthritis patients

**Figure 1:**
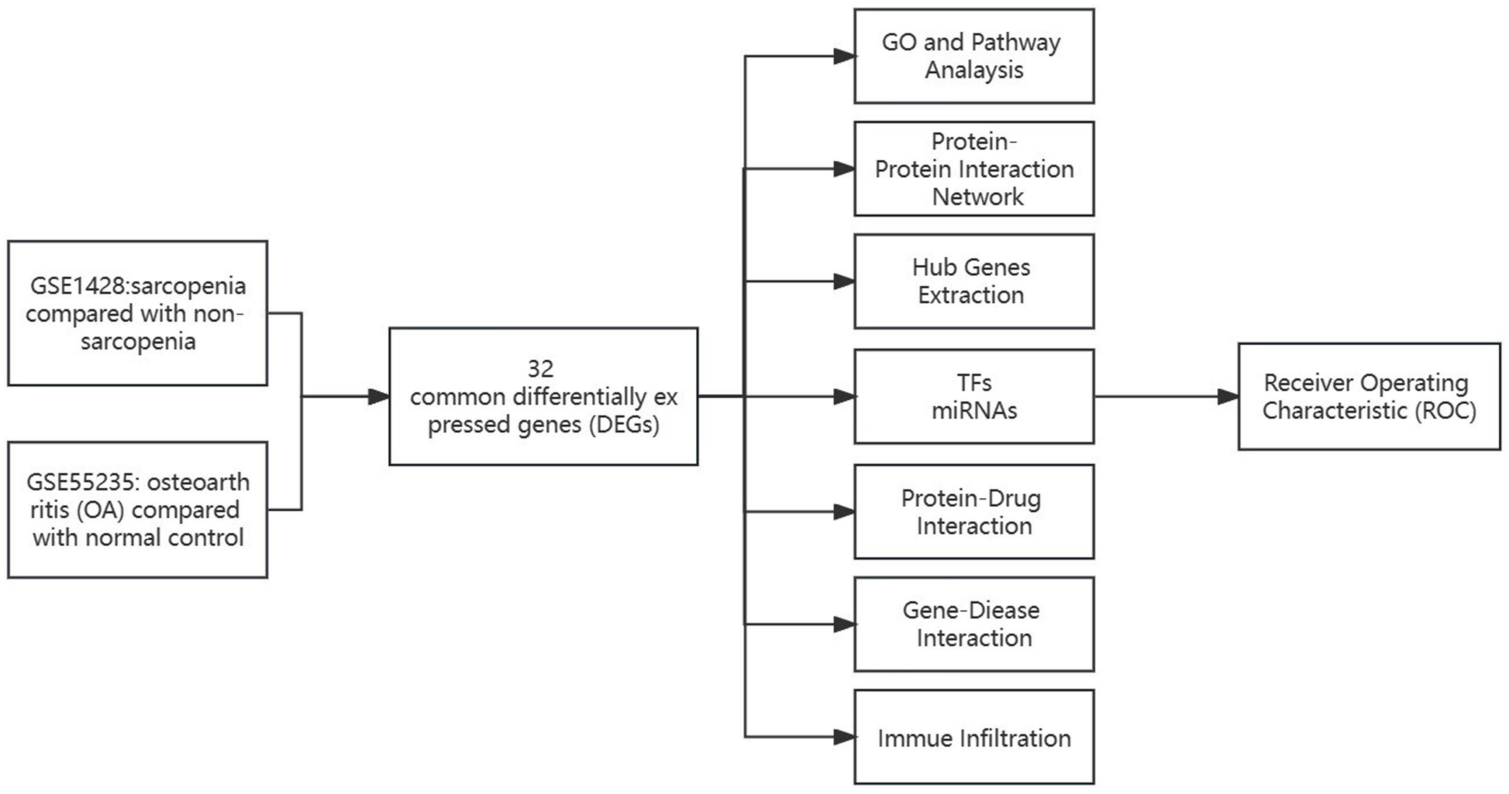
Schematic illustration of the overall workflow of this study.

**Figure 2:**
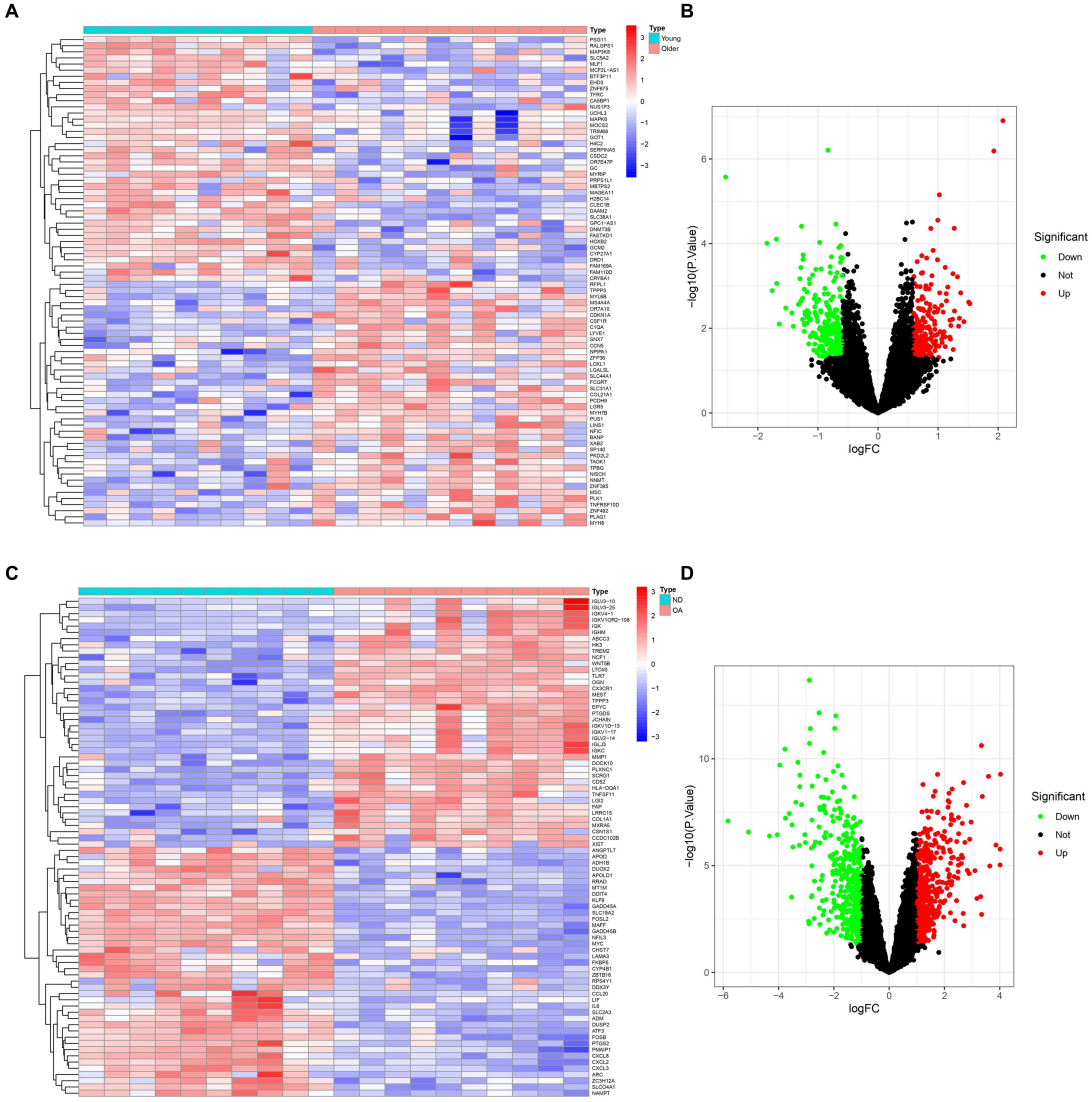
Expression characteristics of DEGs in sarcopenia patients and OA patients. **(A)** Sarcopenia-Heatmap and **(B)** Sarcopenia-Volcano plot **(C)** OA-Heatmap **(D)** OA-Volcano plot **(A,B)** present the DEGs identified between patients with sarcopenia and healthy controls (|logFC| >0.585 was defined as the screening criterion to obtain DEGs in sarcopenia). **(C,D)** The DEGs identified between OA patients and healthy controls (|logFC| >1) were defined as the screening criterion for obtaining DEGs for OA. Blue indicates low expression values, and red indicates high expression values.

**Figure 3:**
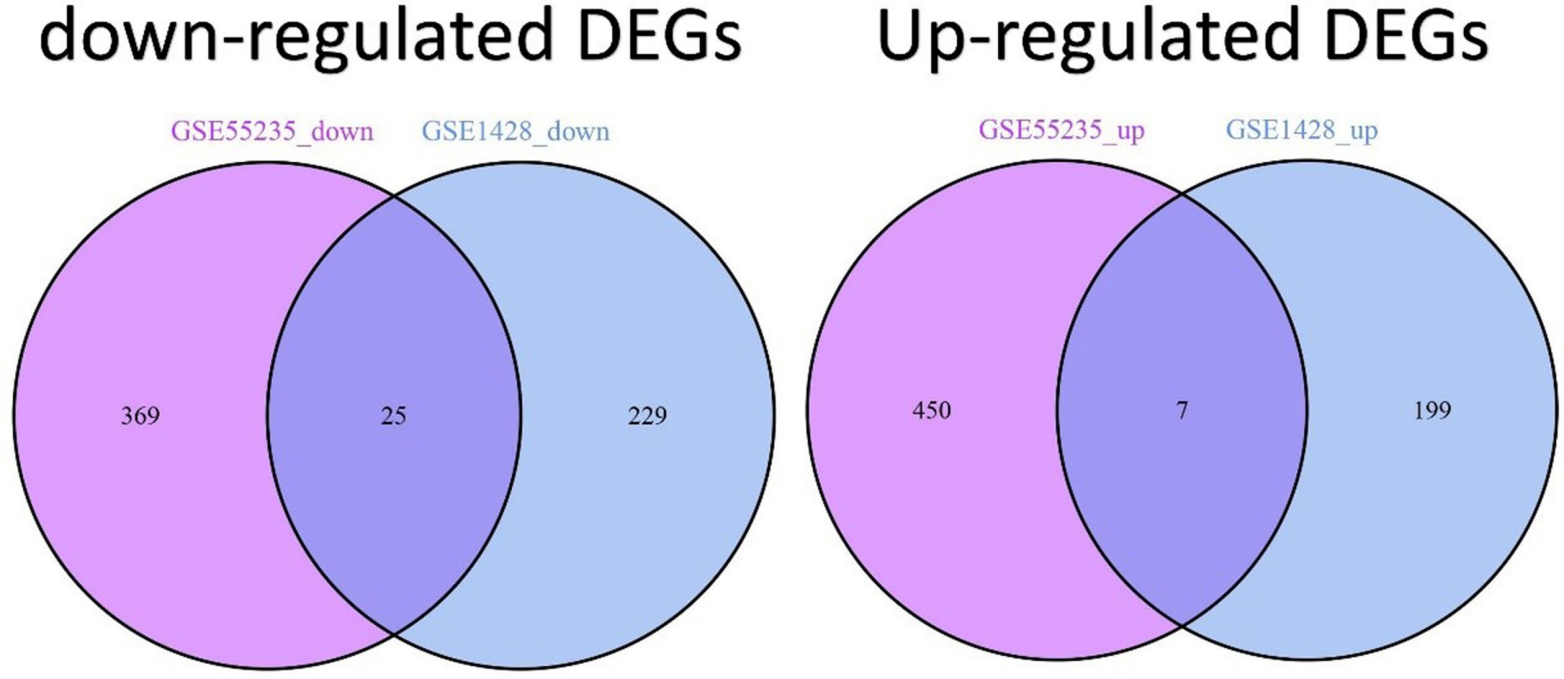
Identification of DEGs shared between sarcopenia and OA patients. Venn diagrams show two datasets containing 7 shared upregulated DEGs and 25 shared downregulated DEGs.

The comprehensive flowchart of this study is depicted in Figure 1. In the GSE1428 dataset, a total of 460 differentially expressed genes (DEGs) were discovered, whereas 851 DEGs were identified in the GSE55235 dataset. Utilizing the predefined criteria for *p*-value and fold change in differentially expressed genes (DEGs), we identified 199 upregulated and 229 downregulated DEGs in sarcopenia patients, as well as 450 upregulated and 369 downregulated DEGs in osteoarthritis (OA) patients. Figure 2 graphically illustrate the DEG distributions, comparing patients with sarcopenia to those without, and patients with OA to healthy controls, respectively, through heatmaps and volcano plots. DEG overlap was calculated to identify common DEGs for both diseases (Figure 3). Seven common upregulated genes and 25 common downregulated genes were identified for further analysis (Figure 3).

### 2.2 Gene ontology and KEGG pathway analysis results

**Figure 4:**
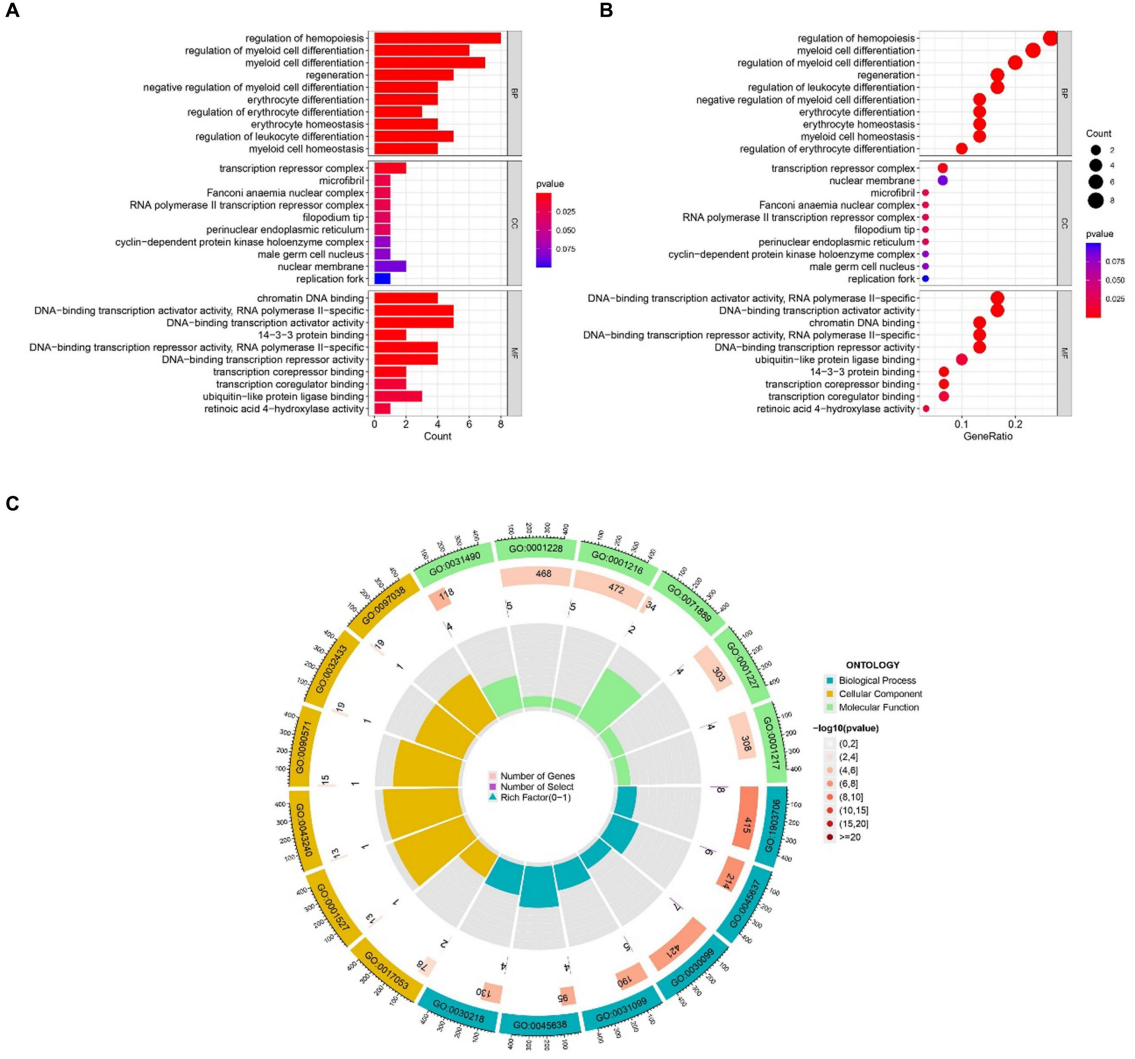
GO functional enrichment analysis of genes shared between sarcopenia patients and OA patients. **(A)** Bar plot. **(B)** Bubble plot. **(C)** Circle chart. The results are shown as-log10 (*p* value).

**Figure 5:**
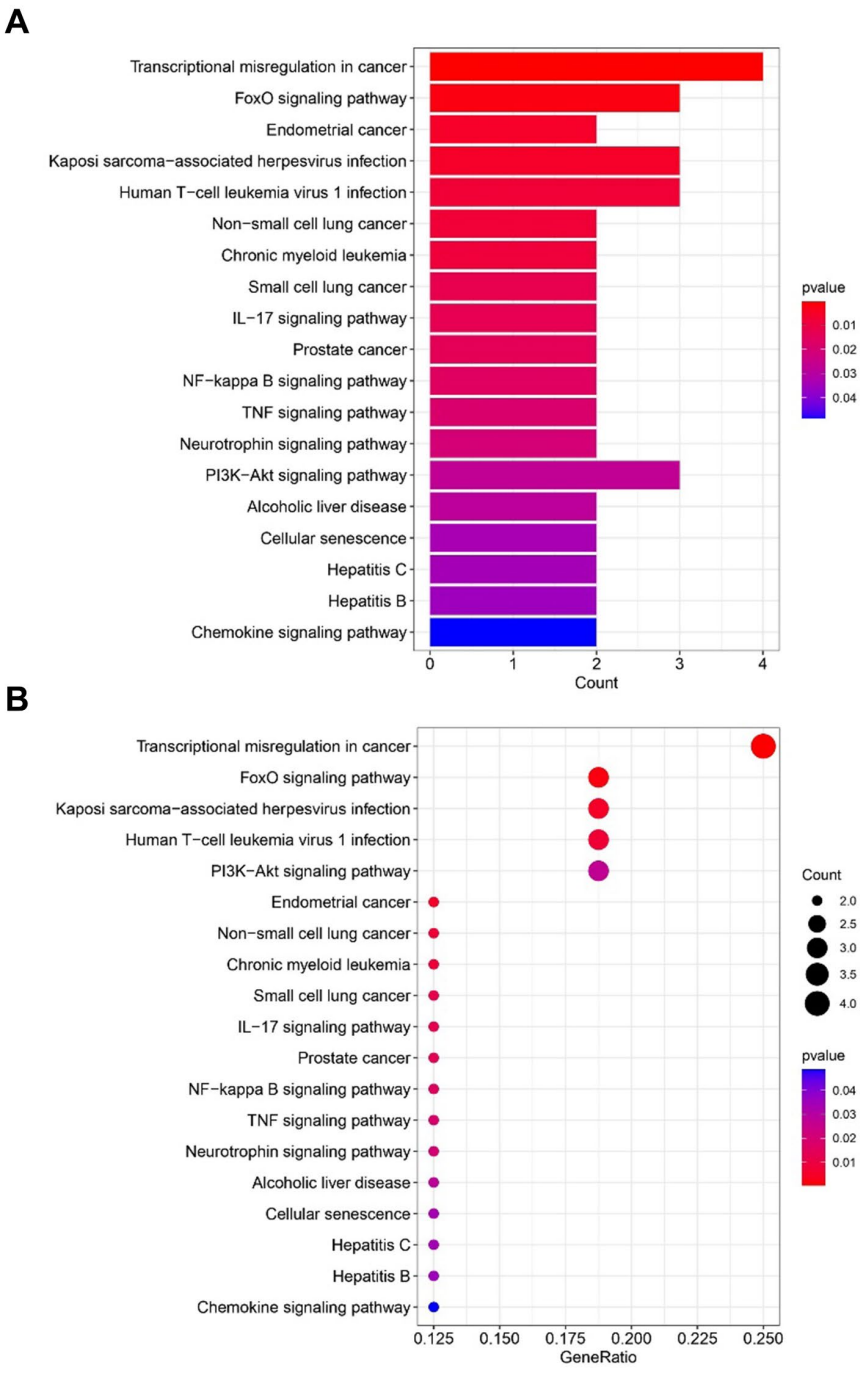
Functional enrichment analysis of KEGG pathways revealed genes shared between sarcopenia patients and OAs. **(A)** Barplot. **(B)** Bubble plot. The results are shown as the-log10 (*p*-value).

The most prominent terms within the gene ontology (GO) categories of biological process, molecular function, and cellular component were “positive regulation of hemopoiesis,” “chromatin DNA binding,” and “transcription repressor complex,” respectively (Figure 4). The pathways enriched in the common DEGs between sarcopenia patients and OA patients were identified via KEGG enrichment analysis and are shown in Figure 5.

### 2.3 Analyzing and visualizing protein–protein interaction network

**Figure 6:**
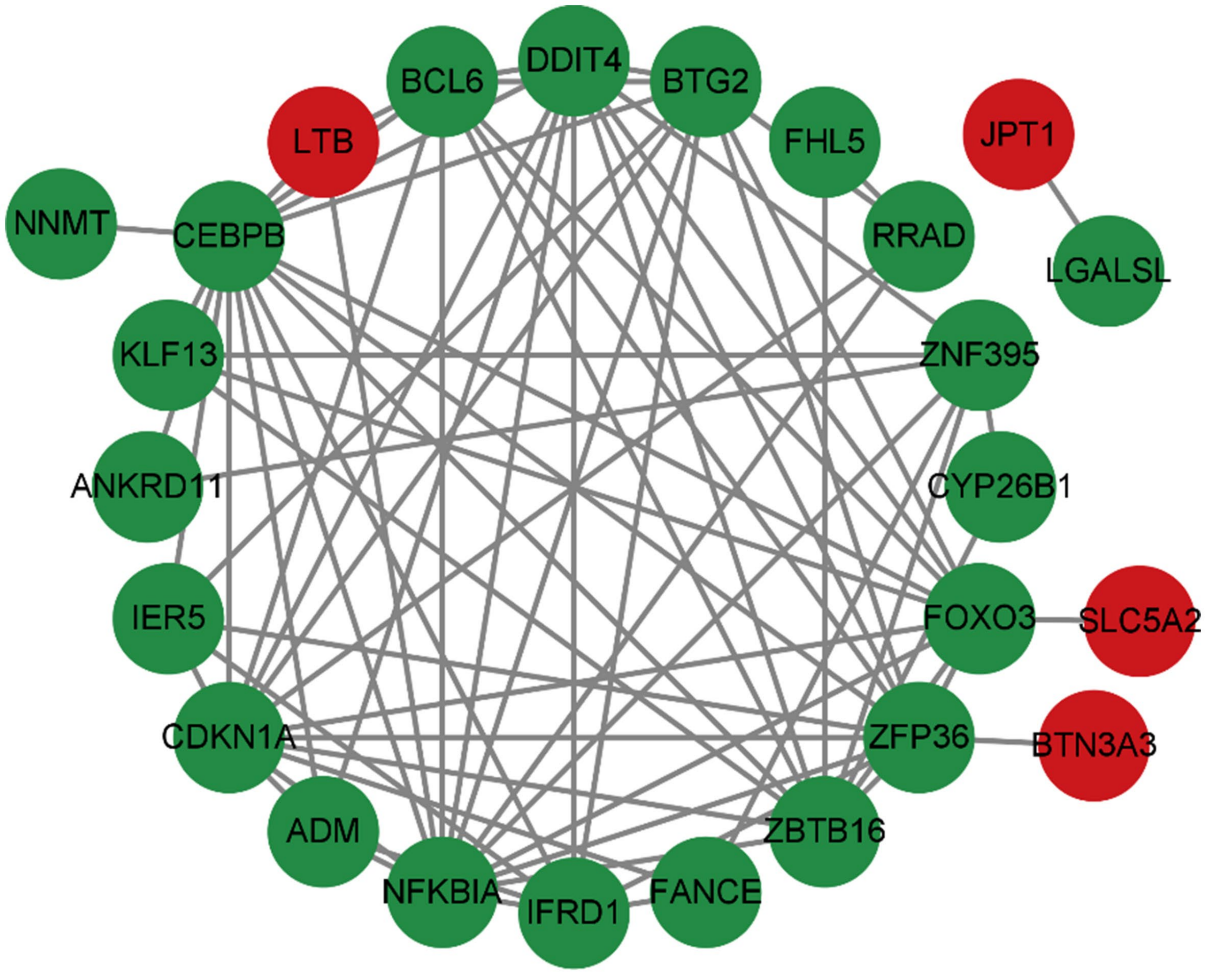
The PPI network of DEGs was shared between OA and sarcopenia patients.

To predict common differentially expressed gene (DEG) interactions and adhesion pathways, we meticulously analyzed and visualized the protein–protein interaction (PPI) network in STRING. The PPI network, composed of 32 nodes and 76 edges, exhibited a *p* < 2.05e-08, as depicted in Figure 6.

### 2.4 Classification and functional analysis of the hub genes

**Figure 7:**
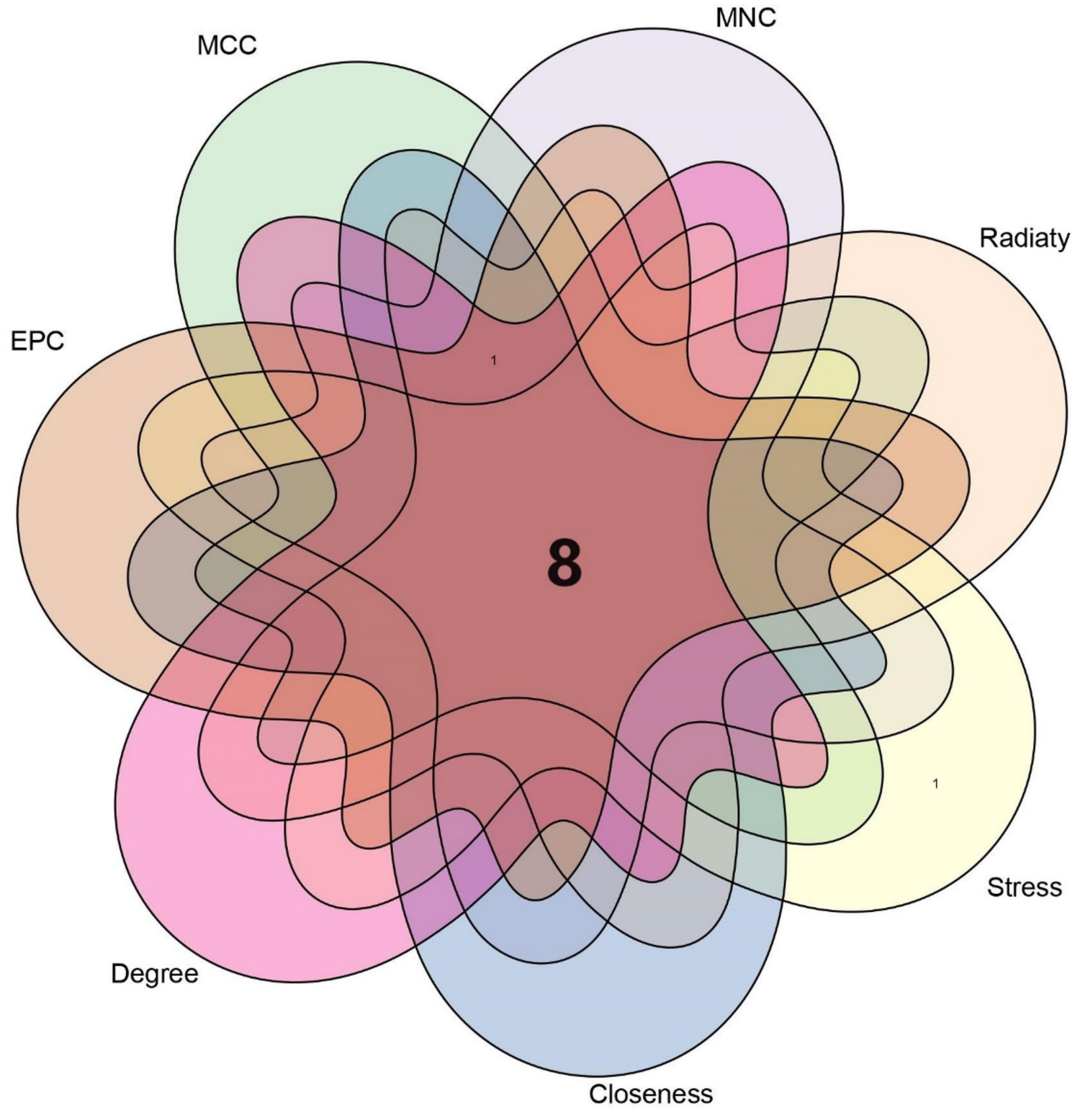
Venn diagram showing 7 algorithms screening for 8 overlapping hub genes.

The top 37 hub genes were screened by applying the 7 algorithms of the Cytoscape plugin cytoHubba. Eight common hub genes, BTG2, CDKN1A, CEBPB, DDIT4, FOXO3, NFKBIA, ZBTB16, and ZFP36, were ultimately identified by Venn diagram crossover (Figure 7).

**Figure 8:**
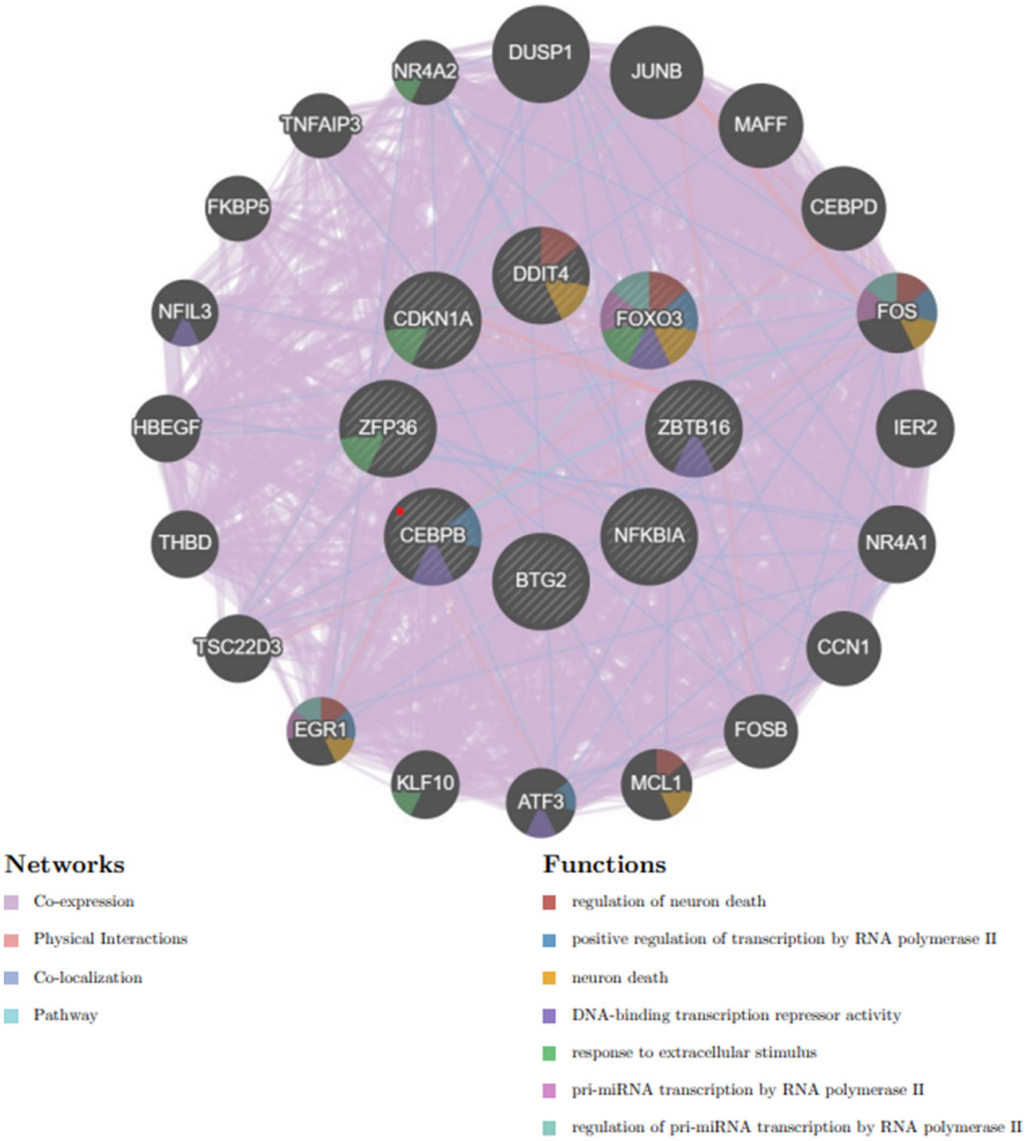
Hub genes and their coexpressed genes were analyzed via GeneMANIA.

A complex network of gene interactions was constructed to decipher the biological functions of these hub genes via the GeneMANIA database (Figure 8), with coexpression of 94.8%, physical interactions of 3.24%, pathways of 0.88%, and colocalization of 1.08%. The results also showed that these genes were related to the regulation of neuron death, positive regulation of transcription by RNA polymerase II, neuron death, DNA-binding transcription repressor activity, response to extracellular stimulus, pri-miRNA transcription by RNA polymerase II, and regulation of pri-miRNA transcription by RNA polymerase II. These hub genes have the potential to serve as biomarkers and could pave the way for novel therapeutic strategies in the investigation of these diseases.

### 2.5 Determination of regulatory signatures

**Figure 9:**
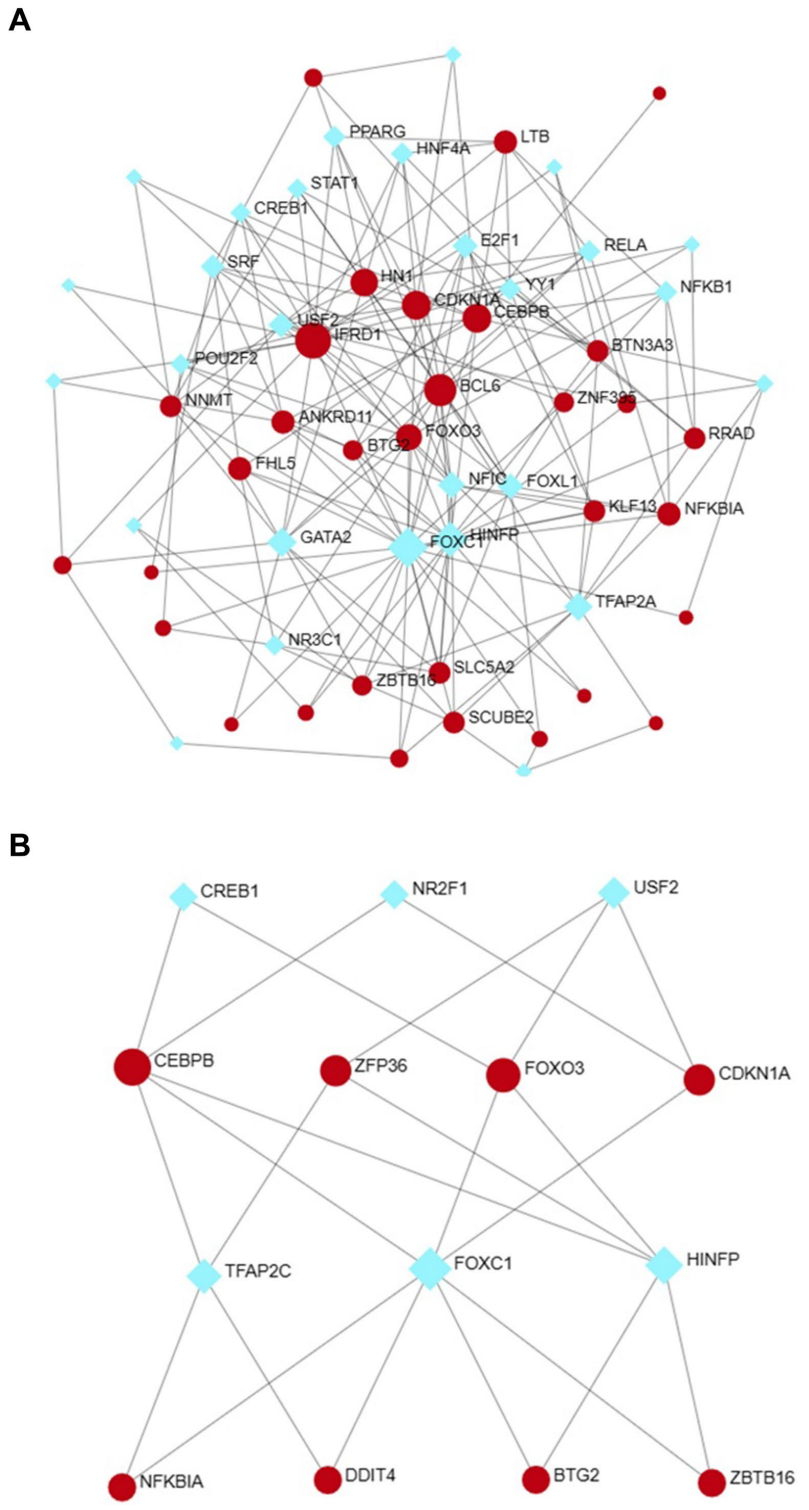
The DEG-TF **(A)** and hub gene-TF **(B)** regulatory interaction networks. Herein, the square nodes are TFs, and the gene symbols that interact with TFs are represented as circle nodes. DEGs, differentially expressed genes; TF, transcription factor.

**Figure 10:**
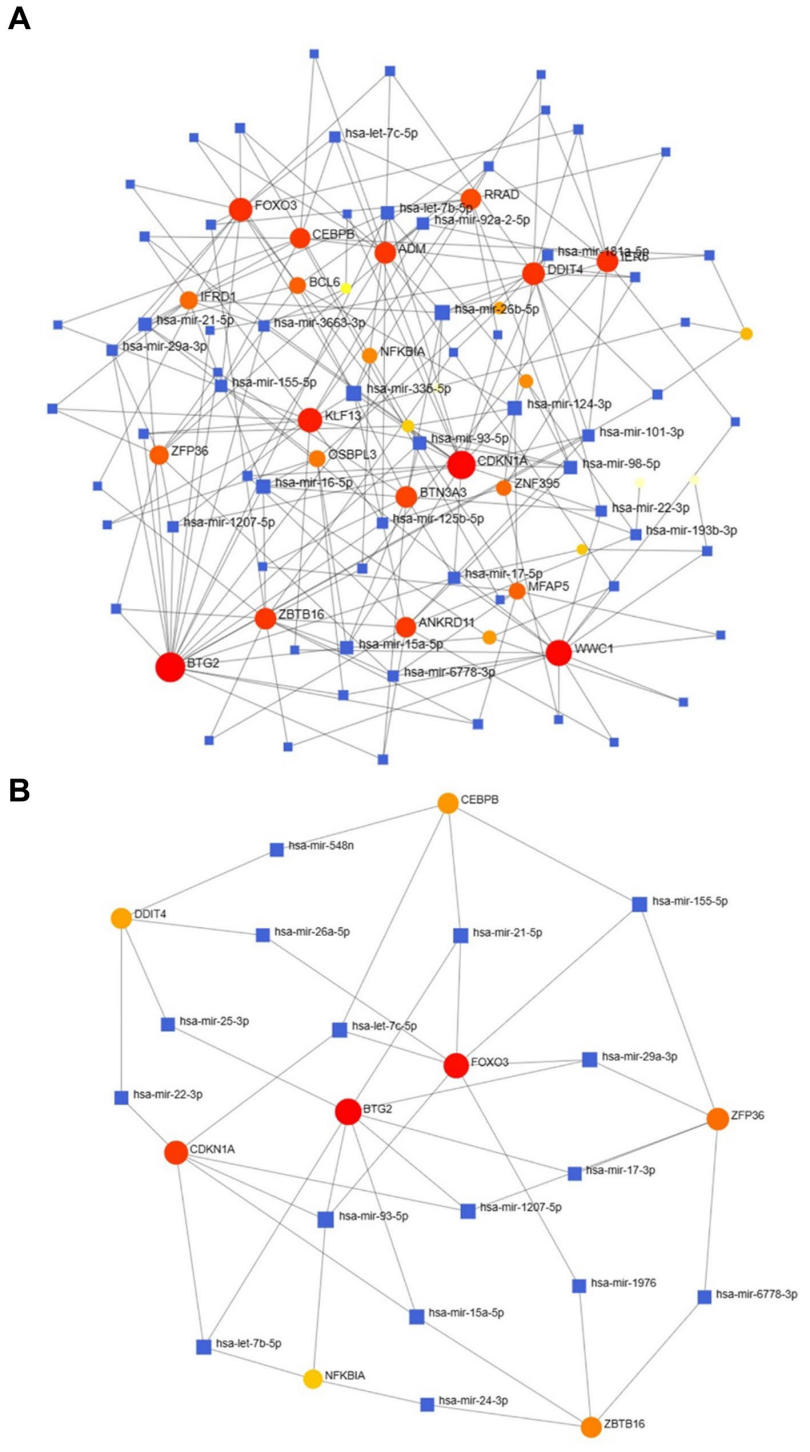
The DEG–miRNA **(A)** and hub gene–miRNA **(B)** regulatory interaction networks. Herein, the square nodes indicate miRNAs, and the gene symbols indicate interactions with miRNAs in a circular shape. DEGs, differentially expressed genes; miRNAs, microRNAs.

Utilizing NetworkAnalyst, the interactions between transcription factors (TFs) and microRNAs (miRNAs) with differentially expressed genes (DEGs) and eight hub genes were predicted and visualized (Figures 9, 10). Through the analysis of the TF-gene and miRNA-gene interaction networks, we discovered that the TF-gene interaction network encompassing the eight hub genes comprised 96 nodes, 223 edges, and 30 genes. Additionally, the miRNA-gene interaction network encompassed 1,157 nodes, 1,184 edges, and 32 genes.

### 2.6 Disease associations were identified

**Figure 11:**
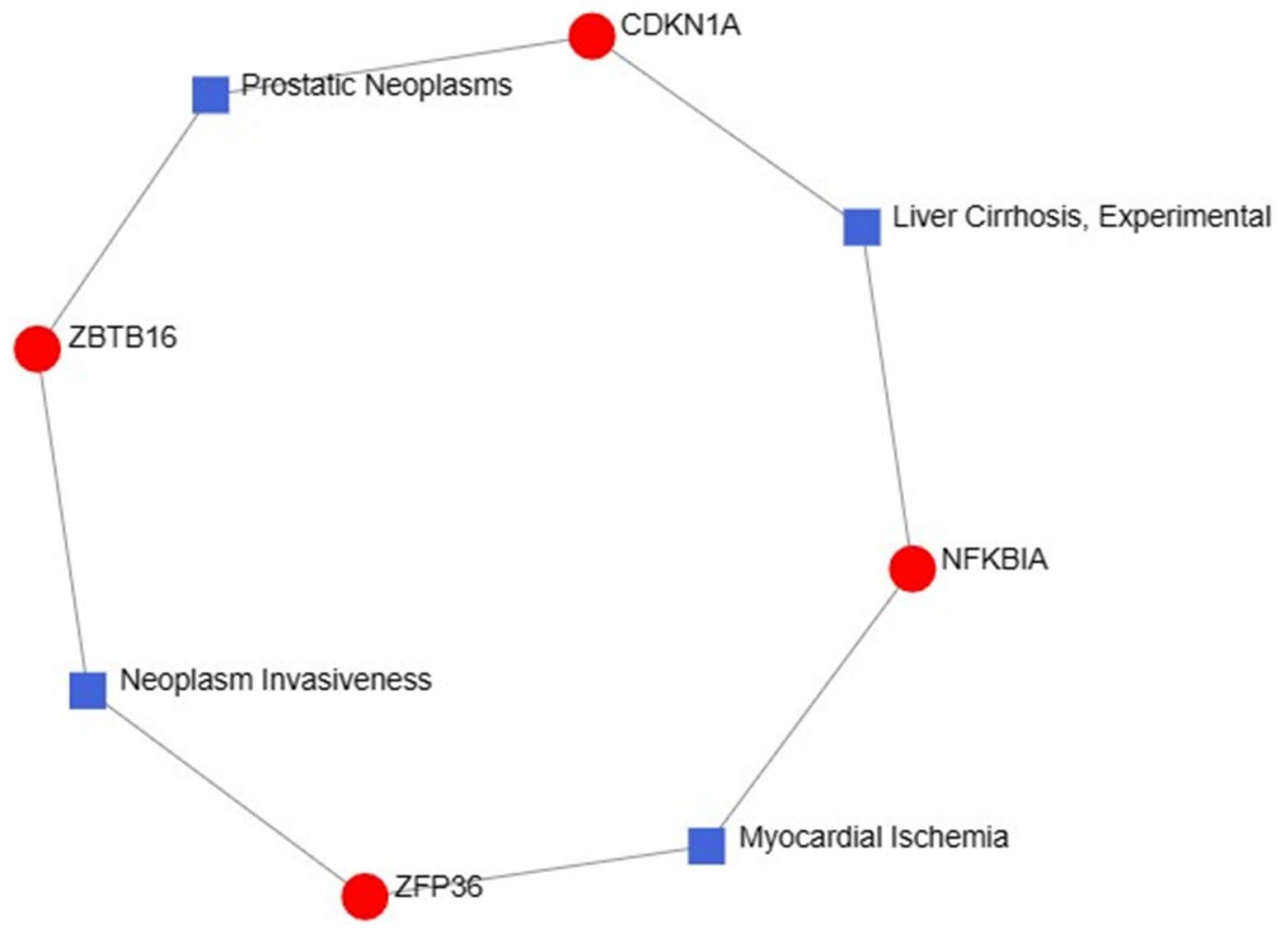
Gene–disease association networks represent diseases associated with hub genes. Diseases are represented by square nodes, and genes are represented by round nodes.

The exploration of therapeutic design strategies for disease treatment offers an avenue to unravel the intricate relationship between genes and disease. Through NetworkAnalyst analysis of gene-disease associations, we found that “prostatic neoplasms,” “experimental liver cirrhosis,” “myocardial ischemia” and “neoplasm invasiveness” were the most related hub genes. Figure 11 depicts the intricate relationships between genes and disease.

### 2.7 Top 10 candidate drugs were identified

**Figure 12:**
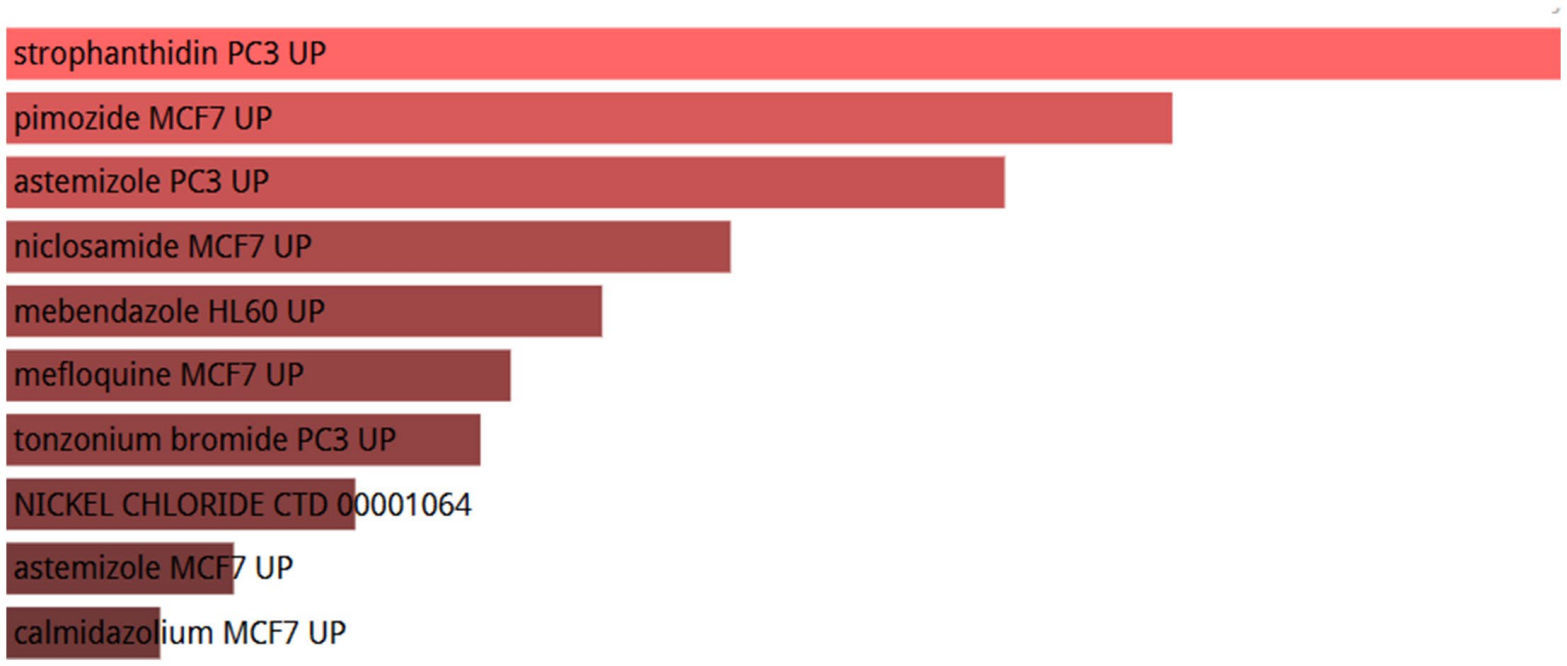
List of the top 10 drugs recommended for patients with OA and sarcopenia.

To investigate the regulatory effects of small molecule drugs on hub gene expression, we retrieved data from the Drug Signatures database (DSigDB) within the enrichment platform. The potential small molecules were prioritized based on their *p* values, reflecting the degree of association between these molecules and the genes of interest. The top 10 potential small molecule drugs for the hub genes are shown in Figure 12.

### 2.8 Assessing diagnostic efficacy of hub genes in sarcopenia and OA

**Figure 13:**
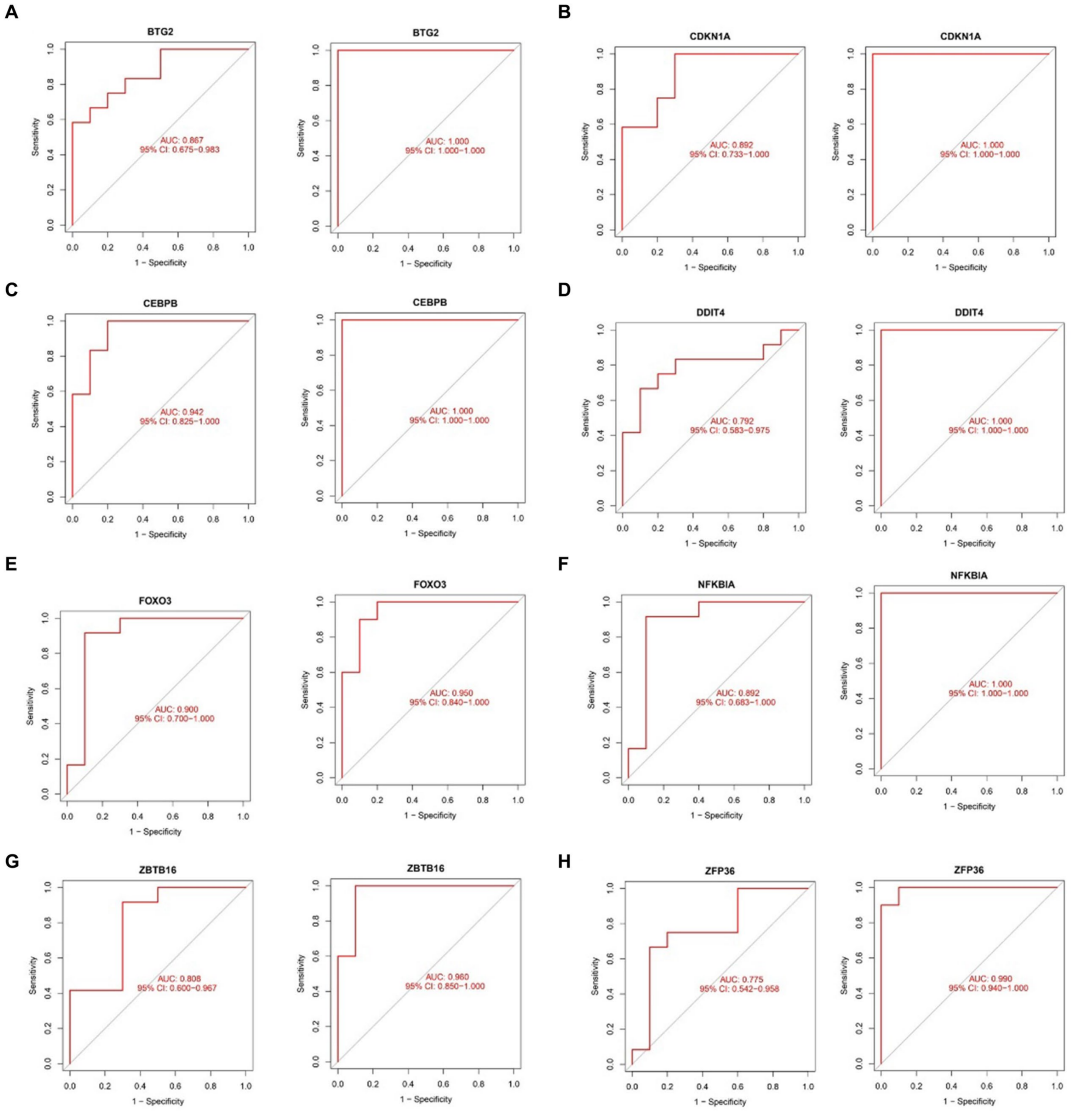
Validation of the diagnostic shared hub genes in the sarcopenia patient (GSE1428) dataset and the OA (GSE5523) dataset. **(A)** BTG2, **(B)** CDKNIA, **(C)** CEBPB, **(D)** DDIT4, **(E)** FOXO3, **(F)** NFKBIA, **(G)** ZBTB16, **(H)** ZFP36.

The ROC curve was plotted to evaluate the diagnostic efficacy of the 8 key genes (Figure 13). In the sarcopenia dataset, BTG2 (AUC = 0.867), CDKN1A (AUC = 0.892), CEBPB (AUC = 0.942), DDIT4 (AUC = 0.792), FOXO3 (AUC = 0.900), NFKBIA (AUC = 0.892), ZBTB16 (AUC = 0.808) and ZFP36 (AUC = 0.775) demonstrated better diagnostic efficacy in distinguishing sarcopenia patients from healthy controls. Coincidentally, all 8 hub genes in the OA dataset were strongly related to the differential diagnosis of OA. Specifically, in the sarcopenia dataset, the CEBPB showed the best diagnostic efficiency for differentiating sarcopenia, while BTG2, CDKN1A, CEBPB, DDIT4, and NFKBIA showed the best-differentiating capability in the OA dataset.

### 2.9 Analyzing immune infiltration and hub gene interactions in sarcopenia and OA

**Figure 14:**
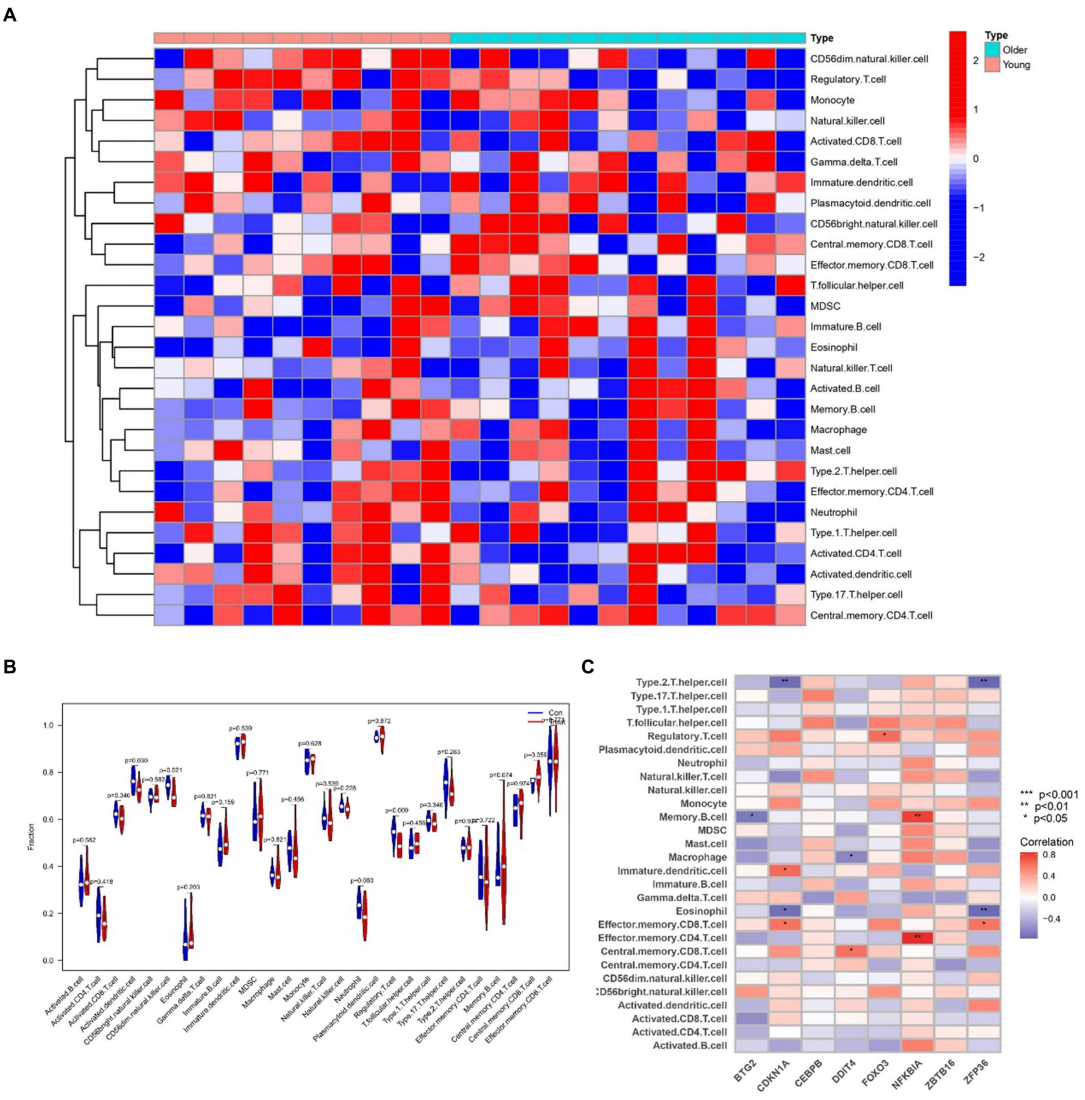
Infiltration analysis and correlation analysis of immune cells in the sarcopenia group and healthy controls. **(A)** Heatmap of immune cell subpopulations in the sarcopenia cohort. **(B)** Violin plot of immune cell subpopulations in the sarcopenia dataset. **(C)** Correlations of immune cell subpopulations with shared key genes. *p* < 0.05 indicated a significant difference.

**Figure 15:**
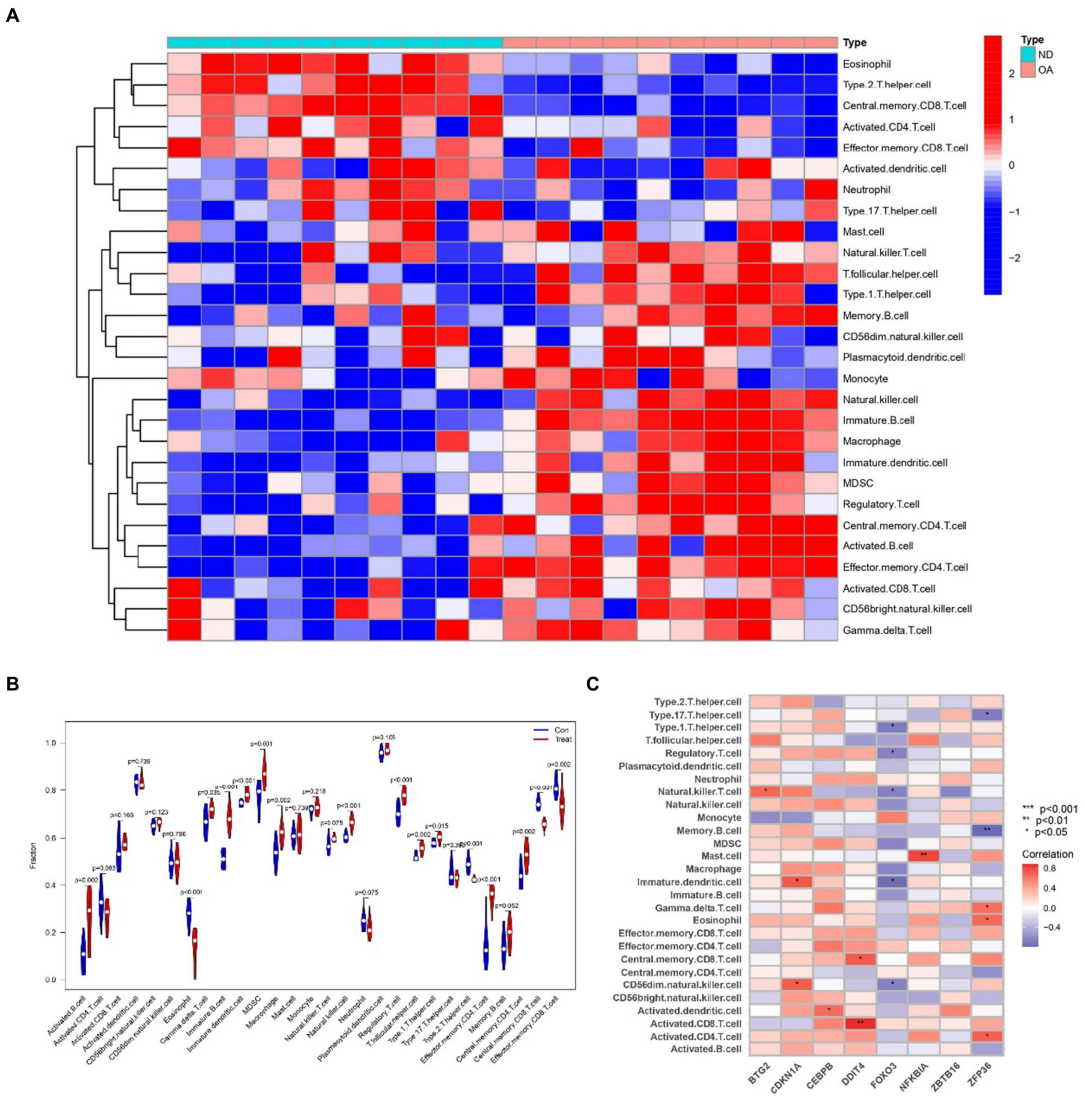
Infiltration analysis and correlation analysis of immune cells in the OA group and healthy controls. **(A)** Heatmap of immune cell subpopulations in the OA dataset. **(B)** Violin plot of immune cell subpopulations in the OA dataset. **(C)** Correlations of immune cell subpopulations with shared key genes. *p* < 0.05 indicated a significant difference.

To delve into the intricate connections between the immune system and the concurrent occurrence of sarcopenia and OA, we conducted immune infiltration analysis on the respective datasets. Furthermore, we examined the interplay between hub genes and immune cells in sarcopenia patients, and these relationships are graphically represented in Figure 14C. These relationships were detected with Eosinophil, Memory B-cell, and Type 2 cells. T helper cells were the cells most strongly associated with the hub genes. Figure 15C illustrates the intricate relationships between hub genes and immune cells in OA, highlighting activated CD8 T cells, immature dendritic cells, natural killer T cells, and mast cells as the most prominent cell types associated with these genes. The interactions between sarcopenia and immune cells, as well as those between OA and immune cells, are comprehensively visualized in Figures 14A,B, 15A,B, presented as a heatmap and violin plot, respectively.

## 3 Discussion

Sarcopenia, a relatively recently recognized condition, exhibits a significant incidence and prevalence among the elderly population, posing a grave threat to the health and well-being of older individuals globally. Several scholars have suggested that sarcopenia may coexist with OA (19). Despite the high occurrence of sarcopenia and osteoarthritis (OA) in the elderly, their pathophysiological mechanisms remain elusive. Ageing, disuse, and inflammation are among the potential contributing factors, yet their significance remains unconfirmed. Notably, only a limited number of studies have explored the genetic interconnections between these two comorbidities. Therefore, to explore the relationship between these two prevalent diseases, bioinformatics and systems biology analyses must be performed to determine the functions and pathways shared between sarcopenia and OA.

In this study, 32 shared DEGs and 8 hub genes (BTG2, CDKN1A, CEBPB, DDIT4, FOXO3, NFKBIA, ZBTB16, and ZFP36) were identified to establish a PPI network, TF-gene interaction network, and DEG-miRNA coregulatory network. In addition, we suggest that potential drugs be used to treat sarcopenia and OA, and the relationships between the immune system and the co-occurrence of sarcopenia and OA should be explored.

The 32 identified common DEGs were selected for exploring GO terms. GO terms were selected according to the *p* values. For biological processes, regulation of hemopoiesis, regulation of myeloid cell differentiation, and myeloid cell differentiation were among the top GO terms. A previous study demonstrated that Runt-related transcription factor-1 (Runx1), renowned for its roles in hemopoiesis and leukaemia, possesses the ability to regulate crucial genes such as collagen type II (Col2a1) and X (Col10a1), SRY-box transcription factor 9 (Sox9), aggrecan (Acan), and matrix metalloproteinase 13 (MMP-13). Notably, the upregulation of Runx1 expression was found to enhance the overall homeostasis of the joint (20). Myeloid cell differentiation is an important part of cell differentiation. Single-cell RNA sequencing (21) provided further insights into the cellular dynamics within osteocyte ablation mice, revealing a shift in hematopoietic lineage towards myeloid lineage differentiation, characterized by an expansion of myeloid progenitors, neutrophils, and monocytes. Conversely, lymphopoiesis was compromised, manifesting as a decrease in B cell populations. These findings reveal the important role of myeloid cell differentiation in OA and sarcopenia. GO terms in terms of molecular function chromatin DNA binding, DNA − binding transcription activator activity, RNA polymerase II − specific, and DNA − binding transcription activator activity were considered to be at the top of the list. The top GO terms associated with the cellular component category were “transcription repressor complex,” “microfibril” and “Fanconi anaemia nuclear complex.”

The determination of the KEGG pathway was performed for the 32 common DEGs to identify pathways related to both sarcopenia and OA. The top 10 KEGG pathways included “Transcriptional misregulation in cancer,” “FOXO signalling pathway,” “Endometrial cancer,” “Kaposi sarcoma−associated herpesvirus infection,” “Human T − cell leukaemia virus 1 infection,” “On−small cell lung cancer,” “Chronic myeloid leukemia,” “Small cell lung cancer,” the IL − 17 signalling pathway” and “Prostate cancer.” Transcriptional misregulation in cancer and treatment with small molecule inhibitors ultimately affect RNA polymerase II (Pol II)-dependent gene transcription, which is dynamically controlled by regulatory networks (22). This can lead to the cessation of the transcription cycle and cellular heterogeneity and can exacerbate cellular and even organismal senescence (22). The FOXO signalling pathway is regulated mainly by PKB/Akt and MAPK and plays an important role in the induction of autophagy. Recent studies have shown that secondary metabolites of quinoa can antagonize sarcopenia by regulating protein levels in muscle cells via the FOXO signalling pathway (23). Another study has revealed the crucial roles of the AMPK-ULK1 and FOXO/PGC-1ɑ signalling pathways in the induction of autophagy mechanisms within skeletal muscle and bone (24). Furthermore, an analysis of WikiPathways data revealed that the most frequently intersecting gene pathways were implicated in transcriptional misregulation in cancer, the FOXO signalling pathway, Kaposi sarcoma-associated herpesvirus infection, and human T-cell leukemia virus 1 infection. Notably, the KEGG pathway analysis underscored the significance of the FOXO signalling pathway and transcriptional misregulation in cancer as the most pivotal factors underlying the concurrent occurrence of sarcopenia and osteoarthritis.

The analysis of the PPI network played a crucial role in pinpointing potential hub genes that are implicated in the common mechanisms underlying both sarcopenia and OA. The 32 commonly identified DEGs were further analyzed through PPI analysis, and the findings showed that BTG2, CDKN1A, CEBPB, DDIT4, FOXO3, NFKBIA, ZBTB16, and ZFP36 were chosen as hub genes due to their high interaction rates and high degree values. BTG2 is known for its role in regulating cell cycle progression and apoptosis. Its expression can influence the progression of osteoarthritis and sarcopenia by modulating chondrocyte apoptosis and regulating muscle differentiation. DDIT4 (DNA Damage Inducible Transcript 4), which is also known as REDD1, is involved in the cellular response to stress and hypoxia. In sarcopenia and OA patients, DDIT4/REDD1 modulates mTOR signalling pathways, contributing to muscle protein synthesis and degradation balance, affecting chondrocyte survival and function under stress conditions, thus playing an important role in muscle atrophy and OA. Inflammation plays a crucial role in the onset and progression of osteoarthritis and sarcopenia. The genes CEBPB and NFKBIA, which are regulated by inflammatory processes, are significantly implicated in both conditions. NFKBIA inhibits the NF-κB signalling pathway, thereby modulating inflammatory responses. Conversely, CEBPB functions as a transcription factor involved in the regulation of adipocyte differentiation and inflammation. Not coincidentally, FOXO3 is also a transcription factor that plays an important role in OA and sarcopenia. A study (25) demonstrated that CircFOXO3 exerts protective effects against osteoarthritis (OA) by targeting its parental gene, FOXO3, and promoting PI3K/AKT-mediated autophagy. Liu et al. (26) reported that the activation of FOXO3 serves as a crucial mechanism underlying denervation-induced muscle atrophy. FOXO3 regulates cell proliferation and differentiation via autophagy, thereby exerting significant effects on the growth, development, and senescence degradation of muscle and bone tissues.

TFs are essential for regulating biomolecules, which are proteins that control the transcription of DNA into RNA by binding to specific DNA sequences (27). In addition, miRNAs are involved in the regulation of protein expression mainly by binding to target sites on mRNA transcripts and inhibiting their translation (28). TF genes and miRNAs are crucial regulatory biomolecules and potential biomarkers that play pivotal roles in determining post-transcriptional transcription ratios and RNA silencing processes, respectively (29). A TF-gene interaction network was constructed based on the commonly identified DEGs. Within this network, IFRD1 exhibited a high degree of interaction with other TF genes, having a network degree of 18. Notably, FOXC1, among the regulators, demonstrated a significant interaction with a degree value of 22. Previous studies have demonstrated that FOXC1 contributes to the pathogenesis of OA by upregulating β-catenin in synovial fibroblasts (30). Another study showed that miR-204-5p inhibits inflammation in synovial fibroblasts in OA by suppressing FOXC1 (31). We subsequently constructed a TF gene interaction network from the hub genes. CEBPB had a high interaction rate with other TF genes, with a degree value of 7 in the network, and FOXC1 still had a significant interaction, with a degree value of 22. Additionally, DEG-miRNA coregulatory network analysis revealed connections between common DEGs and miRNAs via DEG-miRNA coregulatory network visualization. As a result, 96 miRNAs and 30 genes were revealed in this study. Among the most common interacting miRNAs, hsa-mir-26b-5p and hsa-mir-335-5p had high degrees of interaction (8). Zhang et al. (32) demonstrated that CircRNA circ_SEC24A upregulates DNMT3A expression by sequestering miR-26b-5p, thereby exacerbating osteoarthritis progression. Additionally, Lu et al. (33) reported that MicroRNA-26b-5p mitigates murine collagen-induced arthritis by regulating Th17 cell plasticity. Numerous studies have highlighted alterations in miRNA expression patterns in osteoarthritis (OA) patients, with predictions suggesting a significant involvement of miR-26b-5p in the pathogenesis of OA. Tornero Esteban et al. (34) reported that hsa-miR-335-5p was significantly downregulated during osteogenesis in bone marrow mesenchymal stem cells from OA patients, which may indicate that hsa-miR-335-5p plays an important role in OA. Additionally, Yu et al. reported that exosomal hsa-miR-335-5p and hsa-miR-483-5p serve as novel biomarkers for rheumatoid arthritis (RA). It has been shown that RA is strongly associated with OA and sarcopenia (35–38). However, the association between hsa-mir-26b-5p, hsa-miR-335-5p, and OA or sarcopenia needs to be further explored.

Utilizing the DSigDB database, drug compounds were successfully identified. Out of the numerous predicted drugs, our study emphasized the top ten most promising candidates. Strophanthidin PC3UP, pimozide MCF7 UP, mastizole PC3 UP, niclosamide MCF7 UP, mebendazole HL60 UP, mefloquine MCF7 UP, tonzonium bromide PC3 UP, NICKEL CHLORIDE CTD 00001064, auststemizole MCF7 UP and calmidazolium MCF7 UP are the best candidates for the treatment of sarcopenia and OA. Our results suggest that the Stro-phanthidin PC3 UP is the best candidate for treating sarcopenia and OA. Strophanthidin is a positive inotropic drug. Since 1991, Vassalle has discovered the positive inotropic effects of low concentrations of strophanthidin (39). It can shorten the action potential time by inhibiting the sodium-potassium pump, thus exerting a positive inotropic effect (40). This gene may be an important regulator of human musculoskeletal growth and development. Pimozide is a psychotropic drug commonly used in the treatment of anxiety and depression. Some studies have demonstrated that pimozide can inhibit skeletal muscle ketone oxidation via SCOT/Oxct1, thereby may attenuating sarcopenia (41). However, it has also been shown that in mouse models of Amyotrophic Lateral Sclerosis (ALS), pimozide fails to attenuate motor and pathological deficits (42). Niclosamide is an antiparasitic drug identified by Zhang et al. (43) as a potential therapeutic agent for the treatment of OA and COVID-19. Additionally, studies (44) have shown that niclosamide activates FOXO3 in atrophied muscles. Its treatment inhibits myogenesis in muscle precursor cells, enhances the expression of myoblast markers Pax7 and Myf5, and downregulates the expression of differentiation markers MyoD, MyoG, and Myh2. Thus, niclosamide may be a potential treatment for sarcopenia and OA. Astemizole is an antihistamine with many applications in allergic and immunological diseases. Many studies have shown that OA and sarcopenia are associated with histamine (45, 46). Sarcopenia and OA may be prevented by antagonizing histamine formation to alleviate the patient’s inflammatory response.

To investigate the intricate interplay between the immune system and the concurrent occurrence of sarcopenia and osteoarthritis, immune infiltration analyses were conducted on datasets pertaining to both conditions. As shown in Figures 14, 15, we showed the degree of infiltration of different immune cells in the sarcopenia and OA datasets. The relationships between the hub genes and immune cells were analyzed and are visualized in these figures. We found that the sarcopenia group had lower activation. Dendritic.cell (*p* = 0.030) and regulatory.T.cell (*p* = 0.009) expression scores. In addition, the OA group had lower Eosinophi (*p* < 0.001), and Effector.memory.CD4.T.cell (*p* < 0.001), Central.memory.CD8.T.cell (*p* < 0.001), Effector.memory.CD8.T.cell (*p* = 0.002) and Type.2.T.helper.cell (*p* < 0.001) expression scores were obtained, while the Activated.B.cell (*p* = 0.002), Immature.B.cell (*p* < 0.001), Immature.dendritic.cell (*p* < 0.001), Macrophage (*p* = 0.002), Natural. killer. Cell (*p* < 0.001), Regulatory.T.cell (*p* < 0.001), Type.1.T.cell (*p* = 0.015) and Effector. memory.CD4.T.cell (*p* < 0.001) expression scores were also obtained. However, no significant differences were observed in the proportions of other immune cell subpopulations between the two groups. Subsequently, we evaluated the correlation between the hub genes and the infiltration patterns of various immune cells. The results showed a strong positive correlation (*p* < 0.01) between NFKBIA and effector.Memory.CD4.T.cell and memory.B.cell, while ZFP36 had a strong negative correlation with both eosinophils and type 2.T.helper.cell cells (*p* < 0.01) in the sarcopenia datasets. In addition, the results of the OA dataset showed that DDIT4 was strongly positively correlated with activated CD8.T.cell (*p* < 0.01), and NFKBIA was strongly positively correlated with mast. Cell (*p* < 0.01). However, ZFP36 had a strong negative correlation with Memory.B.cell (*p* < 0.01). Previous studies have shown that the immune system regulates muscle regeneration and growth and plays an important role in the progression of sarcopenia (47). Regulatory T cells, comprising a minor subpopulation of immune cells, function to restrain exaggerated immune activation and uphold immune homeostasis. In the context of sarcopenia, reduced Regulatory T cells function may contribute to chronic low-grade inflammation, exacerbating muscle degradation. Conversely, in osteoarthritis, dysregulation of Regulatory T cells activity could lead to persistent inflammation and joint degeneration. These cells possess the capability of serving as versatile and adaptable therapeutic agents for the management of inflammatory disorders (48). Increasing Regulatory T cells numbers or enhancing their suppressive functions may help mitigate inflammation and its detrimental effects on muscle and joint tissues. For instance, Maike et al. (49) reported that regulatory T cells can control skeletal muscle function and regeneration through IL6 receptor alpha signalling. IL-2 therapy has been explored to selectively expand Regulatory T cells in autoimmune diseases and could be repurposed for musculoskeletal conditions. Therefore, high expression of activated CD8 T cells is important for the treatment of sarcopenia and OA.

Although several previous studies have reported the relationships between OA or sarcopenia and hub genes (50–53), bioinformatics methods have not explored the common molecular mechanisms involved. We conducted a DEG analysis comparing the sarcopenia and OA databases to delve into the underlying mechanisms of interaction between these two diseases. Notably, various factors such as impaired insulin sensitivity, AGEs, obesity, and vitamin D deficiency may contribute to this association. Consequently, our study aims to provide valuable insights for the early diagnosis of sarcopenia in elderly patients with OA. Nevertheless, it is acknowledged that our research holds certain limitations. First, the data were downloaded from public databases, and the amount of data and information was limited and unbalanced. Given that most of the data are derived from cross-sectional analyses and lack time series samples, the collection of longitudinal data and the exploration of time series in sarcopenia and osteoarthritis, as ageing-related diseases, will be the focus of future analyses. Furthermore, even though sarcopenia and OA patients underwent differential and enrichment analyses, important genes that influence the course of these diseases may still be missing. Finally, in the future, we plan to collect muscle and synovial samples from patients with sarcopenia and OA to validate hub gene expression using Western Blot and RT-qPCR analyses. Additionally, we will conduct cohort studies to associate hub gene expression with patients having both OA and sarcopenia to confirm their prognostic value.

## 4 Methods

### 4.1 Dataset preparation

We downloaded the GSE1428 and GSE55235 datasets from the Gene Expression Omnibus (GEO)^1^ database (Table 1). The GSE1428 dataset included the global gene expression profiles of the vastus lateralis muscle of 10 young (19–25 years old) and 12 older (70–80 years old) male subjects from whom expression profiling was performed via an array. The GSE55235 dataset encompassed three multicenter genome-wide transcriptomic datasets (Affymetrix HG-U133 A/B) encompassing a total of 79 individuals. This cohort comprised 20 healthy controls (control group - CG), 26 patients with osteoarthritis (OA), and 33 patients with rheumatoid arthritis (RA). Utilizing this dataset, we inferred rule-based classifiers aimed at discriminating between the various disease groups. For our study, we exclusively utilized data from the OA group and the control group.

**Table 1:** Summary information of studies included in the analysis.

GEO accession	Author	Public date	Platform	Healthy:disease
GSE1428	Giresi PG	May 24, 2004	GPL96[HG-U133A] Affymetrix Human Genome U133A Array	10:12
GSE55235	Woetzel D	February 21, 2014	GPL96[HG-U133A] Affymetrix Human Genome U133A Array	20: 33

### 4.2 Identification of DEGs and shared DEGs between sarcopenia and OA patients

We used the *limma* package of R software (version 4.1.1) to select DEGs between sarcopenia patients and nonsarcopenia patients and between OA patients and healthy individuals (54). For sarcopenia data, *p* < 0.05 and |log fold change (FC)| > 0.585 were the criteria for screening DEGs. For the OA data, the criteria for screening out DEGs were set as *p* < 0.05 and |log fold change (FC)| > 1 (55). The *Pheatmap*, *ggplot2,* and *Venn. diagram* R package (version 4.1.1) was used to generate heatmaps and volcano plots and obtain the common DEGs between GSE1428 and GSE55235 (Table 2).

**Table 2:** 32 common DEGs.

Gene name	Protein name	Gene name	Protein name
OSBPL3	Oxysterol-binding protein-related protein 3	ZNF395	Zinc finger protein 395
SLC5A2	Sodium/glucose cotransporter 2	ANKRD11	Ankyrin repeat domain-containing protein 11
SCUBE2	Signal peptide, CUB and EGF-like domain-containing protein 2	ZBTB16	Zinc finger and BTB domain-containing protein 16
BTN3A3	Butyrophilin subfamily 3 member A3	MFAP5	Microfibrillar-associated protein 5
WWC1	Protein KIBRA	KLF13	Krueppel-like factor 13
JPT1	Jupiter microtubule associated homolog 1, N-terminally processed	ZFP36	mRNA decay activator protein ZFP36
LTB	Lymphotoxin-beta	DDIT4	DNA damage-inducible transcript 4 protein
CEBPB	CCAAT/enhancer-binding protein beta	CYP26B1	Cytochrome P450 26B1
NNMT	Nicotinamide N-methyltransferase	BCL6	B-cell lymphoma 6 protein
PUS1	tRNA pseudouridine synthase A	IER5	Immediate early response gene 5 protein
FOXO3	Forkhead box protein O3	IFRD1	Interferon-related developmental regulator 1
H1-10	Histone H1x	RRAD	GTP-binding protein RAD
CDKN1A	Cyclin-dependent kinase inhibitor 1	LGALSL	Galectin-related protein
NFKBIA	NF-kappa-B inhibitor alpha	FANCE	Fanconi anemia group E protein
ADM	Proadrenomedullin N-20 terminal peptide	RPGR	X-linked retinitis pigmentosa GTPase regulator
BTG2	Protein BTG2	FHL5	Four and a half LIM domains protein 5

### 4.3 Gene ontology and pathway enrichment analysis

Gene ontology (GO) (biological process, cellular component, and molecular function) and pathway enrichment analyses (WikiPathways, Reactome, BioCarta, and Kyoto Encyclopedia of Genes and Genomes [KEGG]) were also conducted using the *clusterProfiler*, *enrich plot*, *org.Hs.eg.db*, *ggplot2*, *circle*, *RColorBrewer*, *dplyr*, *ggpubr* and *ComplexHeatmap* packages of R software (version 4.1.1) to identify the functions and pathways shared between sarcopenia and OA. A *p* < 0.05 was considered to indicate statistical enrichment.

### 4.4 PPI network analysis

STRING (version 12.0, accessed via)^2^ serves as a comprehensive database for the exploration of protein–protein interaction (PPI) networks, boasting enhanced information coverage encompassing over 14,000 species, 67 million proteins, and 20 billion interactions. This extensive repository facilitates functional discovery within genome-wide experimental datasets. To construct the PPI network of proteins derived from the commonly shared differentially expressed genes (DEGs), we utilized the STRING database, setting an interaction score threshold of >0.15.

### 4.5 Identification and analysis of hub genes

Hub genes occupy a pivotal position as the most intricately interconnected nodes within a protein–protein interaction (PPI) network, which comprises nodes, edges, and their interconnectedness. Cytohubba (accessible via)^3^ serves as an innovative plugin within Cytoscape, designed to efficiently extract the central elements of biological networks (56). Seven algorithms (closeness, MCC, degree, MNC, radiality, stress, and EPC) were used to screen and intersect hub genes. GeneMANIA^4^ (57) is a flexible and useful website for gene function analysis and was used to construct a coexpression network of identified hub genes.

### 4.6 Construction of TF-gene and miRNA-gene regulatory networks

TFs regulate gene transcription, and miRNAs posttranscriptionally regulate gene expression; thus, understanding their activity is essential for obtaining molecular insights (58, 59). NetworkAnalyst^5^ is a broad online platform for web-based statistics, visualization, and meta-analysis of gene expression data (60). JASPAR^6^ is a public resource that provides spectral information across six taxonomic groups of multispecies TF combinations (61). MirTarBase is a valuable tool that aids researchers in screening for prominent microRNAs (miRNAs) and elucidating their biological functions and characteristics, thereby fostering the advancement of biological hypotheses. Utilizing the NetworkAnalyst platform, we topologically identified credible transcription factors (TFs) with a propensity to bind to our hub genes, drawing from the JASPAR database. Subsequently, we employed MirTarBase to screen for miRNAs that interact with our hub genes through NetworkAnalyst.

### 4.7 Gene-disease association analysis

DisGeNET serves as a comprehensive platform that integrates diverse information pertaining to genes and variants associated with human diseases. This resource offers a powerful tool for delving into the molecular underpinnings of specific human diseases and their associated complications, thereby enhancing our understanding of these conditions (62). Furthermore, to elucidate the associations between hub genes and their related diseases, as well as their complications, we utilized the DisGeNET database through the NetworkAnalyst platform to examine the gene-disease relationships.

### 4.8 Evaluation of applicant drugs

The Drug Signatures database (DSigDB), which contains 22,527 gene sets, was used to generate small molecules that could downregulate the expression of hub genes (63). We accessed the DSigDB database through “enrichment”^7^ (64). Based on the selected central genes, drug molecular identification was performed by enrichment using DSigDB.

### 4.9 ROC curves of the hub genes

The receiver operating characteristic (ROC) curves were plotted, and the area under the curve (AUC) was subsequently computed utilizing the pROC software package in R. This analysis was conducted to assess the diagnostic capabilities of each individual candidate hub gene in isolation.

### 4.10 Immune infiltration

To elucidate the role of the immune system in the skeletal and muscular systems, we conducted immune infiltration analyses. Utilizing the online platform CIBERSORTx,^8^ we were able to generate an immune infiltration matrix that captured the immune cell infiltration patterns within these systems. This matrix was subsequently imported into R software for visualization purposes, leveraging the powerful *ggplot2* package. Additionally, we employed the Wilcoxon test to statistically compare differences between the two groups and to visualize the correlation between 22 infiltrating immune cells and key genes. This comprehensive analysis provided insights into the intricate interactions between the immune system and the bone and muscle systems.

## 5 Conclusion

This study represents the inaugural investigation into the intricate relationship between sarcopenia and osteoarthritis through transcriptome analysis. Our objective was to identify key hub genes associated with both sarcopenia and osteoarthritis, thereby elucidating the potential link between these two conditions. The results showed that sarcopenia and OA share similar pathogeneses, such as transcriptional misregulation in cancer, the FOXO signalling pathway, and endometrial cancer. In addition, the presence of one disease may increase the risk of developing another. Moreover, we identified several drug targets based on the hub genes that may be candidates for drugs that are already sanctioned. We also investigated the relationships between immune cell levels and hub genes in patients with these two diseases through immune infiltration. In conclusion, our research offers novel biological targets and insights that could pave the way for earlier diagnosis and more effective treatment strategies for patients suffering from the combined condition of sarcopenia and OA.

## Data availability statement

Publicly available datasets were analyzed in this study. This data can be found at: https://www.ncbi.nlm.nih.gov/geo/, GSE1428 and GSE55235.

## Author contributions

JY: Conceptualization, Data curation, Formal analysis, Funding acquisition, Investigation, Methodology, Project administration, Resources, Software, Supervision, Validation, Visualization, Writing – original draft, Writing – review & editing. JZ: Conceptualization, Data curation, Formal analysis, Funding acquisition, Investigation, Methodology, Project administration, Resources, Software, Supervision, Validation, Visualization, Writing – original draft. YD: Conceptualization, Data curation, Formal analysis, Funding acquisition, Investigation, Methodology, Project administration, Resources, Software, Supervision, Validation, Visualization, Writing – original draft. ZW: Conceptualization, Data curation, Investigation, Methodology, Software, Supervision, Validation, Writing – original draft. LJ: Conceptualization, Data curation, Formal analysis, Methodology, Writing – original draft. ZL: Conceptualization, Data curation, Formal analysis, Funding acquisition, Investigation, Methodology, Project administration, Resources, Software, Supervision, Validation, Visualization, Writing – review & editing. YL: Conceptualization, Data curation, Formal analysis, Funding acquisition, Investigation, Methodology, Project administration, Resources, Software, Supervision, Validation, Visualization, Writing – review & editing.

## Footnotes

^1^http://www.Ncbi.nlm.Nih.gov/geo/

^2^http://string-db.org

^3^http://apps.cytoscape.org/apps/cytohubba

^4^https://genemanania.Org

^5^https://www.networkanalyst.ca/

^6^http://jaspar.Gener

^7^https://amp.pharm.org

^8^https://cibersortx.stanford.edu/

## References

[1] Cruz-Jentoft AJ Bahat G Bauer J Boirie Y Bruyère O Cederholm T. Sarcopenia: revised European consensus on definition and diagnosis. Age Ageing. (2019) 48:601. doi: 10.1093/ageing/afz046, PMID: 31081853 PMC6593317

[2] Shou J Chen PJ Xiao WH. Mechanism of increased risk of insulin resistance in ageing skeletal muscle. Diabetol Metab Syndr. (2020) 12:14. doi: 10.1186/s13098-020-0523-x, PMID: 32082422 PMC7014712

[3] Rosenberg IH. Sarcopenia: origins and clinical relevance. J Nutr. (1997) 127:990S (5 Suppl), 990 s991 s–1S. doi: 10.1093/jn/127.5.990S 9164280

[4] Cruz-Jentoft AJ Sayer AA. Sarcopenia. Lancet. (2019) 393:2636–46. doi: 10.1016/S0140-6736(19)31138-9 31171417

[5] Mayhew AJ Amog K Phillips S Parise G McNicholas PD de Souza RJ. The prevalence of sarcopenia in community-dwelling older adults, an exploration of differences between studies and within definitions: a systematic review and meta-analyses. Age Ageing. (2019) 48:48–56. doi: 10.1093/ageing/afy106, PMID: 30052707

[6] Chen LK Liu LK Woo J Assantachai P Auyeung TW Bahyah KS. Sarcopenia in Asia: consensus report of the Asian working Group for Sarcopenia. J Am Med Dir Assoc. (2014) 15:95–101. doi: 10.1016/j.jamda.2013.11.025 24461239

[7] Petermann-Rocha F Balntzi V Gray SR Lara J Ho FK Pell JP. Global prevalence of sarcopenia and severe sarcopenia: a systematic review and meta-analysis. J Cachexia Sarcopenia Muscle. (2022) 13:86–99. doi: 10.1002/jcsm.12783, PMID: 34816624 PMC8818604

[8] Janssen I Shepard DS Katzmarzyk PT Roubenoff R. The healthcare costs of sarcopenia in the United States. J Am Geriatr Soc. (2004) 52:80–5. doi: 10.1111/j.1532-5415.2004.52014.x 14687319

[9] Bruyère O Beaudart C Ethgen O Reginster JY Locquet M. The health economics burden of sarcopenia: a sys-tematic review. Maturitas. (2019) 119:61–9. doi: 10.1016/j.maturitas.2018.11.003, PMID: 30502752

[10] Hunter DJ Bierma-Zeinstra S. Osteoarthritis. Lancet. (2019) 393:1745–59. doi: 10.1016/S0140-6736(19)30417-9 31034380

[11] Peat G McCarney R Croft P. Knee pain and osteoarthritis in older adults: a review of community burden and current use of primary health care. Ann Rheum Dis. (2001) 60:91–7. doi: 10.1136/ard.60.2.91, PMID: 11156538 PMC1753462

[12] Glyn-Jones S Palmer AJ Agricola R Price AJ Vincent TL Weinans H. Osteoarthritis. Lancet. (2015) 386:376–87. doi: 10.1016/S0140-6736(14)60802-3 25748615

[13] Losina E Walensky RP Reichmann WM Holt HL Gerlovin H Solomon DH. Impact of obesity and knee osteoarthritis on morbidity and mortality in older Americans. Ann Intern Med. (2011) 154:217–26. doi: 10.7326/0003-4819-154-4-201102150-00001 21320937 PMC3260464

[14] Felson DT Lawrence RC Dieppe PA Hirsch R Helmick CG Jordan JM. Osteoarthritis: new insights. Part 1: the disease and its risk factors. Ann Intern Med. (2000) 133:635–46. doi: 10.7326/0003-4819-133-8-200010170-00016, PMID: 11033593

[15] Øiestad BE Juhl CB Eitzen I Thorlund JB. Knee extensor muscle weakness is a risk factor for the development of knee osteoarthritis. A systematic review and meta-analysis. Osteoarthr Cartil. (2015) 23:171–7. doi: 10.1016/j.joca.2014.10.008, PMID: 25450853

[16] Karlsson MK Magnusson H Cöster M Karlsson C Rosengren BE. Patients with knee osteoarthritis have a phenotype with higher bone mass, higher fat mass, and lower lean body mass. Clin Orthop Relat Res. (2015) 473:258–64. doi: 10.1007/s11999-014-3973-3, PMID: 25280553 PMC4390976

[17] Chung SM Hyun MH Lee E Seo HS. Novel effects of sarcopenic osteoarthritis on metabolic syndrome, insulin resistance, osteoporosis, and bone fracture: the national survey. Osteoporos Int. (2016) 27:2447–57. doi: 10.1007/s00198-016-3548-0, PMID: 27177746

[18] Godziuk K Prado CM Woodhouse LJ Forhan M. The impact of sarcopenic obesity on knee and hip osteoarthritis: a scoping review. BMC Musculoskelet Disord. (2018) 19:271. doi: 10.1186/s12891-018-2175-7, PMID: 30055599 PMC6064616

[19] Ho KK Lau LC Chau WW Poon Q Chung KY Wong RM. End-stage knee osteoarthritis with and without sarcopenia and the effect of knee arthroplasty - a prospective cohort study. BMC Geriatr. (2021) 21:2. doi: 10.1186/s12877-020-01929-6, PMID: 33397330 PMC7784022

[20] Liu Y Huang C Bai M Pi C Zhang D Xie J. The roles of Runx1 in skeletal development and osteoarthritis: a concise review. Heliyon. (2022) 8:e12656. doi: 10.1016/j.heliyon.2022.e12656, PMID: 36636224 PMC9830174

[21] Ding P Gao C Gao Y Liu D Li H Xu J. Osteocytes regulate senescence of bone and bone marrow. eLife. (2022) 11:11. doi: 10.7554/eLife.81480 PMC9678362 36305580

[22] Vervoort SJ Devlin JR Kwiatkowski N Teng M Gray NS Johnstone RW. Targeting transcription cycles in cancer. Nat Rev Cancer. (2022) 22:5–24. doi: 10.1038/s41568-021-00411-8 34675395

[23] Liu PJ Hu YS Wang MJ Kang L. Nutrient weight against sarcopenia: regulation of the IGF-1/PI3K/Akt/FOXO pathway in quinoa metabolites. Curr Opin Pharmacol. (2021) 61:136–41. doi: 10.1016/j.coph.2021.10.001, PMID: 34801804

[24] Park SS Seo YK Kwon KS. Sarcopenia targeting with autophagy mechanism by exercise. BMB Rep. (2019) 52:64–9. doi: 10.5483/BMBRep.2019.52.1.292, PMID: 30526769 PMC6386226

[25] Zhao C Li X Sun G Liu P Kong K Chen X. CircFOXO3 protects against osteoarthritis by targeting its parental gene FOXO3 and activating PI3K/AKT-mediated autophagy. Cell Death Dis. (2022) 13:932. doi: 10.1038/s41419-022-05390-8, PMID: 36344492 PMC9640610

[26] Liu Y Zhou T Wang Q Fu R Zhang Z Chen N. M(6) a demethylase ALKBH5 drives denervation-induced muscle atrophy by targeting HDAC4 to activate FoxO3 signalling. J Cachexia Sarcopenia Muscle. (2022) 13:1210–23. doi: 10.1002/jcsm.12929, PMID: 35142084 PMC8978003

[27] Caramori G Casolari P Adcock I. Role of transcription factors in the pathogenesis of asthma and COPD. Cell Commun Adhes. (2013) 20:21–40. doi: 10.3109/15419061.2013.775257 23472830

[28] Sethupathy P Corda B Hatzigeorgiou AG. TarBase: a comprehensive database of experimentally supported animal microRNA targets. RNA. (2006) 12:192–7. doi: 10.1261/rna.2239606, PMID: 16373484 PMC1370898

[29] Zhang HM Kuang S Xiong X Gao T Liu C Guo AY. Transcription factor and microRNA coregulatory loops: important regulatory motifs in biological processes and diseases. Brief Bioinform. (2015) 16:45–58. doi: 10.1093/bib/bbt085, PMID: 24307685

[30] Wang J Wang Y Zhang H Gao W Lu M Liu W. Forkhead box C1 promotes the pathology of osteoarthritis by upregulating β-catenin in synovial fibroblasts. FEBS J. (2020) 287:3065–87. doi: 10.1111/febs.15178, PMID: 31837247

[31] He X Deng L. miR-204-5p inhibits inflammation of synovial fibroblasts in osteoarthritis by suppressing FOXC1. J Orthop Sci. (2022) 27:921–8. doi: 10.1016/j.jos.2021.03.014, PMID: 34045139

[32] Zhang Z Yang B Zhou S Wu J. CircRNA circ_SEC24A upregulates DNMT3A expression by sponging miR-26b-5p to aggravate osteoarthritis progression. Int Immunopharmacol. (2021) 99:107957. doi: 10.1016/j.intimp.2021.107957, PMID: 34325283

[33] Zhang MF Yang P Shen MY Wang X Gao NX Zhou XP. MicroRNA-26b-5p alleviates murine collagen-induced arthritis by modulating Th17 cell plasticity. Cell Immunol. (2021) 365:104382. doi: 10.1016/j.cellimm.2021.104382, PMID: 34049010

[34] Tornero-Esteban P Rodríguez-Rodríguez L Abásolo L Tomé M López-Romero P Herranz E. Signature of microRNA expression during osteogenic differentiation of bone marrow MSCs reveals a putative role of miR-335-5p in osteoarthritis. BMC Musculoskelet Disord. (2015) 16:182. doi: 10.1186/s12891-015-0652-9, PMID: 26243143 PMC4526194

[35] Bennett JL Pratt AG Dodds R Sayer AA Isaacs JD. Rheumatoid sarcopenia: loss of skeletal muscle strength and mass in rheumatoid arthritis. Nat Rev Rheumatol. (2023) 19:239–51. doi: 10.1038/s41584-023-00921-9, PMID: 36801919

[36] Ge Y Chen Z Fu Y Xiao X Xu H Shan L. Identification and validation of hub genes of synovial tissue for patients with osteoarthritis and rheumatoid arthritis. Hereditas. (2021) 158:37. doi: 10.1186/s41065-021-00201-0, PMID: 34583778 PMC8480049

[37] Wang J Fan Q Yu T Zhang Y. Identifying the hub genes and immune cell infiltration in synovial tissue between osteoarthritic and rheumatoid arthritic patients by Bioinformatic approach. Curr Pharm Des. (2022) 28:497–509. doi: 10.2174/1381612827666211104154459, PMID: 34736376

[38] Vlietstra L Stebbings S Meredith-Jones K Abbott JH Treharne GJ Waters DL. Sarcopenia in osteoarthritis and rheumatoid arthritis: the association with self-reported fatigue, physical function, and obesity. PLoS One. (2019) 14:e0217462. doi: 10.1371/journal.pone.0217462, PMID: 31170172 PMC6553728

[39] Aceto E Vassalle M. On the mechanism of the positive inotropy of low concentrations of strophanthidin. J Pharmacol Exp Ther. (1991) 259:182–9. PMID: 1920115

[40] Levi AJ. A role for sodium/calcium exchange in the action potential shortening caused by strophanthidin in guinea pig ventricular myocytes. Cardiovasc Res. (1993) 27:471–81. doi: 10.1093/cvr/27.3.471, PMID: 8387887

[41] al Batran R Gopal K Capozzi ME Chahade JJ Saleme B Tabatabaei-Dakhili SA. Pimozide alleviates hyperglycemia in diet-induced obesity by inhibiting skeletal muscle ketone oxidation. Cell Metab. (2020) 31:909–919.e8. doi: 10.1016/j.cmet.2020.03.017, PMID: 32275862

[42] Pozzi S Thammisetty SS Julien JP. Chronic administration of pimozide fails to attenuate motor and pathological deficits in two mouse models of amyotrophic lateral sclerosis. Neurotherapeutics. (2018) 15:715–27. doi: 10.1007/s13311-018-0634-3, PMID: 29790082 PMC6095790

[43] Zhang Y Duan Z Guan Y Xu T Fu Y Li G. Identification of 3 key genes as novel diagnostic and therapeutic targets for OA and COVID-19. Front Immunol. (2023) 14:1167639. doi: 10.3389/fimmu.2023.1167639, PMID: 37283761 PMC10239847

[44] Kim HJ Lee JH Kim SW Lee SH Jung DW Williams DR. Investigation of niclosamide as a repurposing agent for skeletal muscle atrophy. PLoS One. (2021) 16:e0252135. doi: 10.1371/journal.pone.0252135, PMID: 34038481 PMC8153455

[45] Apolloni S Amadio S Fabbrizio P Morello G Spampinato AG Latagliata EC. Histaminergic transmission slows progression of amyotrophic lateral sclerosis. J Cachexia Sarcopenia Muscle. (2019) 10:872–93. doi: 10.1002/jcsm.12422, PMID: 31020811 PMC6711424

[46] Zhao X Younis S Shi H Hu S Zia A Wong HH. RNA-seq characterization of histamine-releasing mast cells as potential therapeutic target of osteoarthritis. Clin Immunol. (2022) 244:109117. doi: 10.1016/j.clim.2022.109117, PMID: 36109004 PMC10752578

[47] Tidball JG. Regulation of muscle growth and regeneration by the immune system. Nat Rev Immunol. (2017) 17:165–78. doi: 10.1038/nri.2016.150, PMID: 28163303 PMC5452982

[48] Ferreira LMR Muller YD Bluestone JA Tang Q. Next-generation regulatory T-cell therapy. Nat Rev Drug Discov. (2019) 18:749–69. doi: 10.1038/s41573-019-0041-4, PMID: 31541224 PMC7773144

[49] Becker M Joseph SS Garcia-Carrizo F Tom RZ Opaleva D Serr I. Regulatory T cells require IL6 receptor alpha signaling to control skeletal muscle function and regeneration. Cell Metab. (2023) 35:1736–1751.e7. doi: 10.1016/j.cmet.2023.08.010, PMID: 37734370 PMC10563138

[50] Misra D Fielding RA Felson DT Niu J Brown C Nevitt M. Risk of knee osteoarthritis with obesity, sarcopenic obesity, and sarcopenia. Arthritis Rheumatol. (2019) 71:232–7. doi: 10.1002/art.40692, PMID: 30106249 PMC6374038

[51] Peng H Zeng Y. Research progress on the correlation between sarcopenia and osteoarthritis. Zhongguo Xiu Fu Chong Jian Wai Ke Za Zhi. (2022) 36:1549–57. doi: 10.7507/1002-1892.202209015, PMID: 36545865 PMC9763072

[52] Papalia R Zampogna B Torre G Lanotte A Vasta S Albo E. Sarcopenia and its relationship with osteoarthritis: risk factor or direct consequence? Musculoskelet Surg. (2014) 98:9–14. doi: 10.1007/s12306-014-0311-6 24482109

[53] Pegreffi F Balestra A De Lucia O Smith L Barbagallo M Veronese N. Prevalence of sarcopenia in knee Os-teoarthritis: a systematic review and Meta-analysis. J Clin Med. (2023) 12:1532. doi: 10.3390/jcm12041532, PMID: 36836065 PMC9963114

[54] Ritchie ME Phipson B Wu D Hu Y Law CW Shi W. Limma powers differential expression analyses for RNA-sequencing and microarray studies. Nucleic Acids Res. (2015) 43:e47. doi: 10.1093/nar/gkv007, PMID: 25605792 PMC4402510

[55] Davis S Meltzer PS. GEOquery: a bridge between the gene expression omnibus (GEO) and BioConductor. Bioinformatics. (2007) 23:1846–7. doi: 10.1093/bioinformatics/btm254, PMID: 17496320

[56] Chin CH Chen SH Wu HH Ho CW Ko MT Lin CY. cytoHubba: identifying hub objects and subnetworks from complex interactome. BMC Syst Biol. (2014) 8:S11. doi: 10.1186/1752-0509-8-S4-S11 25521941 PMC4290687

[57] Franz M Rodriguez H Lopes C Zuberi K Montojo J Bader GD. GeneMANIA update 2018. Nucleic Acids Res. (2018) 46:W60–w64. doi: 10.1093/nar/gky311 29912392 PMC6030815

[58] Cai Y Yu X Hu S Yu J. A brief review on the mechanisms of miRNA regulation. Genomics Proteomics Bioinformatics. (2009) 7:147–54. doi: 10.1016/S1672-0229(08)60044-3, PMID: 20172487 PMC5054406

[59] Lambert SA Jolma A Campitelli LF Das PK Yin Y Albu M. The human transcription factors. Cell. (2018) 172:650–65. doi: 10.1016/j.cell.2018.01.029 29425488 PMC12908702

[60] Xia J Gill EE Hancock RE. NetworkAnalyst for statistical, visual and network-based meta-analysis of gene expression data. Nat Protoc. (2015) 10:823–44. doi: 10.1038/nprot.2015.052 25950236

[61] Khan A Fornes O Stigliani A Gheorghe M Castro-Mondragon JA van der Lee R. JASPAR 2018: update of the open-access database of transcription factor binding profiles and its web framework. Nucleic Acids Res. (2018) 46:D260–d266. doi: 10.1093/nar/gkx1126, PMID: 29140473 PMC5753243

[62] Piñero J Bravo À Queralt-Rosinach N Gutiérrez-Sacristán A Deu-Pons J Centeno E. DisGeNET: a comprehensive platform integrating information on human disease-associated genes and variants. Nucleic Acids Res. (2017) 45:D833–d839. doi: 10.1093/nar/gkw943, PMID: 27924018 PMC5210640

[63] Yoo M Shin J Kim J Ryall KA Lee K Lee S. DSigDB: drug signatures database for gene set analysis. Bioinformatics. (2015) 31:3069–71. doi: 10.1093/bioinformatics/btv313, PMID: 25990557 PMC4668778

[64] Kuleshov MV Jones MR Rouillard AD Fernandez NF Duan Q Wang Z. Enrichr: a comprehensive gene set enrichment analysis web server 2016 update. Nucleic Acids Res. (2016) 44:W90–7. doi: 10.1093/nar/gkw377, PMID: 27141961 PMC4987924

