# Supplementary material for: Bioinformatics and systems biology approaches to identify potential common pathogeneses for sarcopenia and osteoarthritis
Source: Front Med (Lausanne). 2024 Jun 18;11:1380210. doi: 10.3389/fmed.2024.1380210 (PMC11221828; doi:10.3389/fmed.2024.1380210)
Supplement: Supplementary file 1 [file Table_1.DOCX]

| Gene name | Protein name | Gene name | Protein name |
| --- | --- | --- | --- |
| OSBPL3 | Oxysterol-binding protein-related protein 3 | ZNF395 | Zinc finger protein 395 |
| SLC5A2 | Sodium/glucose cotransporter 2 | ANKRD11 | Ankyrin repeat domain-containing protein 11 |
| SCUBE2 | Signal peptide, CUB and EGF-like domain-containing protein 2 | ZBTB16 | Zinc finger and BTB domain-containing protein 16 |
| BTN3A3 | Butyrophilin subfamily 3 member A3 | MFAP5 | Microfibrillar-associated protein 5 |
| WWC1 | Protein KIBRA | KLF13 | Krueppel-like factor 13 |
| JPT1 | Jupiter microtubule associated homolog 1, N-terminally processed | ZFP36 | mRNA decay activator protein ZFP36 |
| LTB | Lymphotoxin-beta | DDIT4 | DNA damage-inducible transcript 4 protein |
| CEBPB | CCAAT/enhancer-binding protein beta | CYP26B1 | Cytochrome P450 26B1 |
| NNMT | Nicotinamide N-methyltransferase | BCL6 | B-cell lymphoma 6 protein |
| PUS1 | tRNA pseudouridine synthase A | IER5 | Immediate early response gene 5 protein |
| FOXO3 | Forkhead box protein O3 | IFRD1 | Interferon-related developmental regulator 1 |
| H1-10 | Histone H1x | RRAD | GTP-binding protein RAD |
| CDKN1A | Cyclin-dependent kinase inhibitor 1 | LGALSL | Galectin-related protein |
| NFKBIA | NF-kappa-B inhibitor alpha | FANCE | Fanconi anemia group E protein |
| ADM | Proadrenomedullin N-20 terminal peptide | RPGR | X-linked retinitis pigmentosa GTPase regulator |
| BTG2 | Protein BTG2 | FHL5 | Four and a half LIM domains protein 5 |

32 common DEGs
