# Supplementary material for: Bioinformatics and systems biology approaches to identify potential common pathogeneses for sarcopenia and osteoarthritis
Source: Front Med (Lausanne). 2024 Jun 18;11:1380210. doi: 10.3389/fmed.2024.1380210 (PMC11221828; doi:10.3389/fmed.2024.1380210)
Supplement: Supplementary file 2 [file Table_2.DOCX]

| GEO accession | Author | Public date | Platform | Healthy:disease |
| --- | --- | --- | --- | --- |
| GSE1428 | Giresi PG | May24, 2004 | GPL96[HG-U133A] Affymetrix Human Genome U133A Array | 10:12 |
| GSE55235 | Woetzel D | Feb21, 2014 | GPL96[HG-U133A] Affymetrix Human Genome U133A Array | 20 :33 |

Summary information of studies included in analysis
