# Supplementary figures and images for: Bioinformatics and systems biology approaches to identify potential common pathogeneses for sarcopenia and osteoarthritis
Source: Front Med (Lausanne). 2024 Jun 18;11:1380210. doi: 10.3389/fmed.2024.1380210 (PMC11221828; doi:10.3389/fmed.2024.1380210)

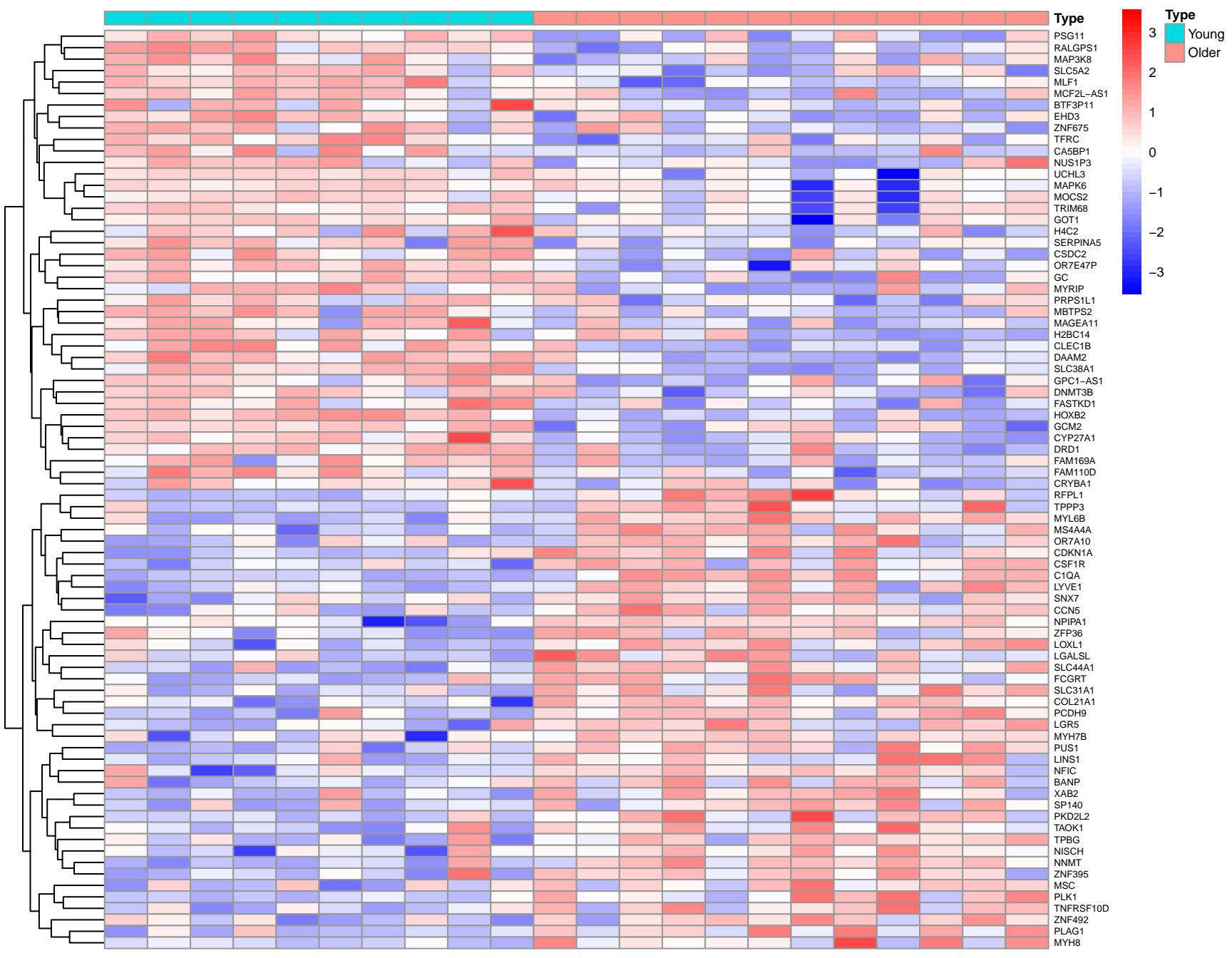

Supplement: Supplementary file 3 [file Data_Sheet_1.ZIP › raw data(1)/05.diff/GSE1428/GSE1428.heatmap.pdf]

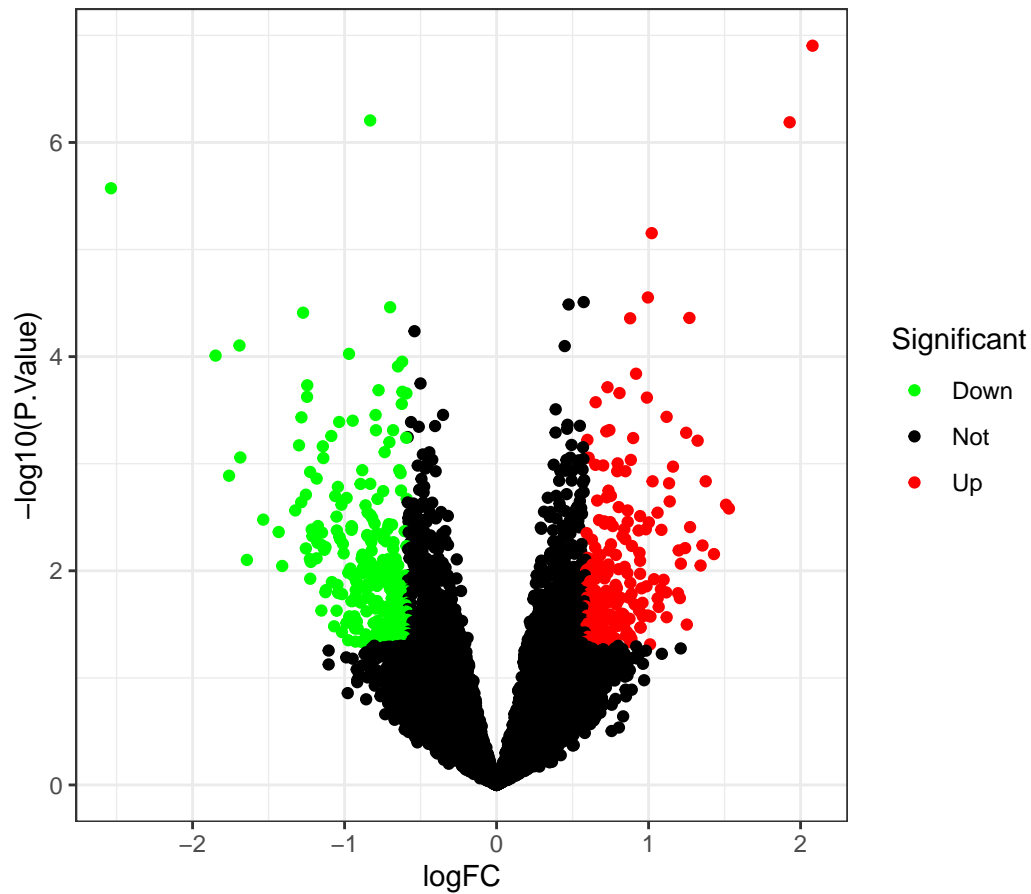

Supplement: Supplementary file 3 [file Data_Sheet_1.ZIP › raw data(1)/05.diff/GSE1428/GSE1428.vol.pdf]

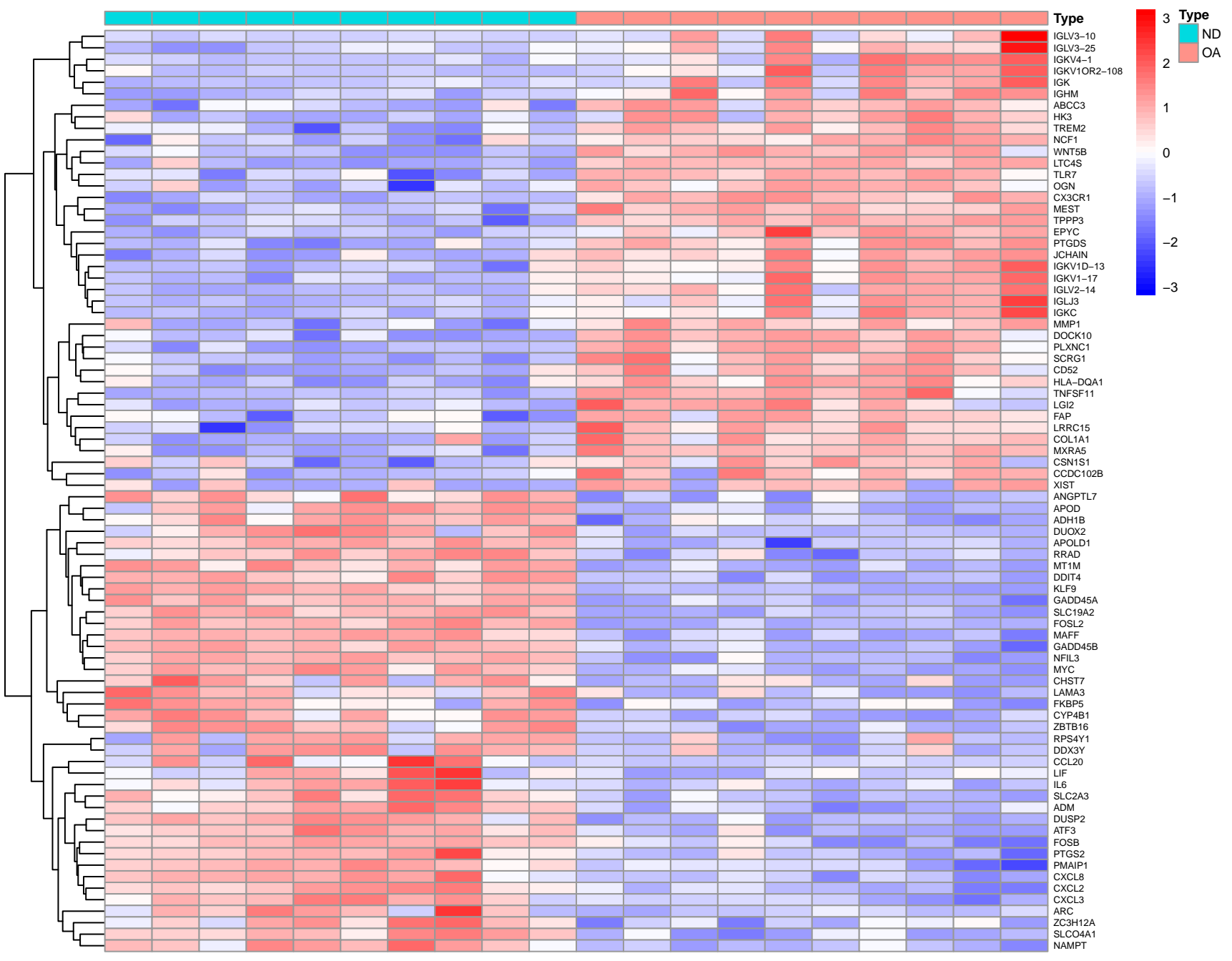

Supplement: Supplementary file 3 [file Data_Sheet_1.ZIP › raw data(1)/05.diff/GSE55235/GSE55235.heatmap.pdf]

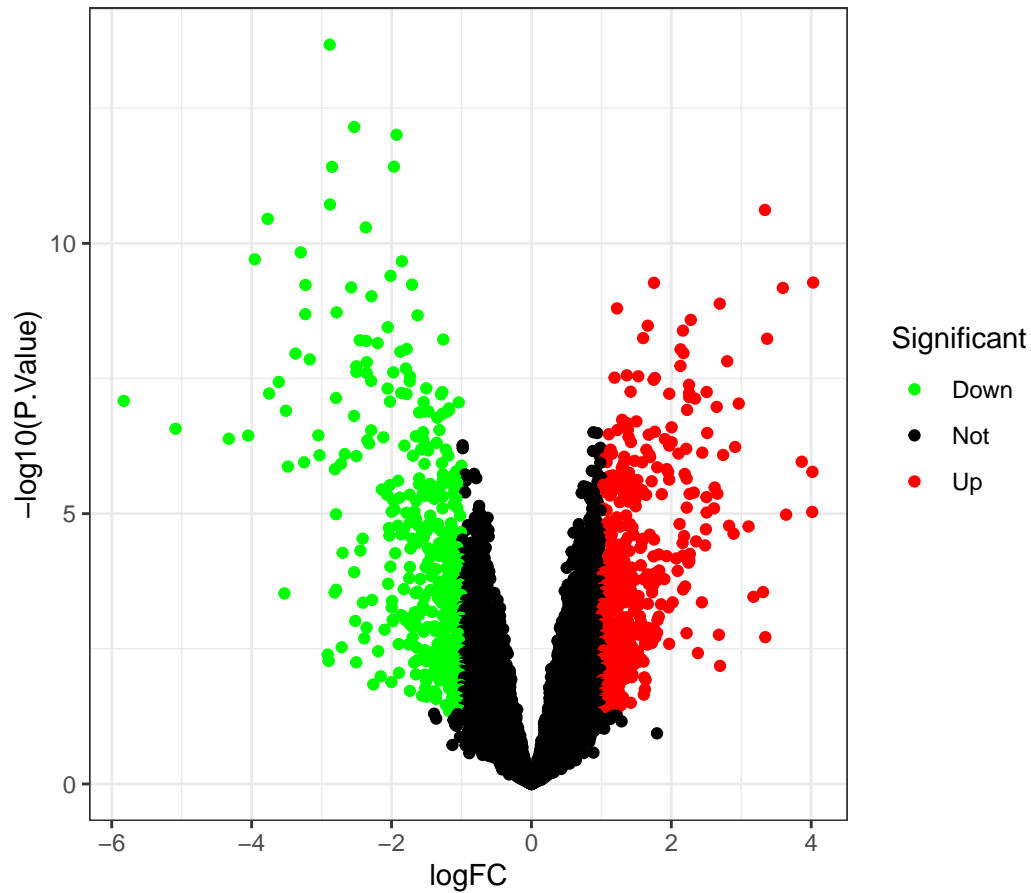

Supplement: Supplementary file 3 [file Data_Sheet_1.ZIP › raw data(1)/05.diff/GSE55235/GSE55235.vol.pdf]

GSE55235\_down

GSE1428\_down

369

25

229

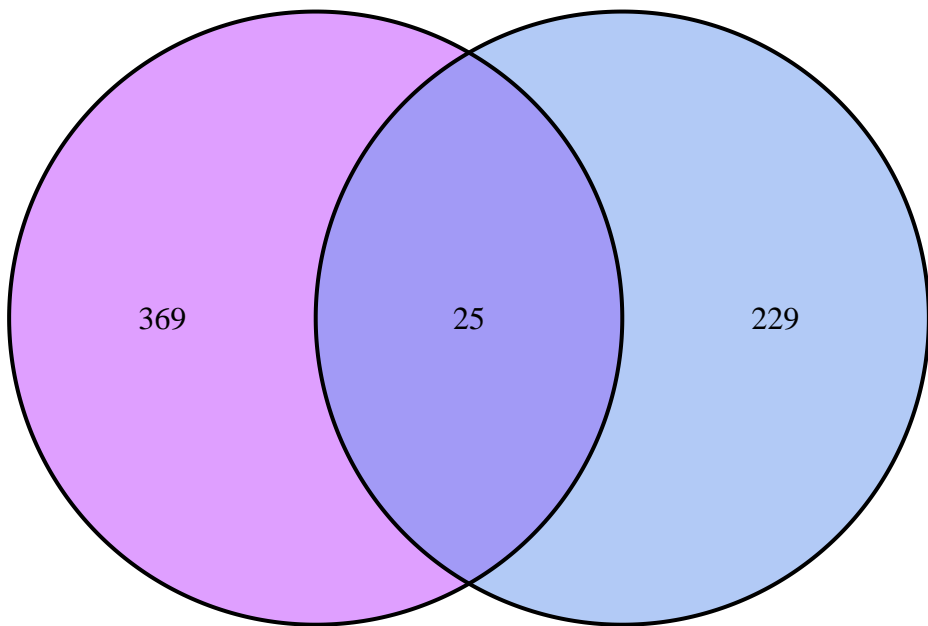

Supplement: Supplementary file 3 [file Data_Sheet_1.ZIP › raw data(1)/06.venn/down.pdf]

GSE55235\_up

GSE1428\_up

450

7

199

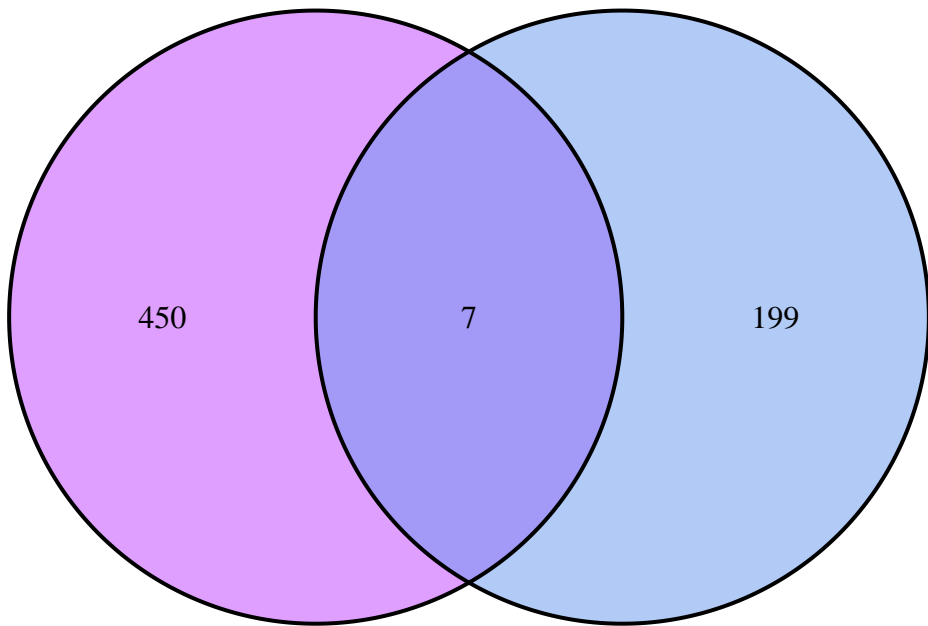

Supplement: Supplementary file 3 [file Data_Sheet_1.ZIP › raw data(1)/06.venn/up.pdf]

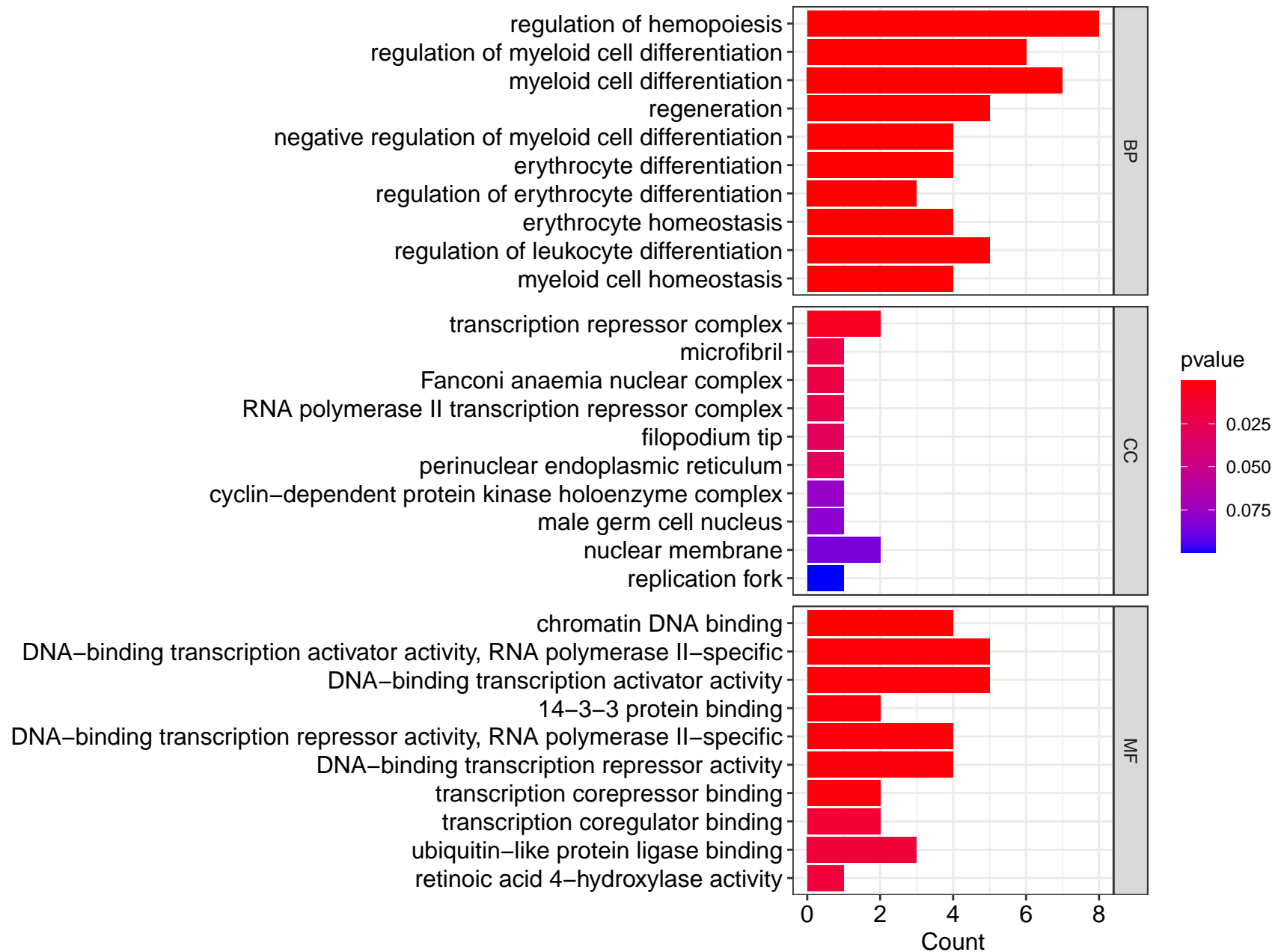

Supplement: Supplementary file 3 [file Data_Sheet_1.ZIP › raw data(1)/07.GO/barplot.pdf]

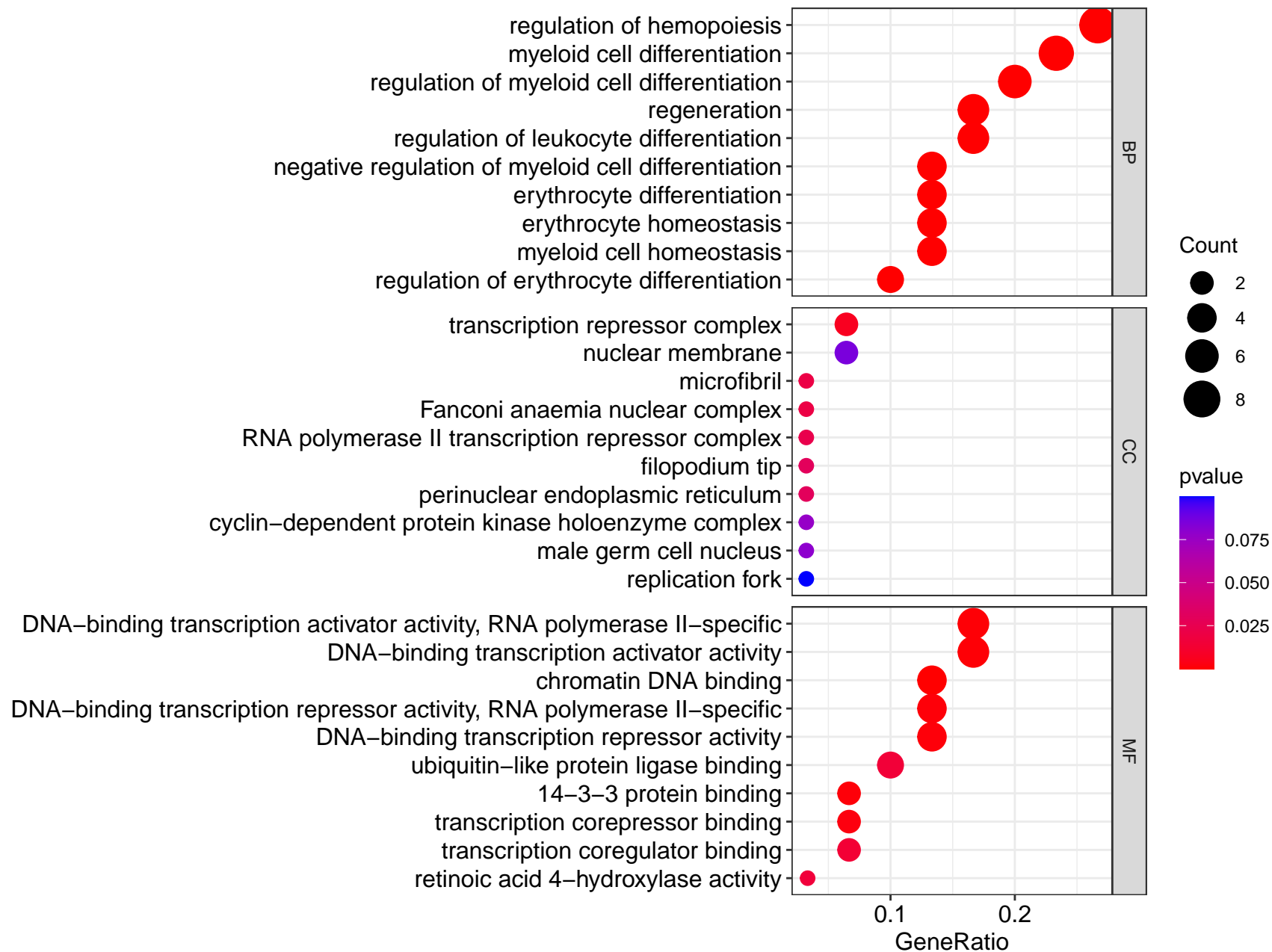

Supplement: Supplementary file 3 [file Data_Sheet_1.ZIP › raw data(1)/07.GO/bubble.pdf]

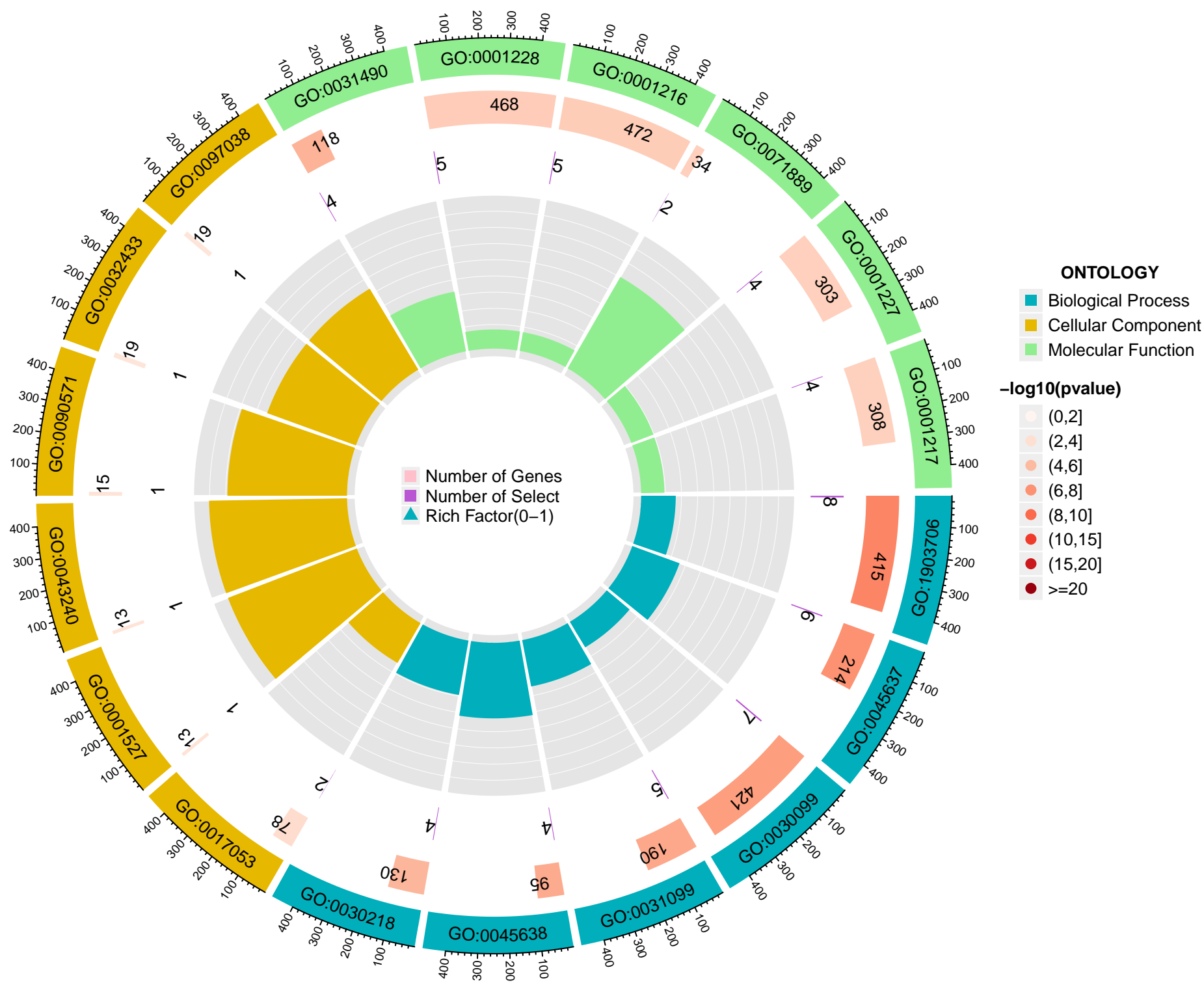

Supplement: Supplementary file 3 [file Data_Sheet_1.ZIP › raw data(1)/07.GO/GO.circlize.pdf]

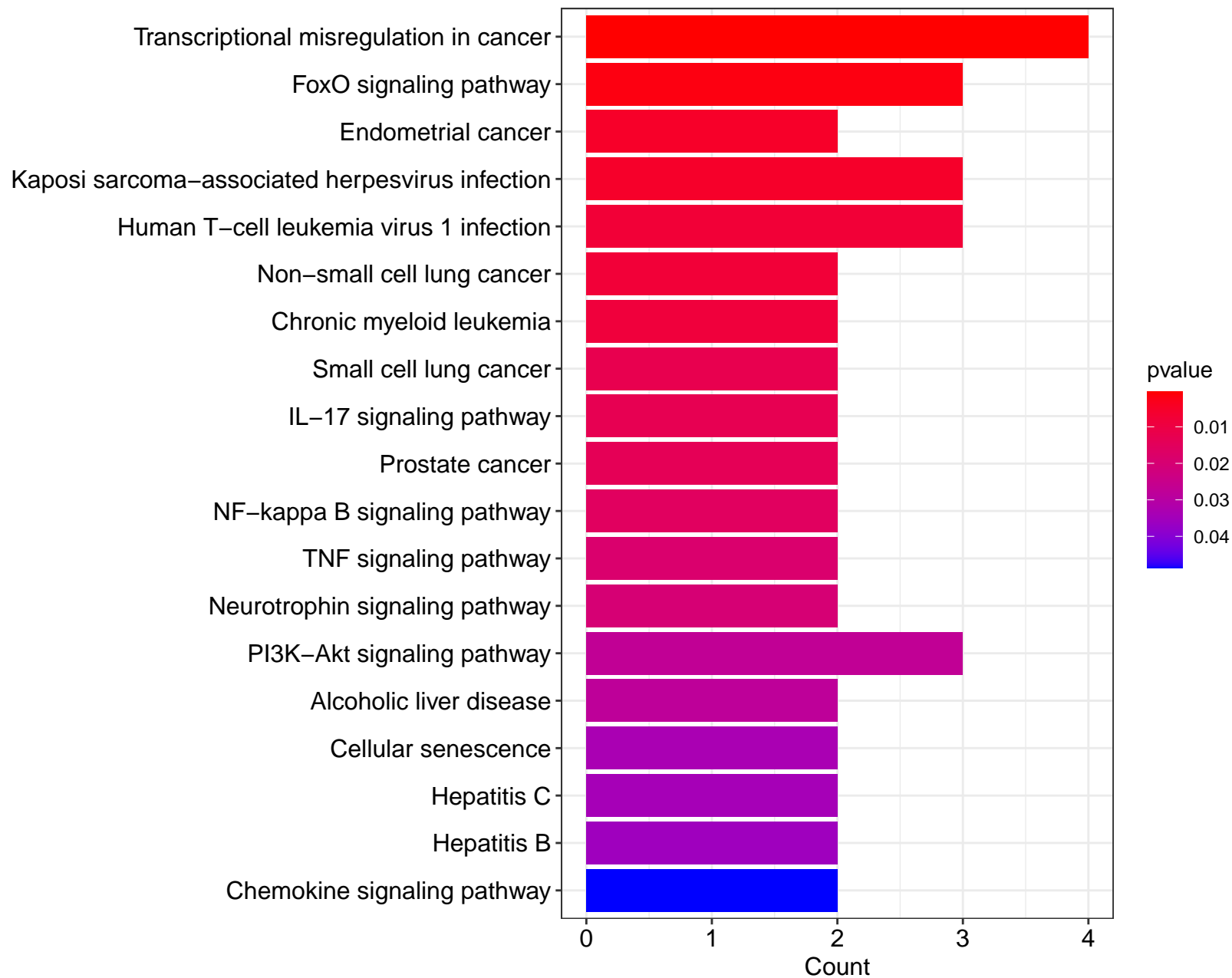

Supplement: Supplementary file 3 [file Data_Sheet_1.ZIP › raw data(1)/08.KEGG/barplot.pdf]

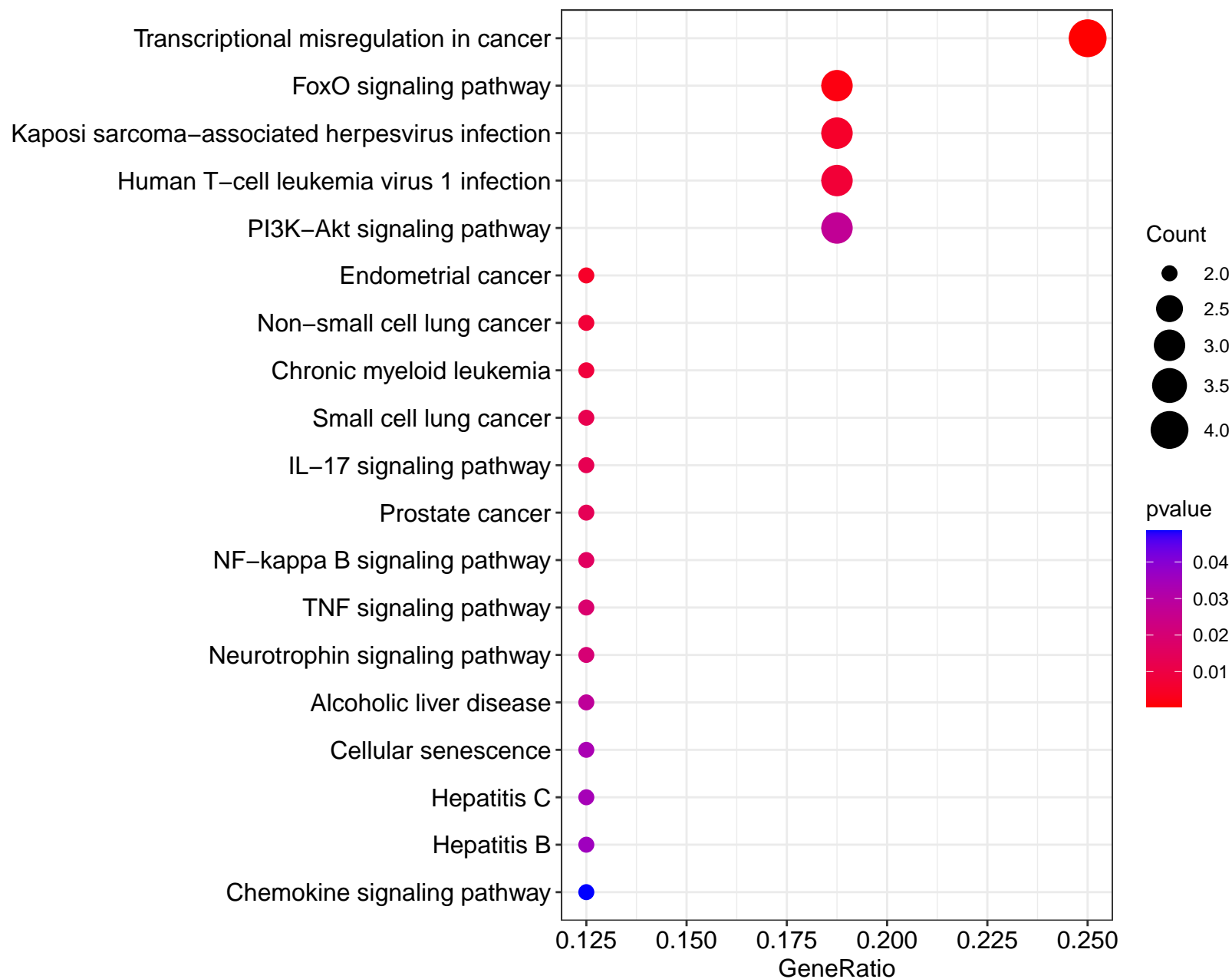

Supplement: Supplementary file 3 [file Data_Sheet_1.ZIP › raw data(1)/08.KEGG/bubble.pdf]

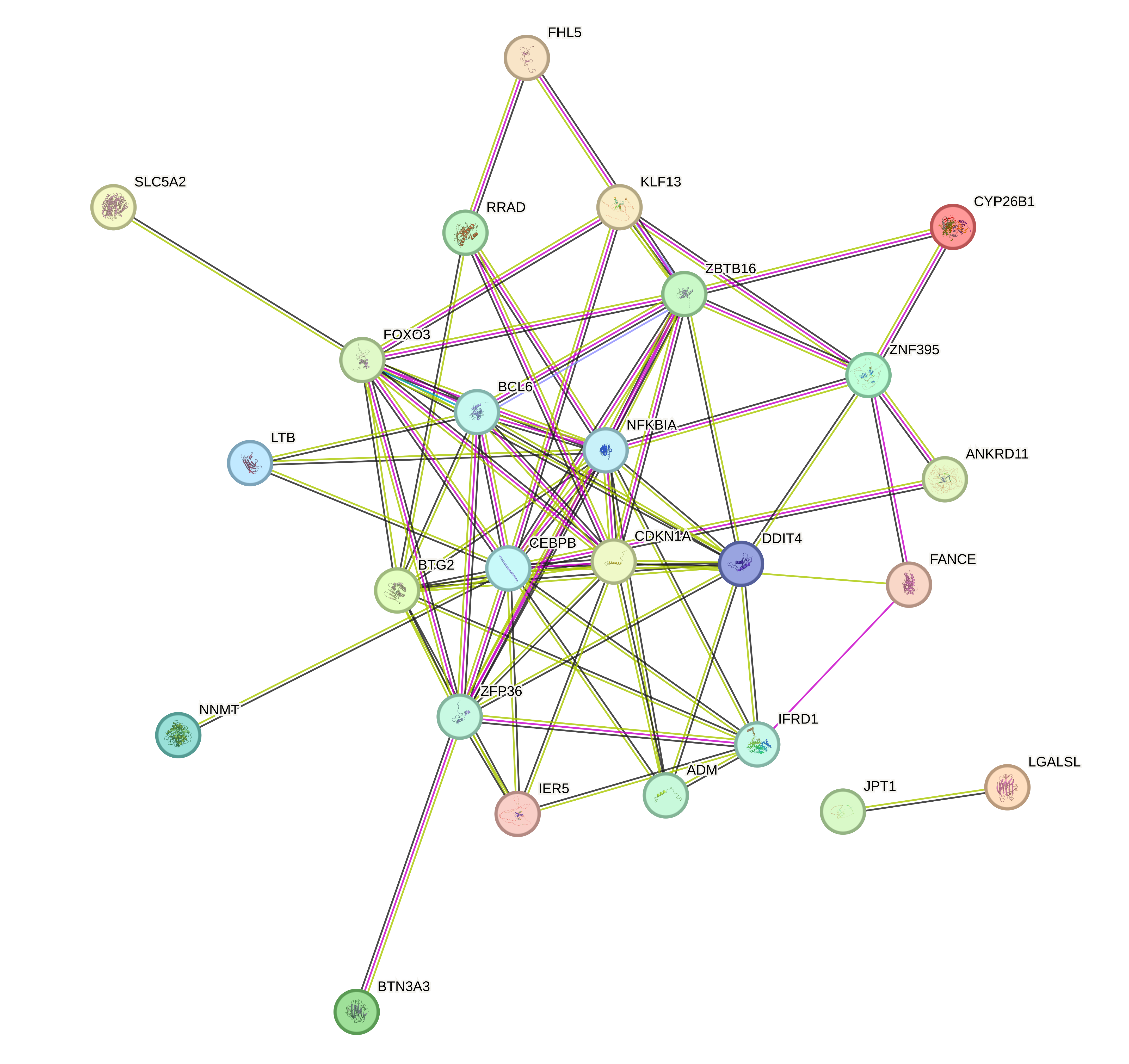

Supplement: Supplementary file 3 [file Data_Sheet_1.ZIP › raw data(1)/09.PPI/string_hires_image.png]

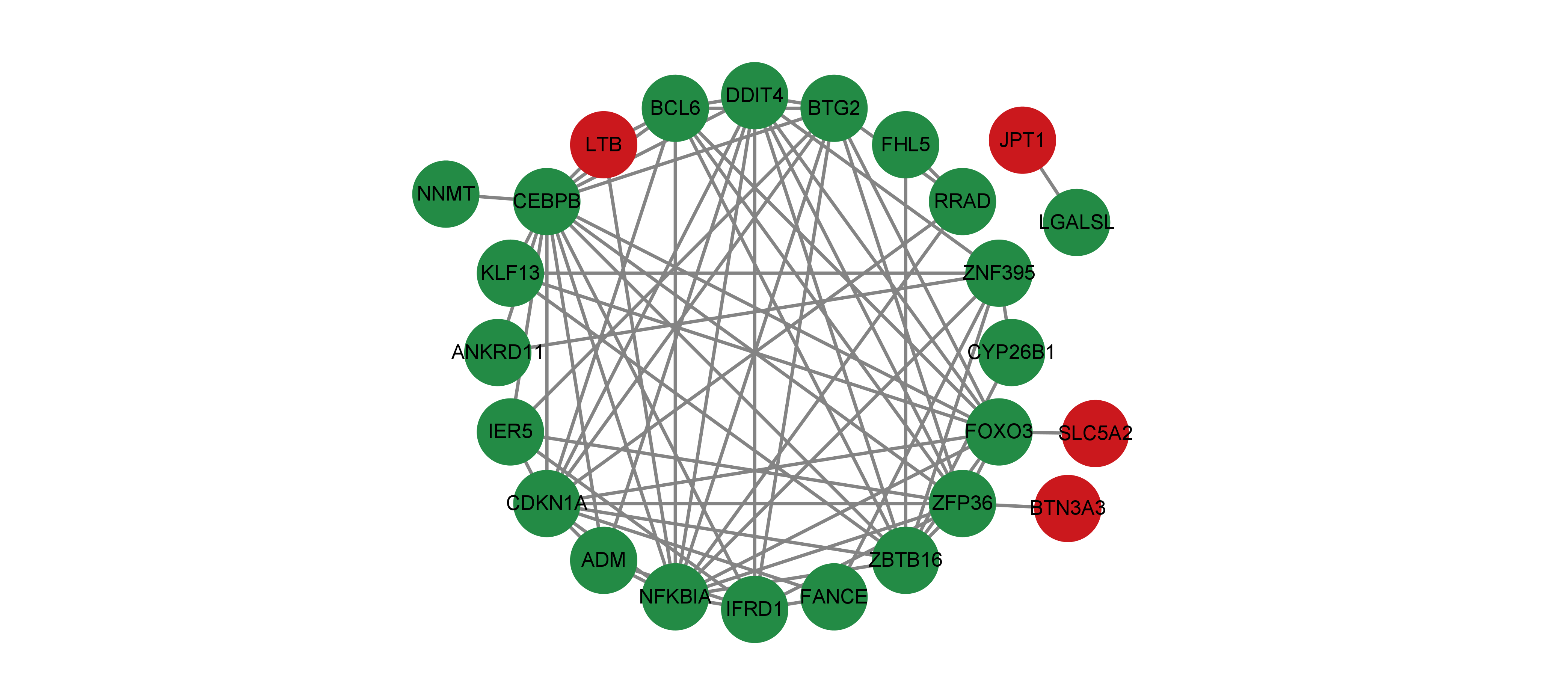

Supplement: Supplementary file 4 [file Data_Sheet_2.ZIP › raw data(2)/11.cytoscape/gengxing.png]

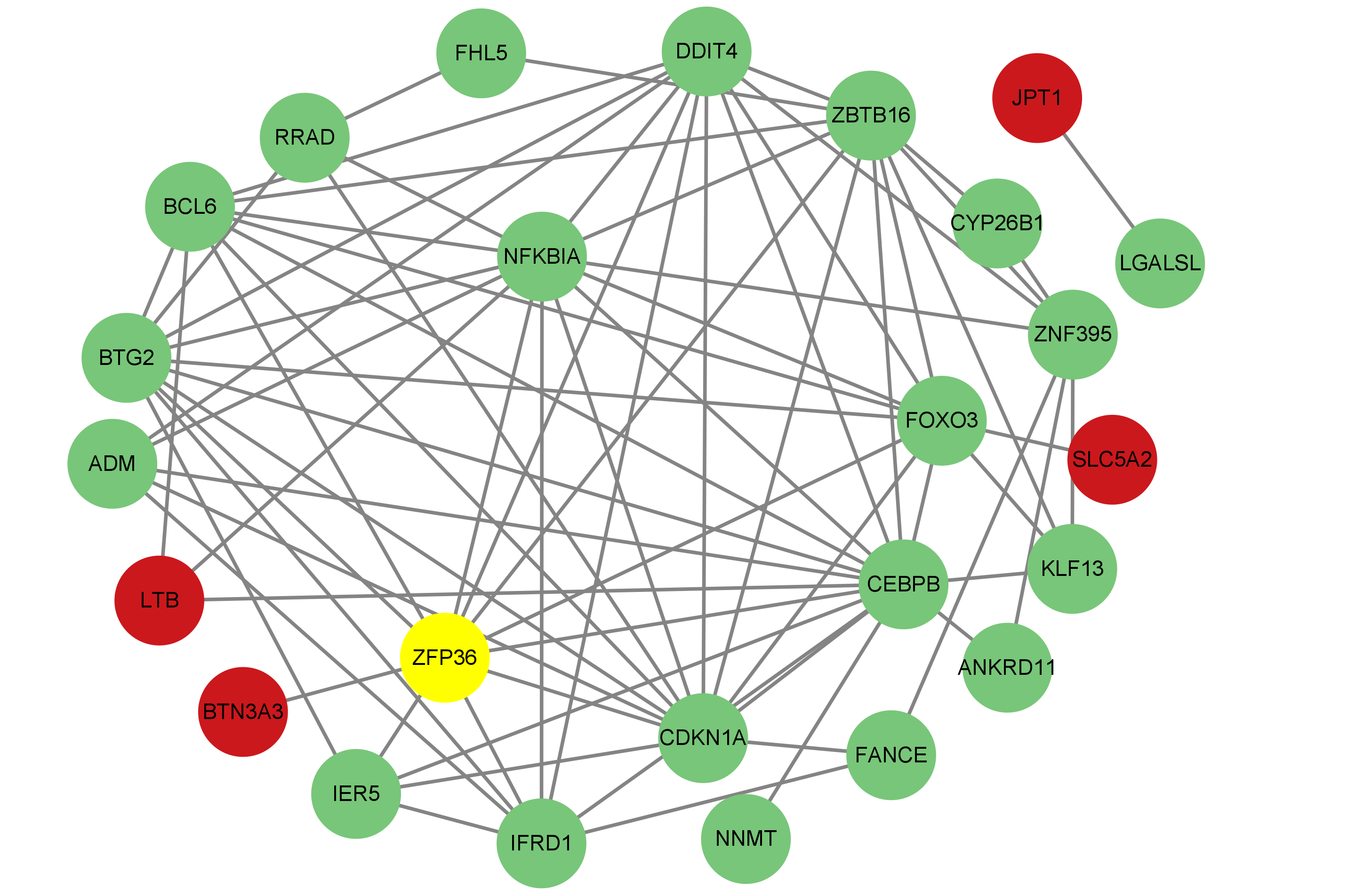

Supplement: Supplementary file 4 [file Data_Sheet_2.ZIP › raw data(2)/11.cytoscape/net.network.txt.png]

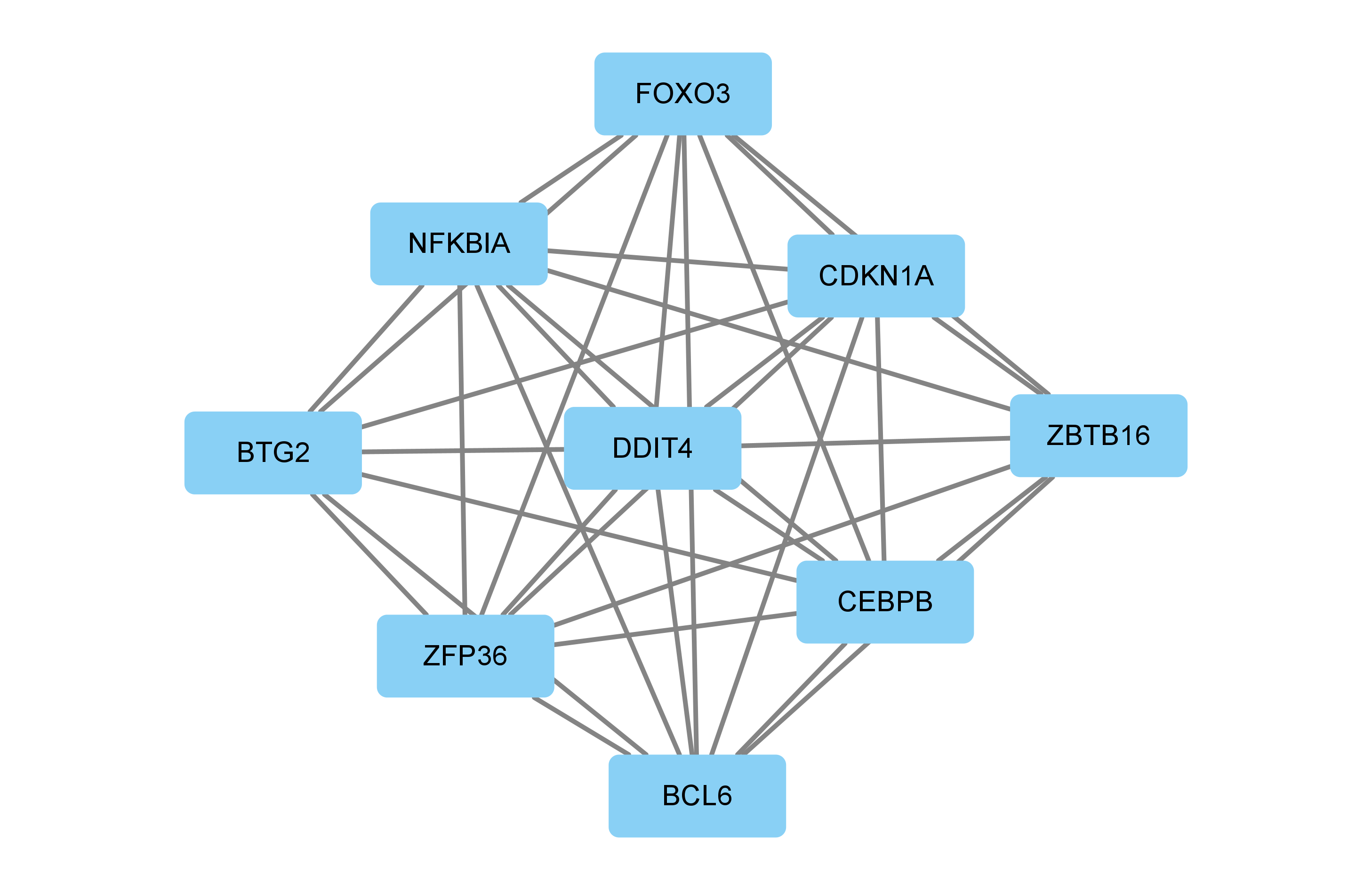

Supplement: Supplementary file 4 [file Data_Sheet_2.ZIP › raw data(2)/12.MCODE/network 1.png]

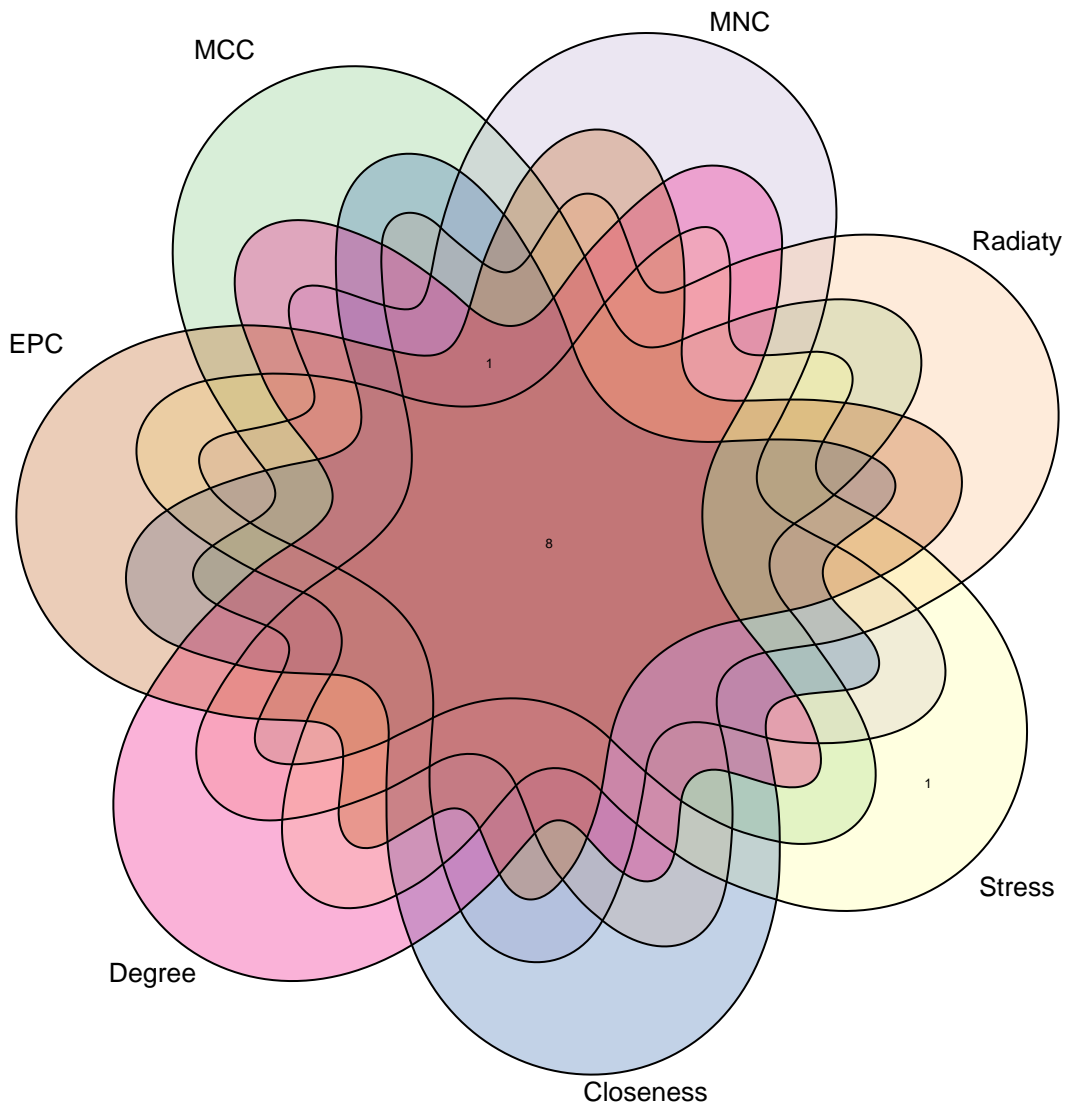

Supplement: Supplementary file 4 [file Data_Sheet_2.ZIP › raw data(2)/13.cytoHubba/7veen.pdf]

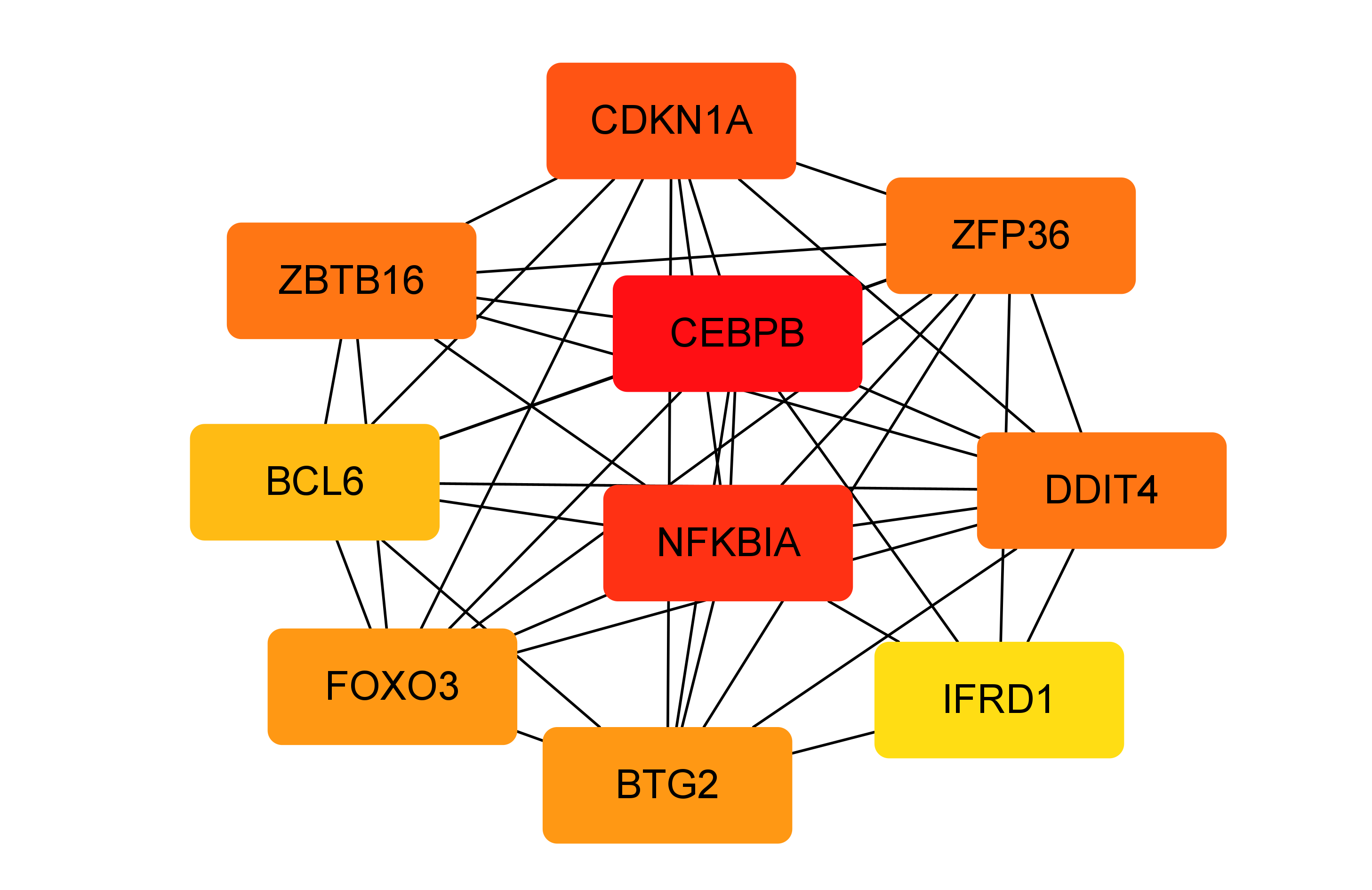

Supplement: Supplementary file 4 [file Data_Sheet_2.ZIP › raw data(2)/13.cytoHubba/hubGenes.png]

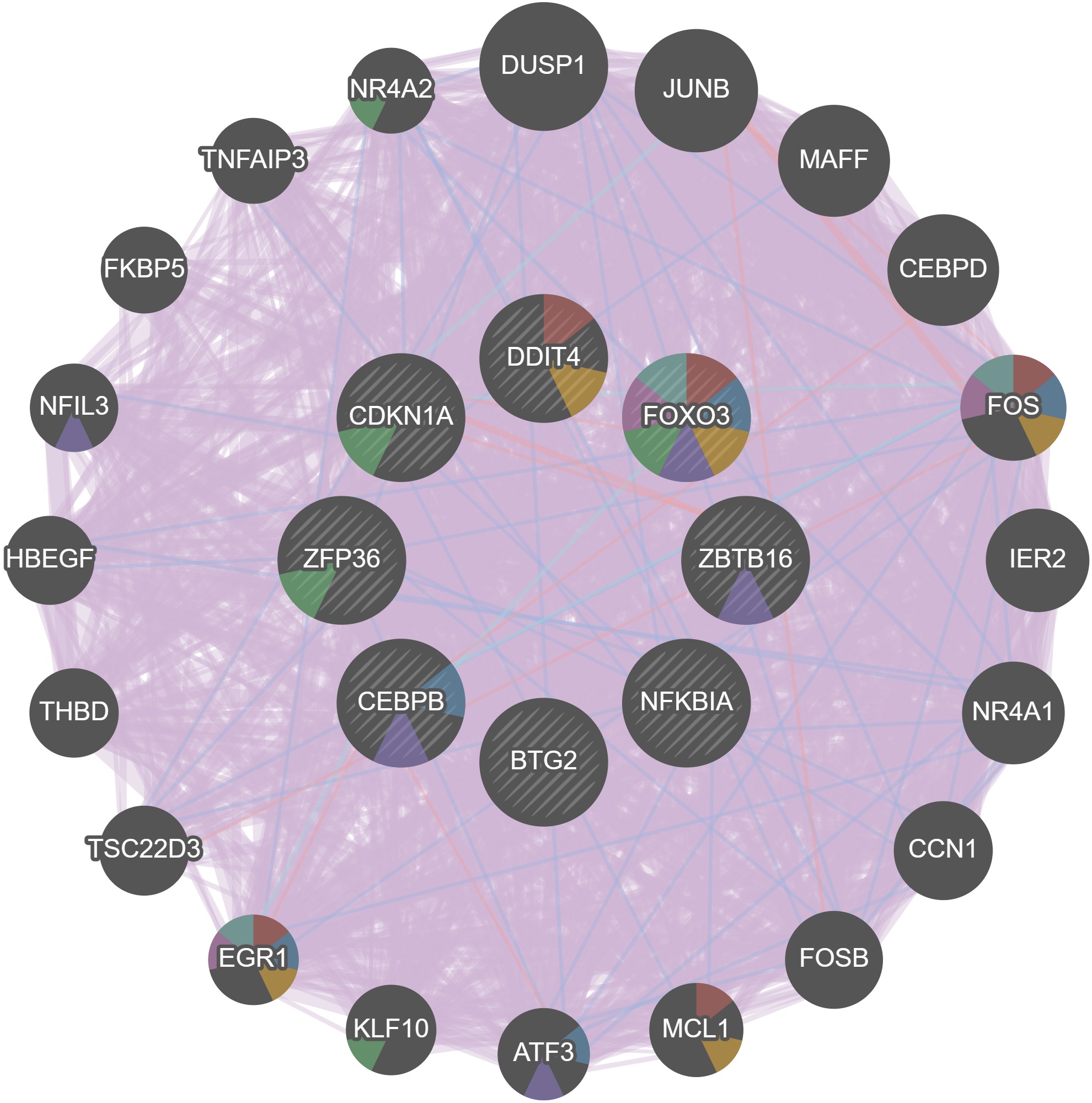

Supplement: Supplementary file 4 [file Data_Sheet_2.ZIP › raw data(2)/14.GeneMANIA/0be30f1b24730d8370a2092e45f4daa.jpg]

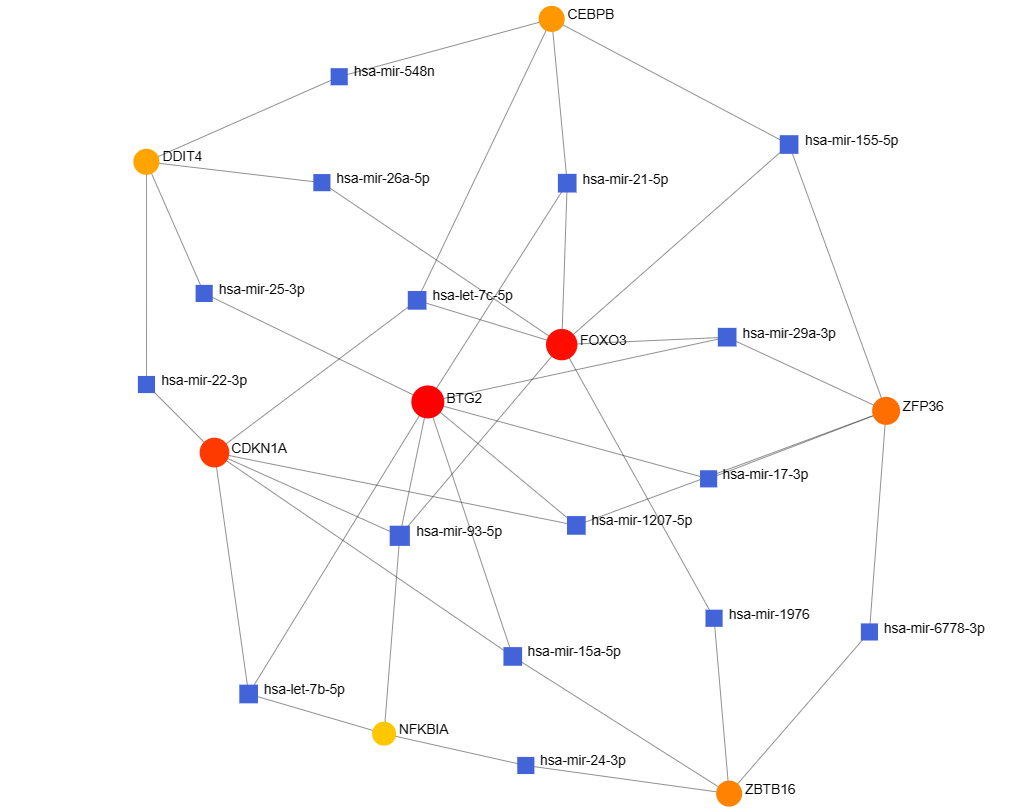

Supplement: Supplementary file 4 [file Data_Sheet_2.ZIP › raw data(2)/14.GeneMANIA/2023-10-3 at 00.45.21.png]

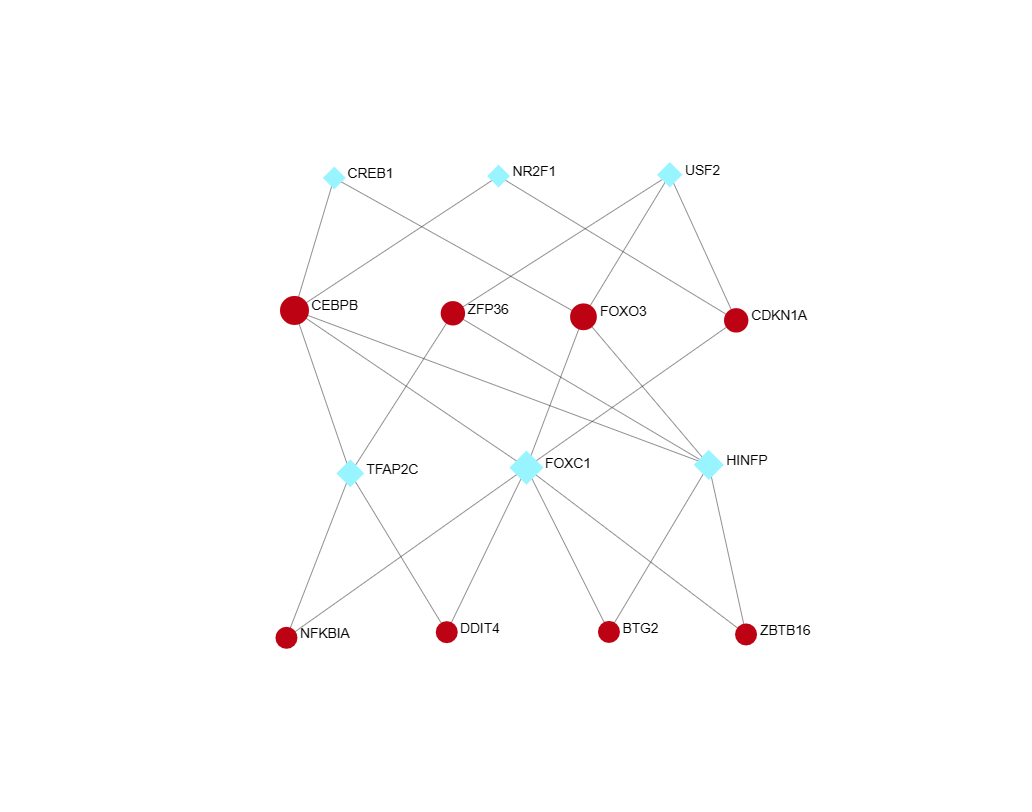

Supplement: Supplementary file 4 [file Data_Sheet_2.ZIP › raw data(2)/14.GeneMANIA/2023-10-3 at 00.49.19.png]

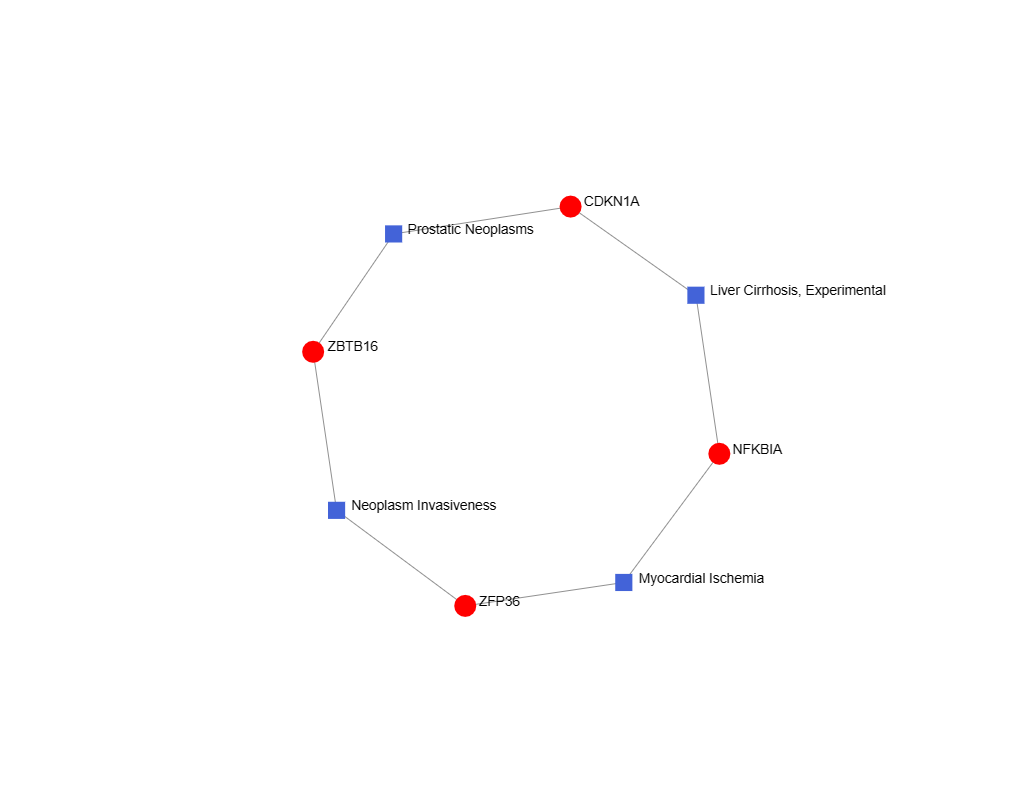

Supplement: Supplementary file 4 [file Data_Sheet_2.ZIP › raw data(2)/14.GeneMANIA/2023-10-3 at 01.09.21.png]

# ADH7

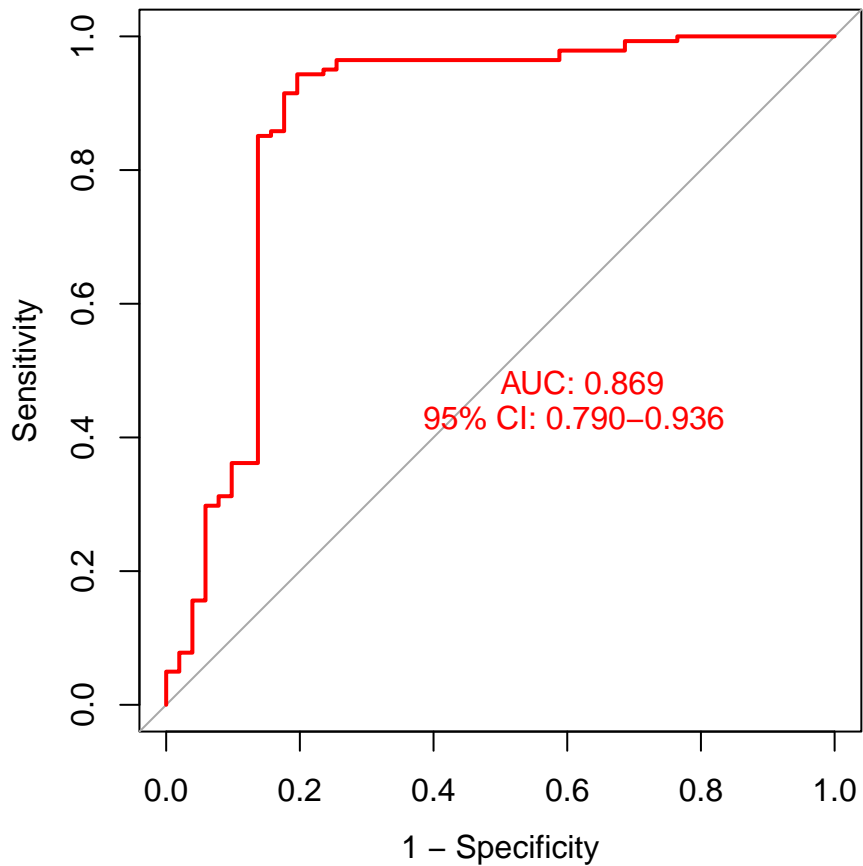

Supplement: Supplementary file 4 [file Data_Sheet_2.ZIP › raw data(2)/22.preTFCyto/16.ROC/ROC.ADH7.pdf]

# TMEM27

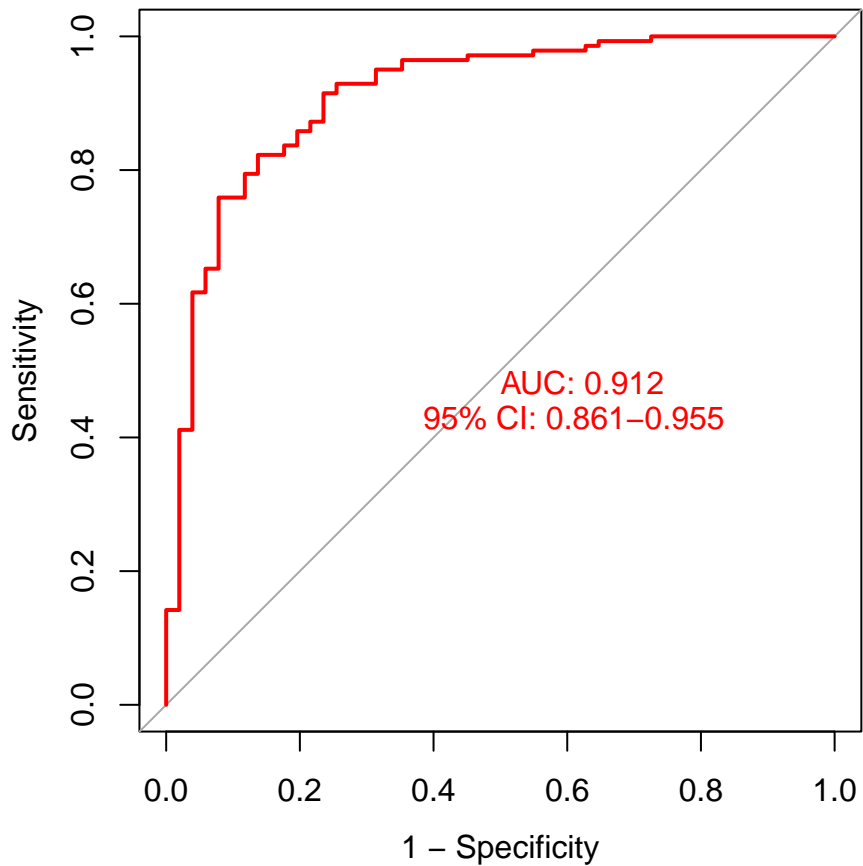

Supplement: Supplementary file 4 [file Data_Sheet_2.ZIP › raw data(2)/22.preTFCyto/16.ROC/ROC.TMEM27.pdf]

## BTG2

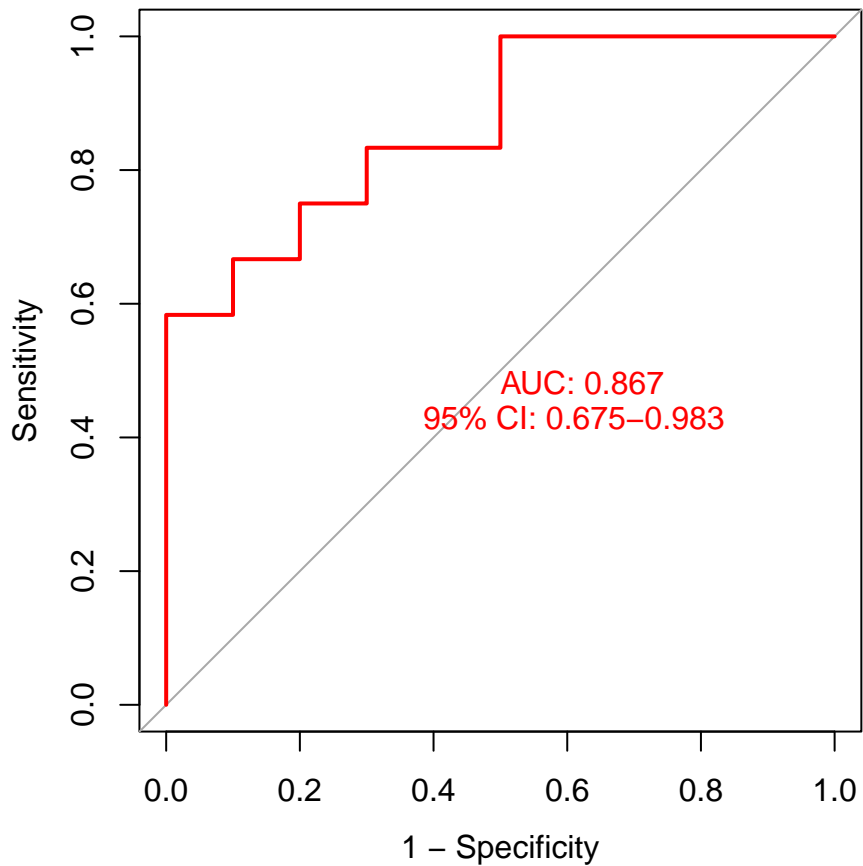

Supplement: Supplementary file 4 [file Data_Sheet_2.ZIP › raw data(2)/25.ROC/1428roc/ROC.BTG2.pdf]

# CDKN1A

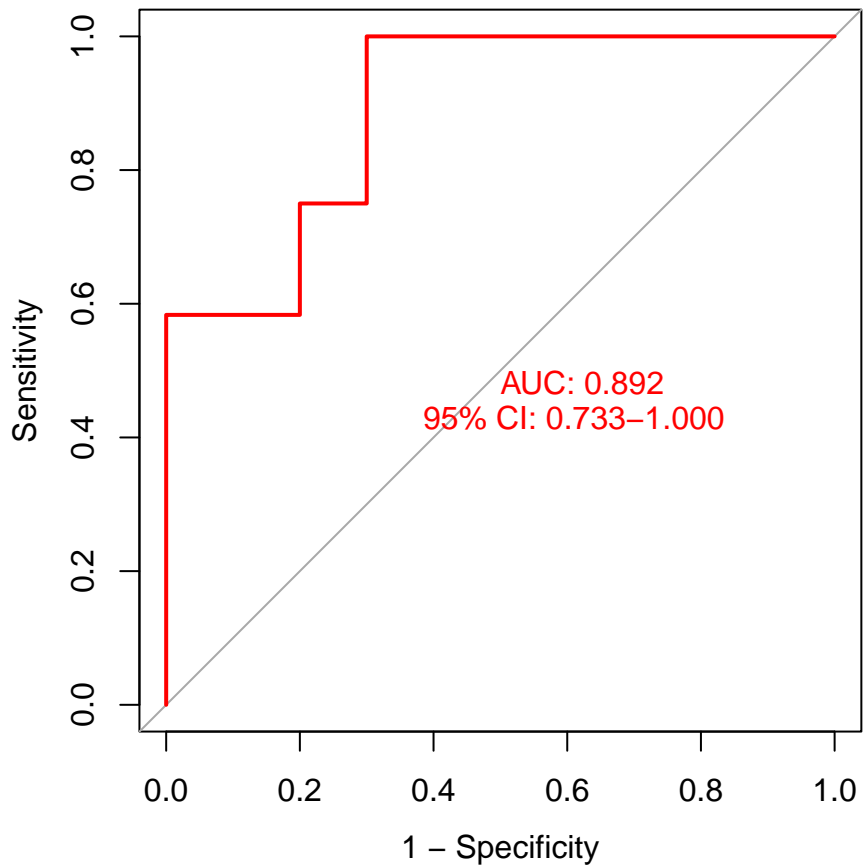

Supplement: Supplementary file 4 [file Data_Sheet_2.ZIP › raw data(2)/25.ROC/1428roc/ROC.CDKN1A.pdf]

# CEBPB

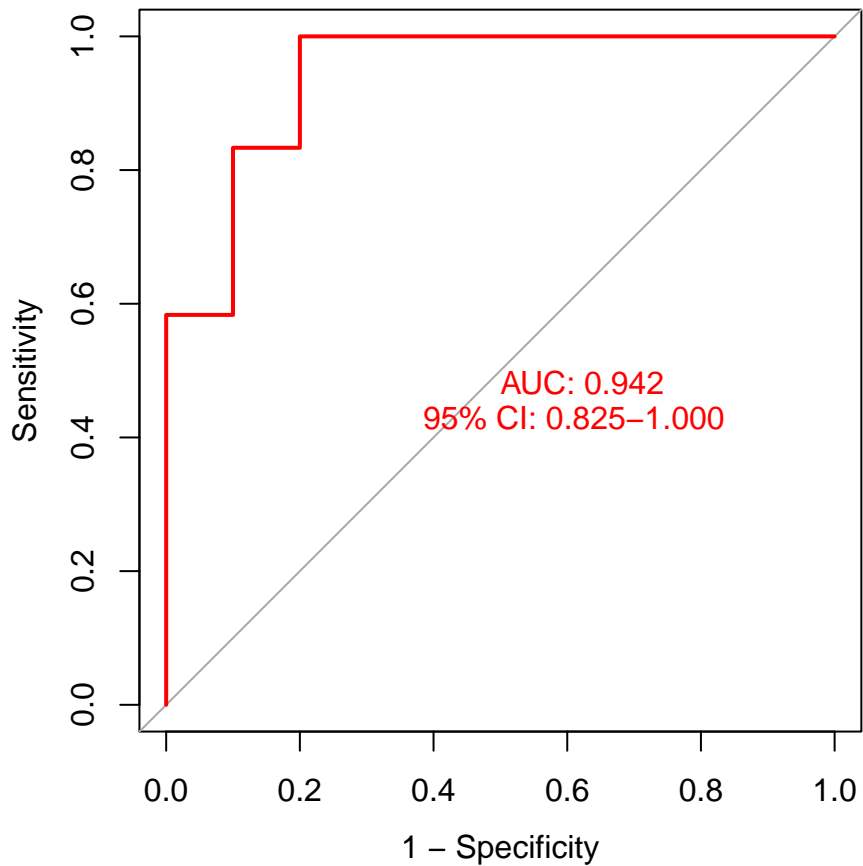

Supplement: Supplementary file 4 [file Data_Sheet_2.ZIP › raw data(2)/25.ROC/1428roc/ROC.CEBPB.pdf]

# DDIT4

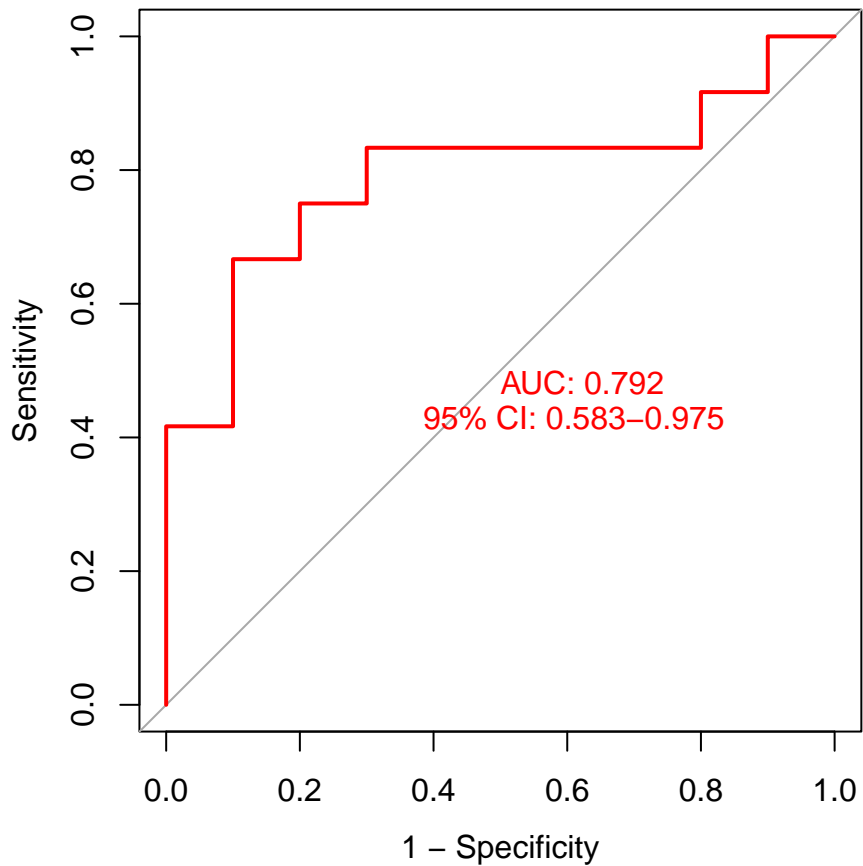

Supplement: Supplementary file 4 [file Data_Sheet_2.ZIP › raw data(2)/25.ROC/1428roc/ROC.DDIT4.pdf]

# FOXO3

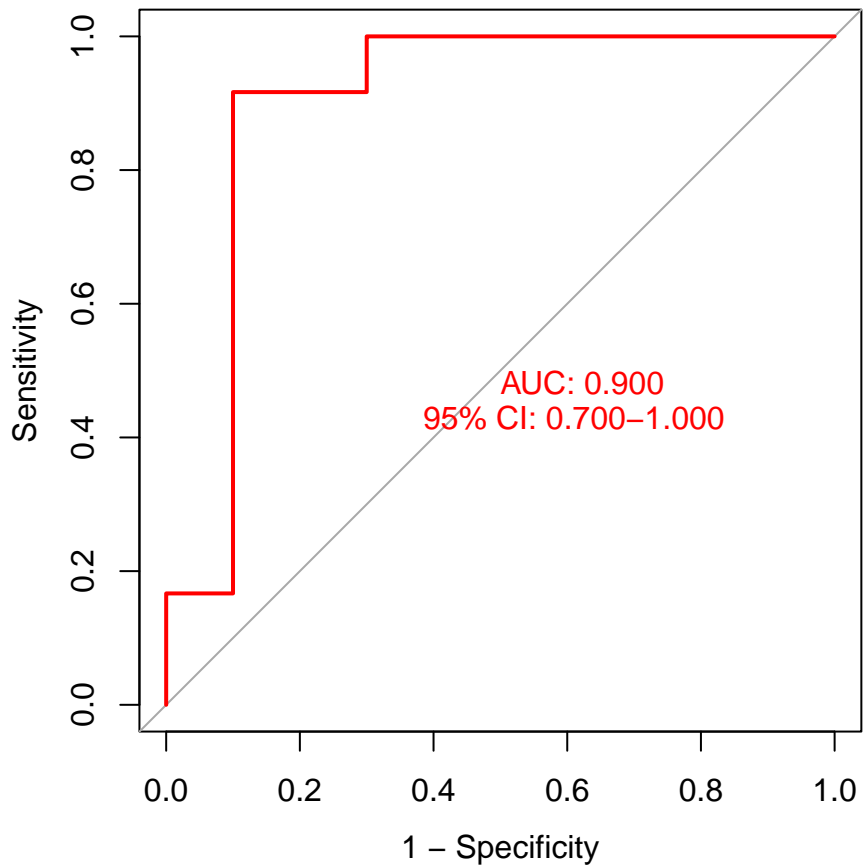

Supplement: Supplementary file 4 [file Data_Sheet_2.ZIP › raw data(2)/25.ROC/1428roc/ROC.FOXO3.pdf]

# NFKBIA

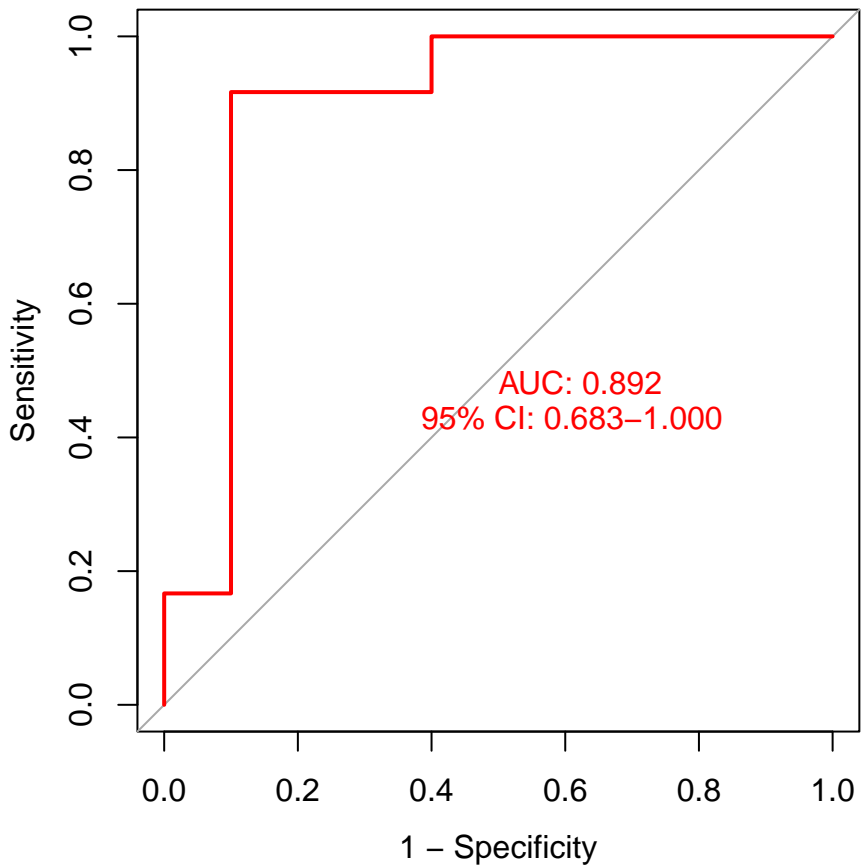

Supplement: Supplementary file 4 [file Data_Sheet_2.ZIP › raw data(2)/25.ROC/1428roc/ROC.NFKBIA.pdf]

# ZBTB16

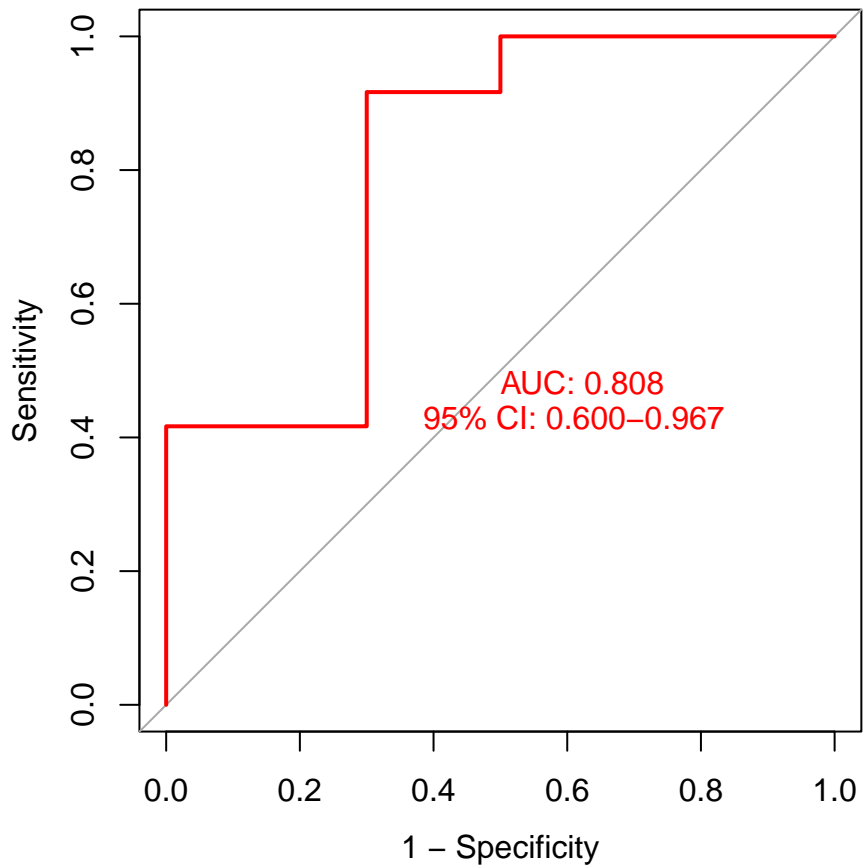

Supplement: Supplementary file 4 [file Data_Sheet_2.ZIP › raw data(2)/25.ROC/1428roc/ROC.ZBTB16.pdf]

# ZFP36

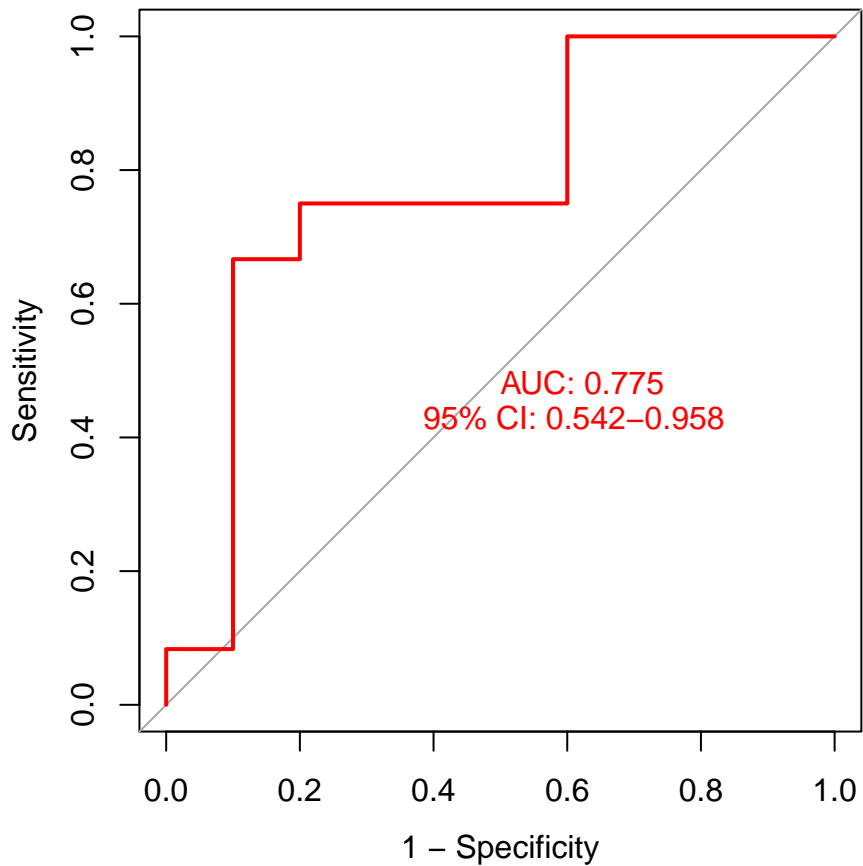

Supplement: Supplementary file 4 [file Data_Sheet_2.ZIP › raw data(2)/25.ROC/1428roc/ROC.ZFP36.pdf]

## BTG2

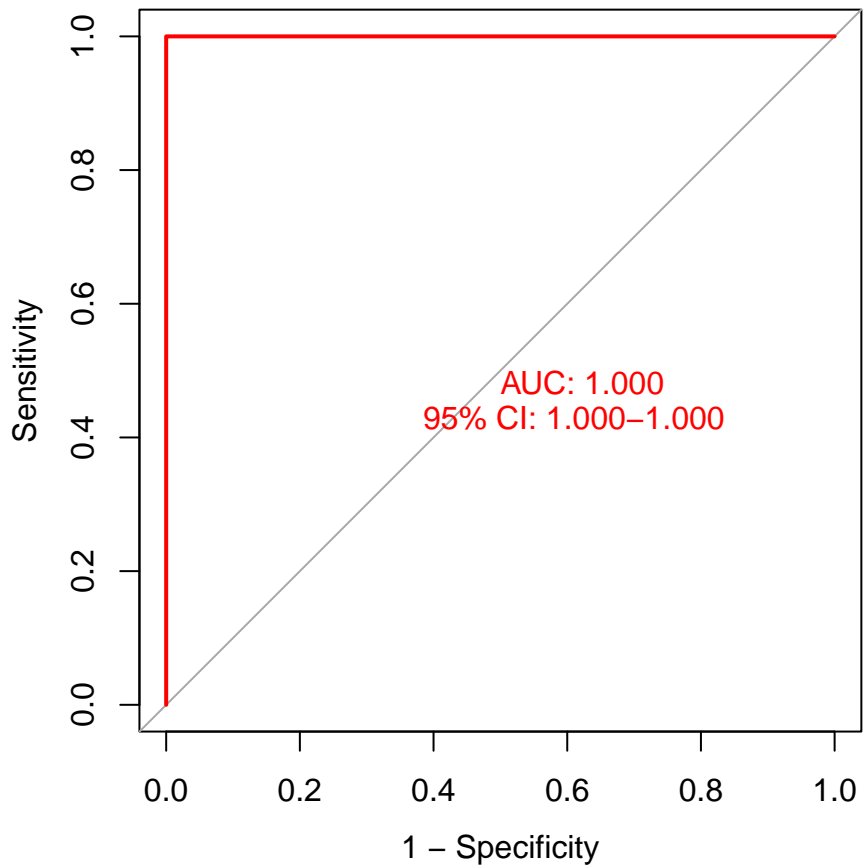

Supplement: Supplementary file 4 [file Data_Sheet_2.ZIP › raw data(2)/25.ROC/55235roc/ROC.BTG2.pdf]

# CDKN1A

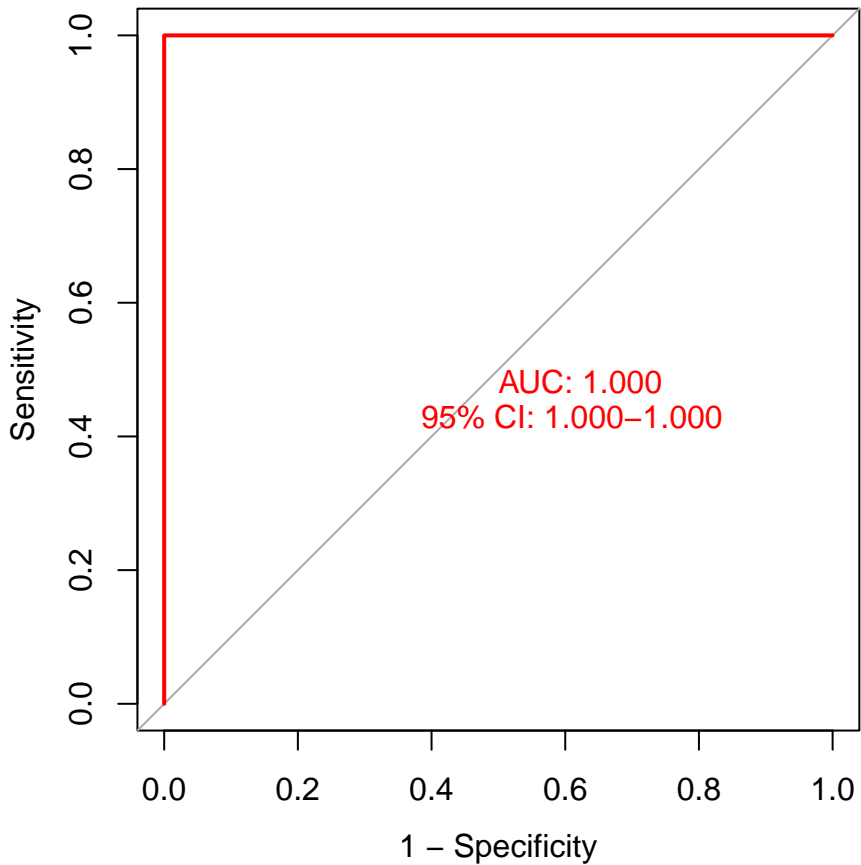

Supplement: Supplementary file 4 [file Data_Sheet_2.ZIP › raw data(2)/25.ROC/55235roc/ROC.CDKN1A.pdf]

# CEBPB

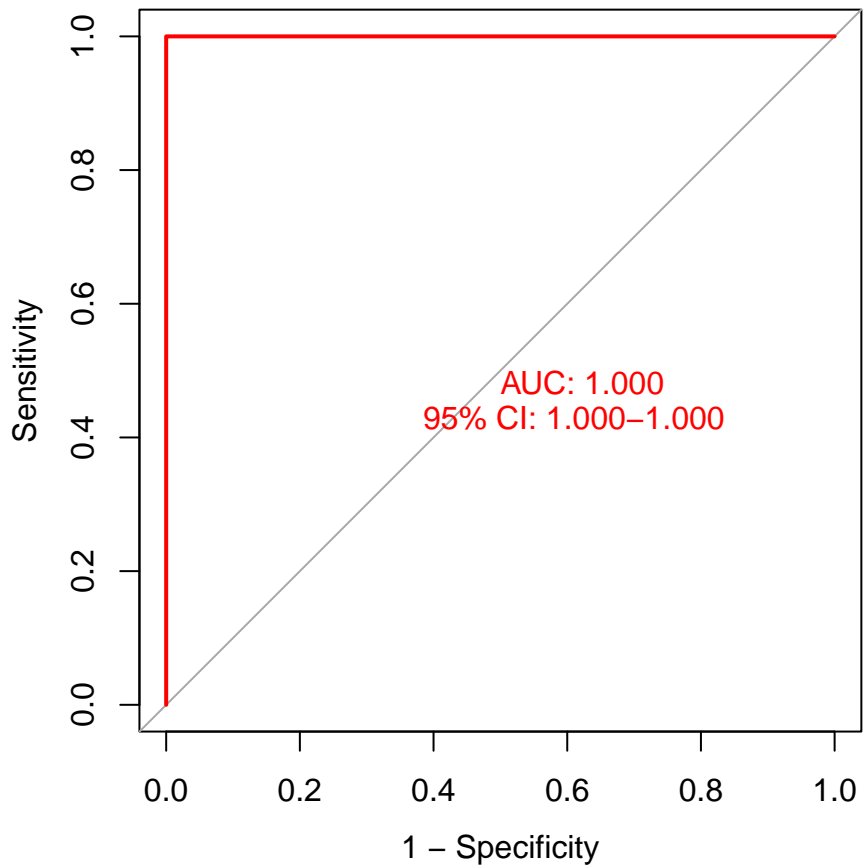

Supplement: Supplementary file 4 [file Data_Sheet_2.ZIP › raw data(2)/25.ROC/55235roc/ROC.CEBPB.pdf]

# DDIT4

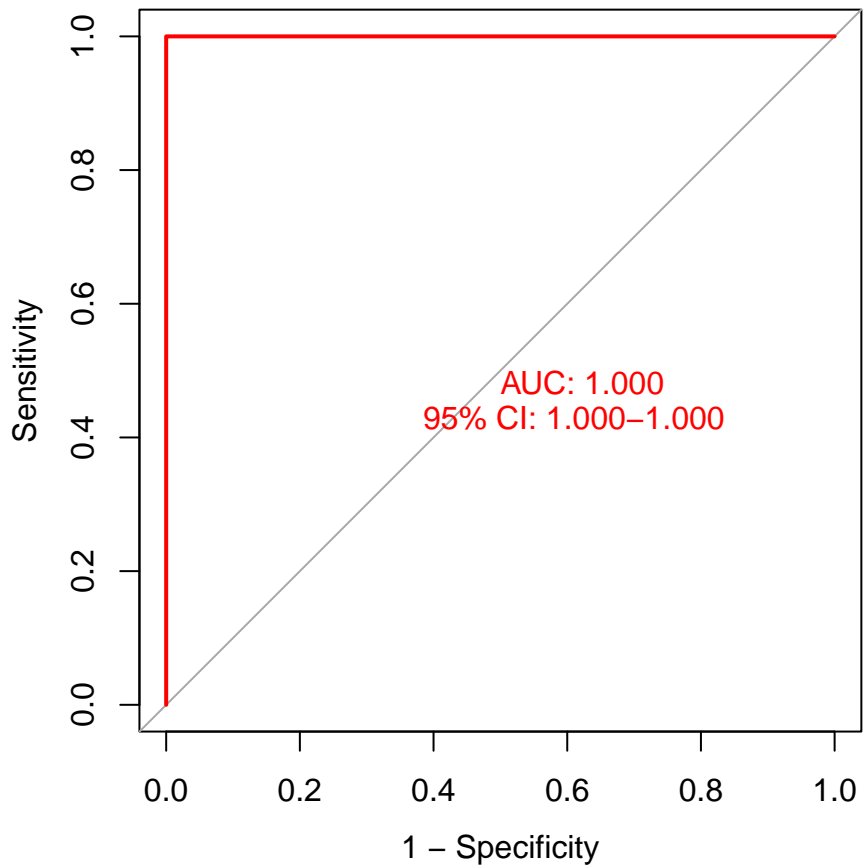

Supplement: Supplementary file 4 [file Data_Sheet_2.ZIP › raw data(2)/25.ROC/55235roc/ROC.DDIT4.pdf]

# FOXO3

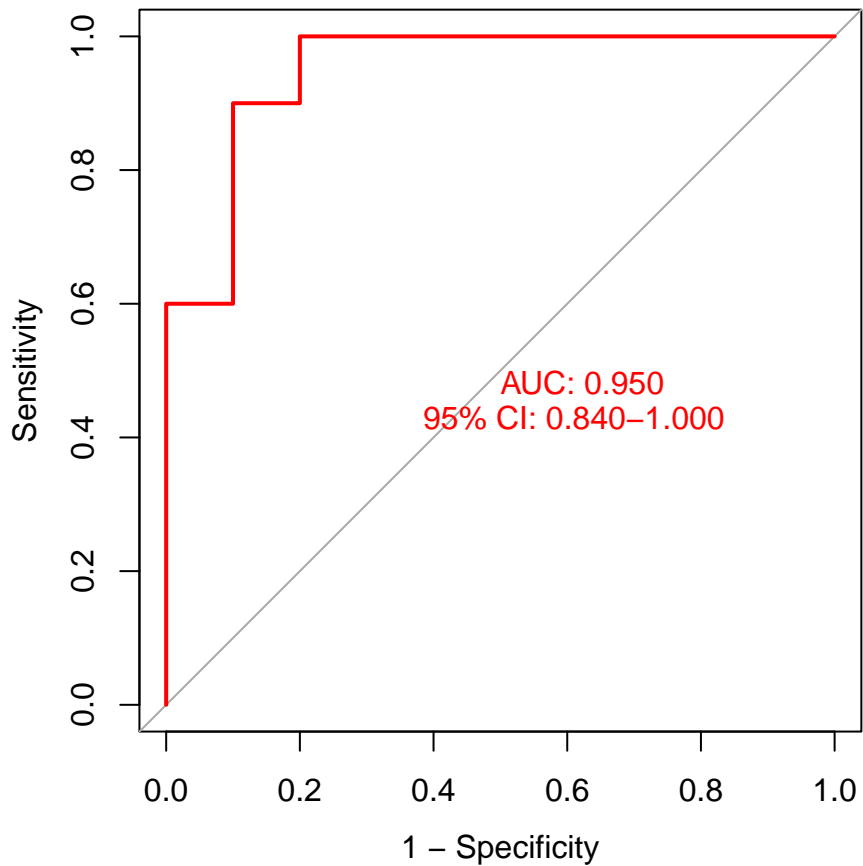

Supplement: Supplementary file 4 [file Data_Sheet_2.ZIP › raw data(2)/25.ROC/55235roc/ROC.FOXO3.pdf]

# NFKBIA

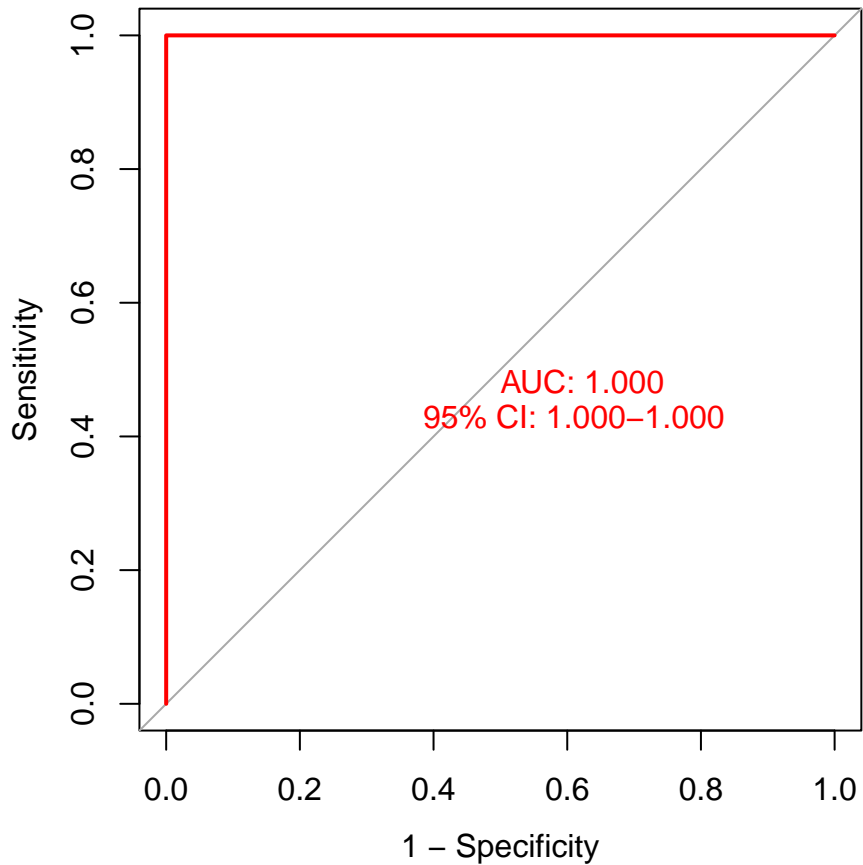

Supplement: Supplementary file 4 [file Data_Sheet_2.ZIP › raw data(2)/25.ROC/55235roc/ROC.NFKBIA.pdf]

# ZBTB16

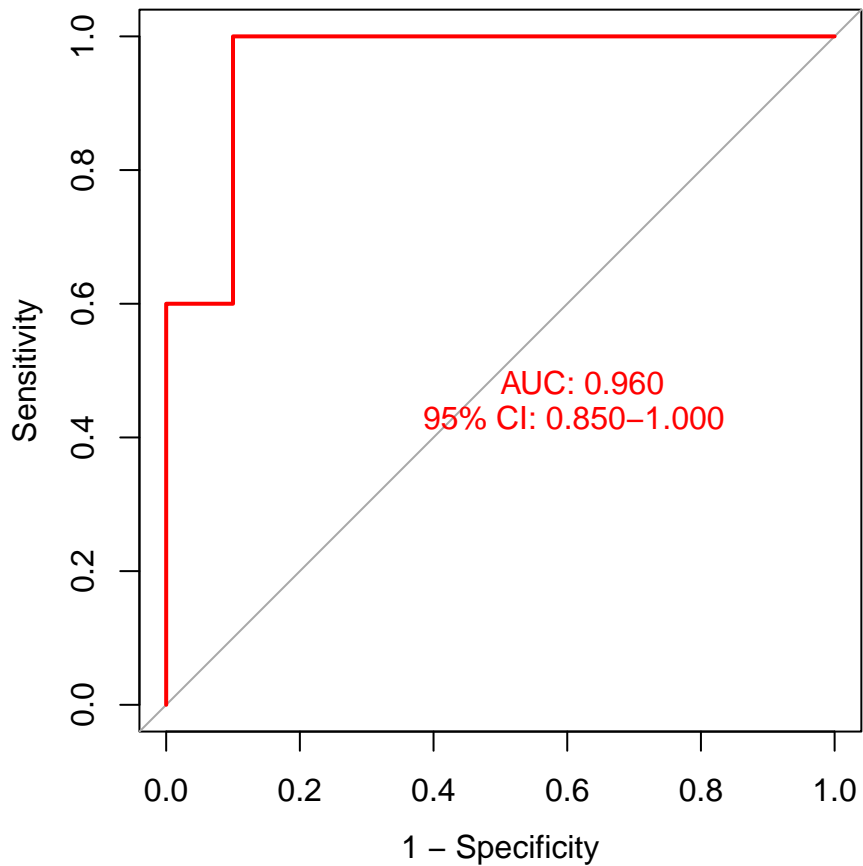

Supplement: Supplementary file 4 [file Data_Sheet_2.ZIP › raw data(2)/25.ROC/55235roc/ROC.ZBTB16.pdf]

# ZFP36

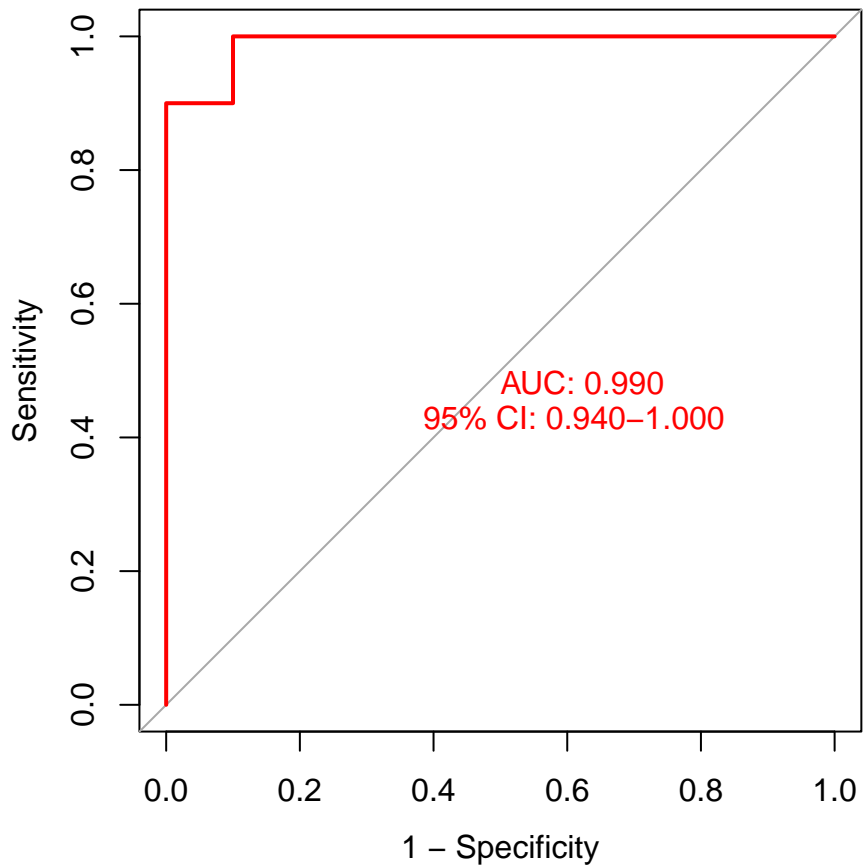

Supplement: Supplementary file 4 [file Data_Sheet_2.ZIP › raw data(2)/25.ROC/55235roc/ROC.ZFP36.pdf]

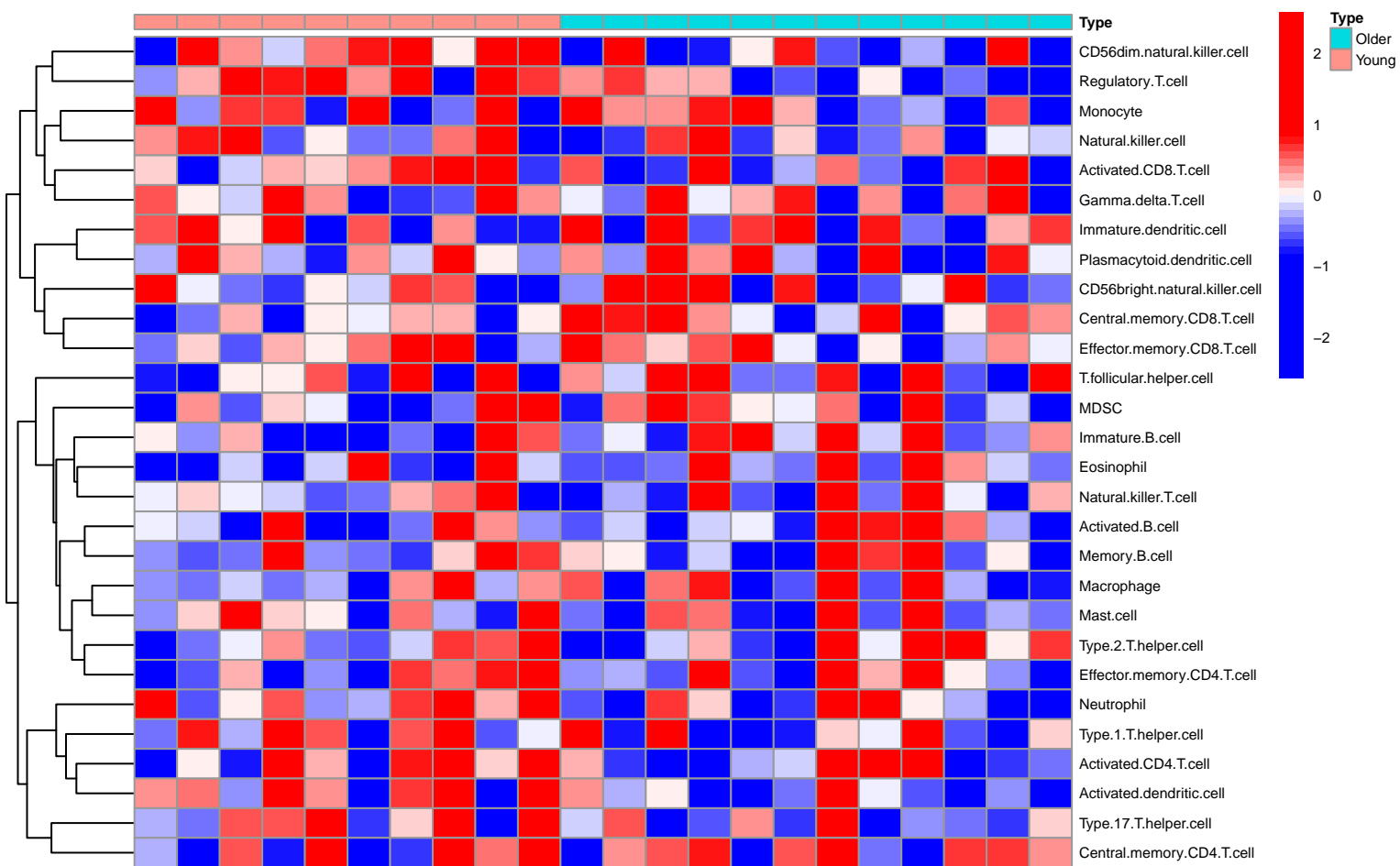

Supplement: Supplementary file 4 [file Data_Sheet_2.ZIP › raw data(2)/27.heatmap/1428/heatmap.pdf]

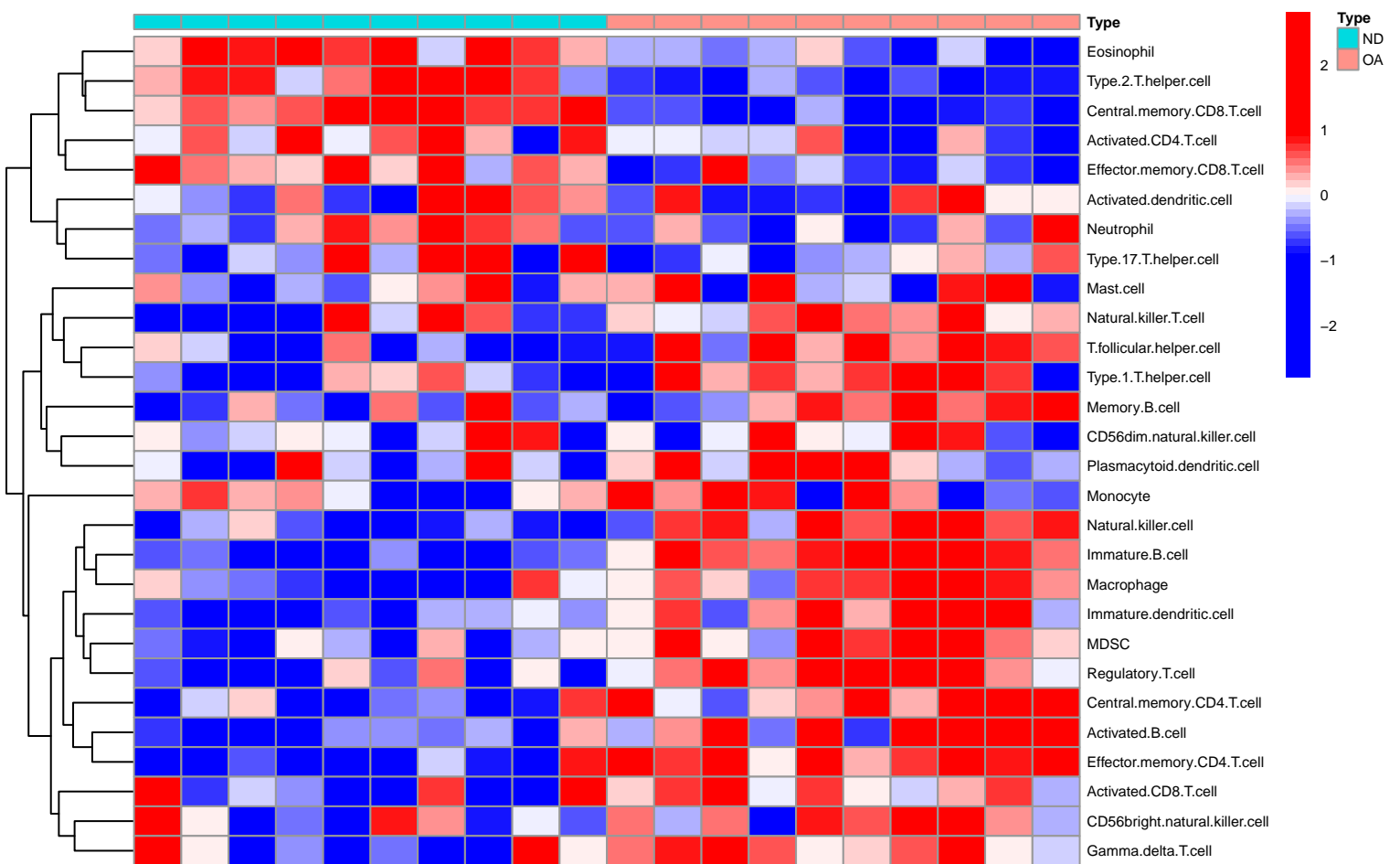

Supplement: Supplementary file 4 [file Data_Sheet_2.ZIP › raw data(2)/27.heatmap/55235/heatmap.pdf]

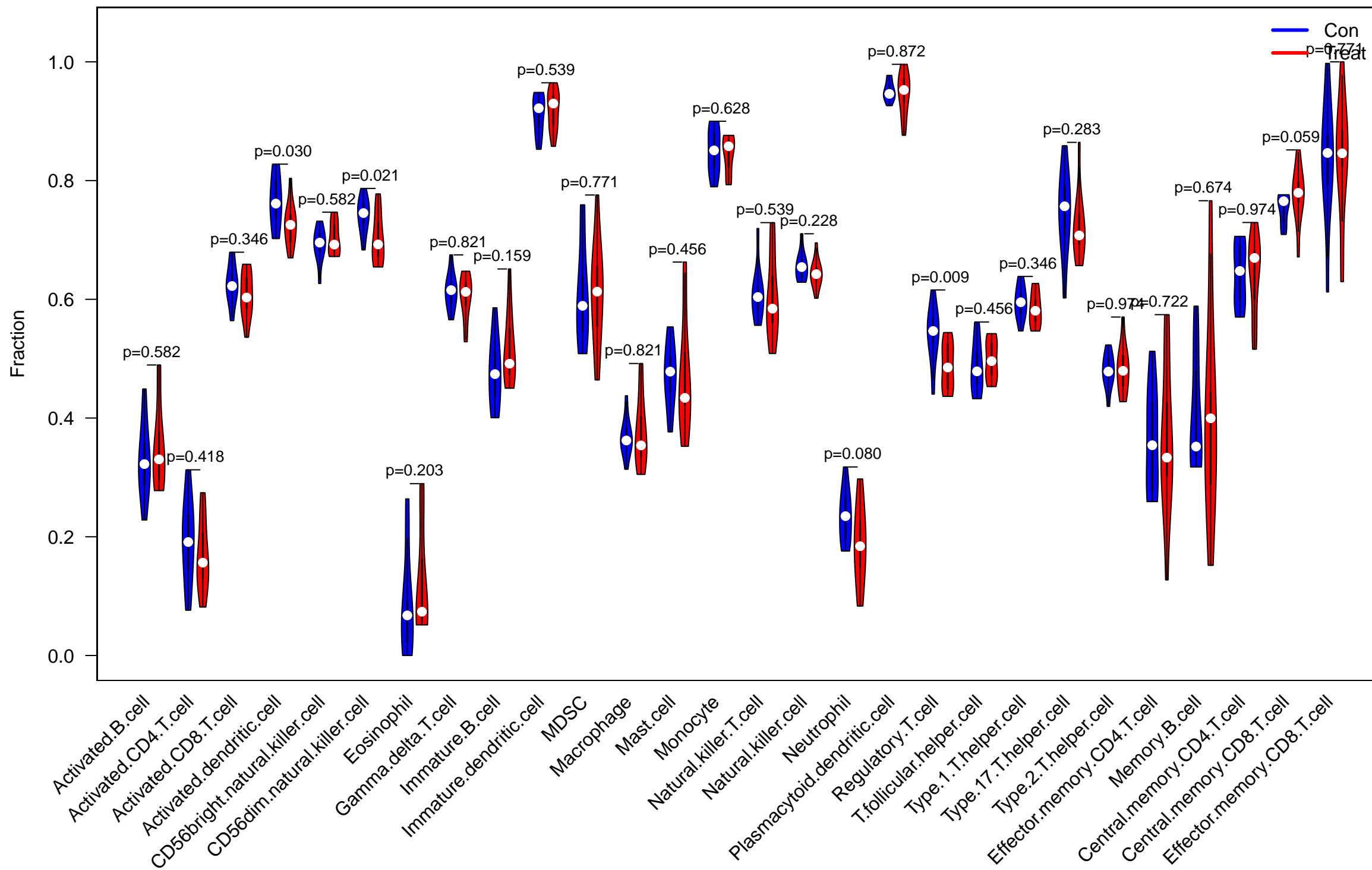

Supplement: Supplementary file 4 [file Data_Sheet_2.ZIP › raw data(2)/28.vioplot/1428/vioplot.pdf]

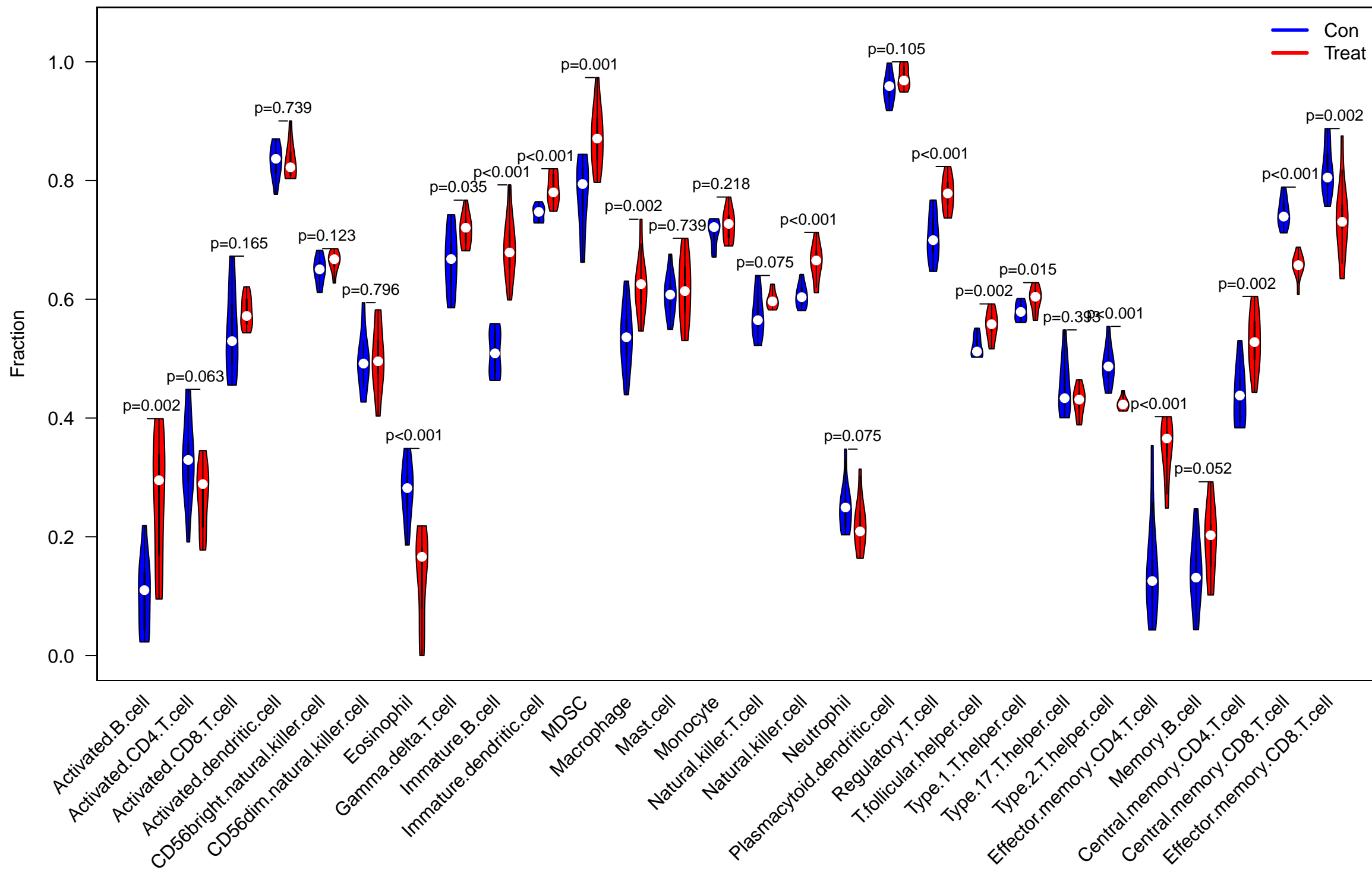

Supplement: Supplementary file 4 [file Data_Sheet_2.ZIP › raw data(2)/28.vioplot/55235/vioplot.pdf]

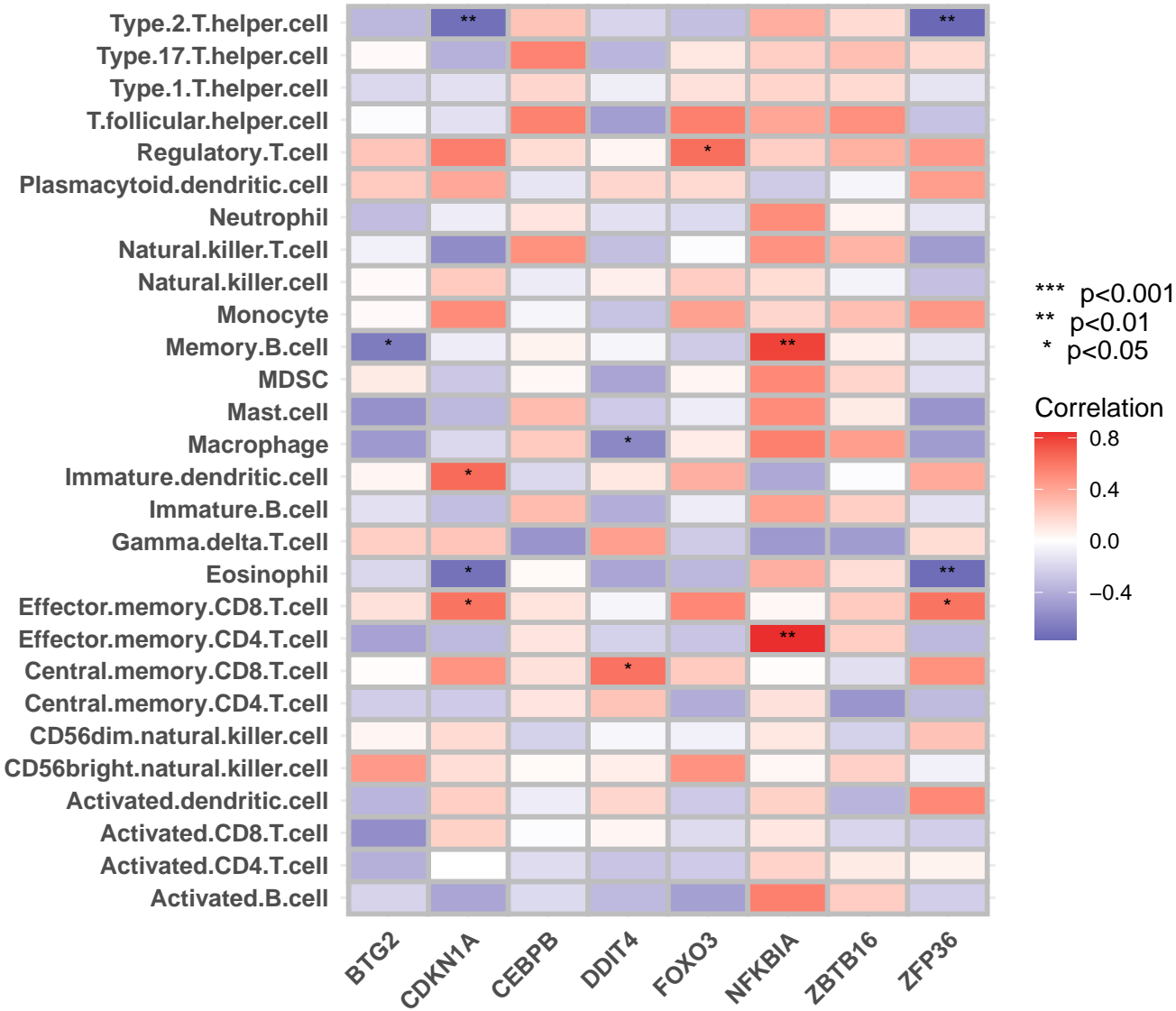

Supplement: Supplementary file 4 [file Data_Sheet_2.ZIP › raw data(2)/29.immuneCor/1428/cor.pdf]

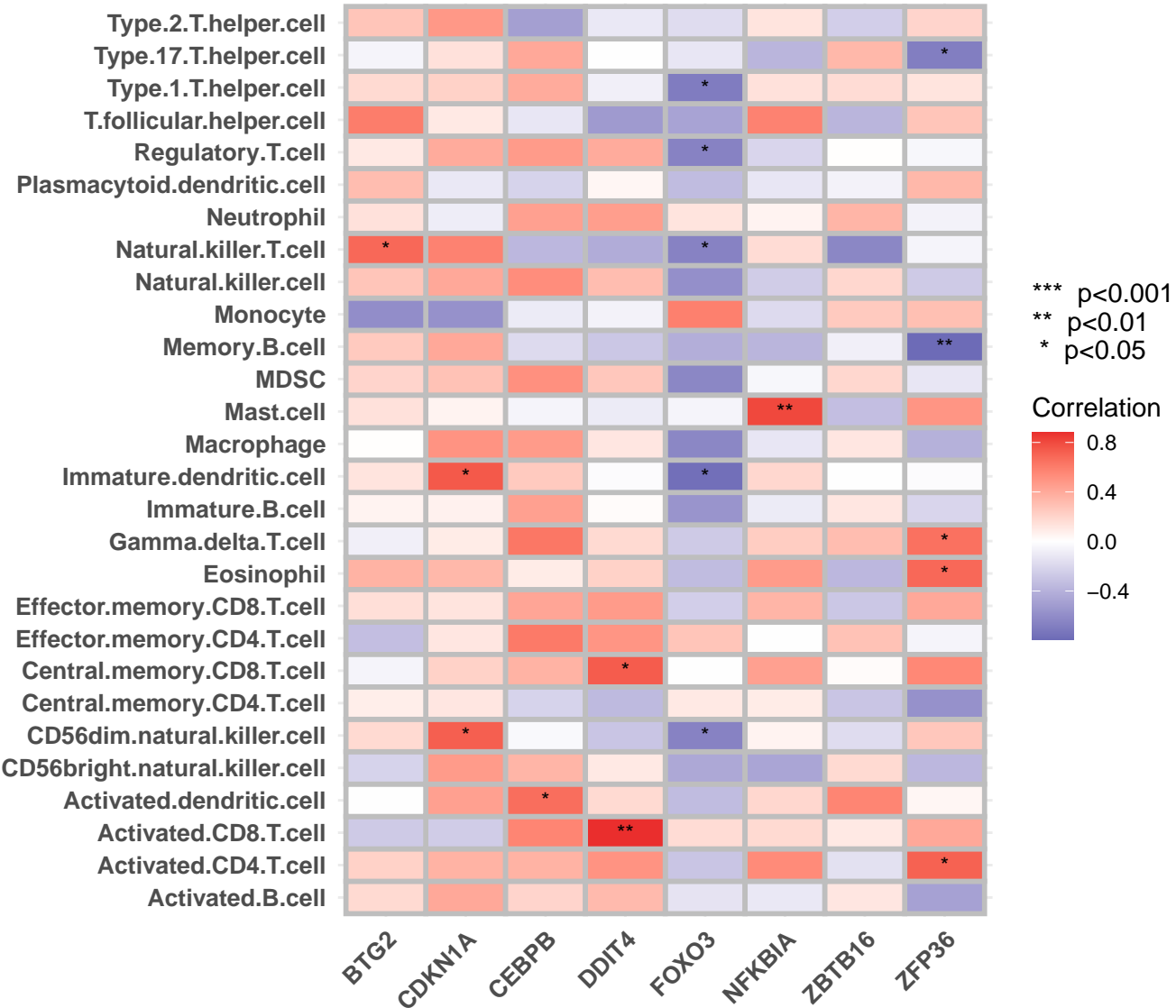

Supplement: Supplementary file 4 [file Data_Sheet_2.ZIP › raw data(2)/29.immuneCor/55235/cor.pdf]

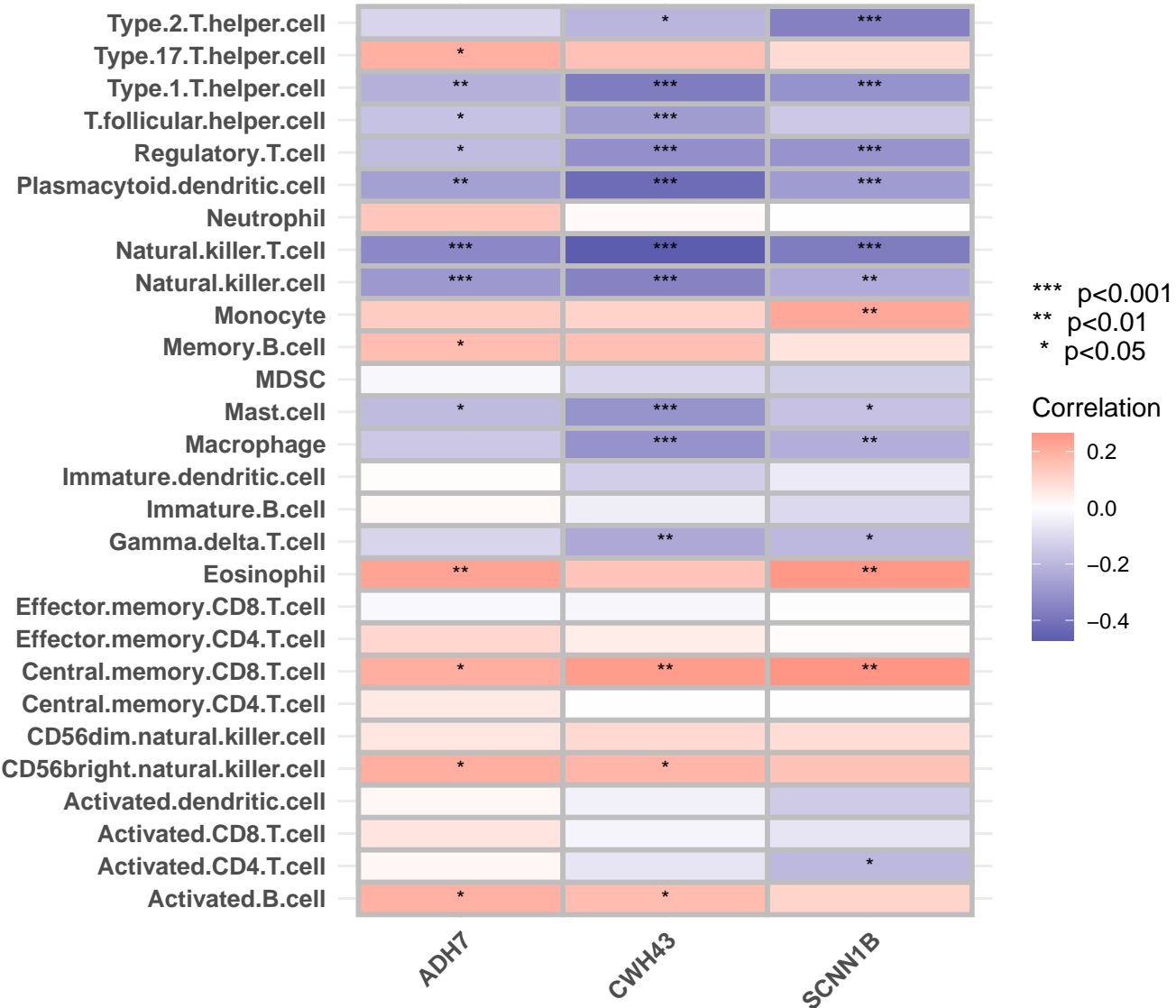

Supplement: Supplementary file 4 [file Data_Sheet_2.ZIP › raw data(2)/29.immuneCor/cor.pdf]

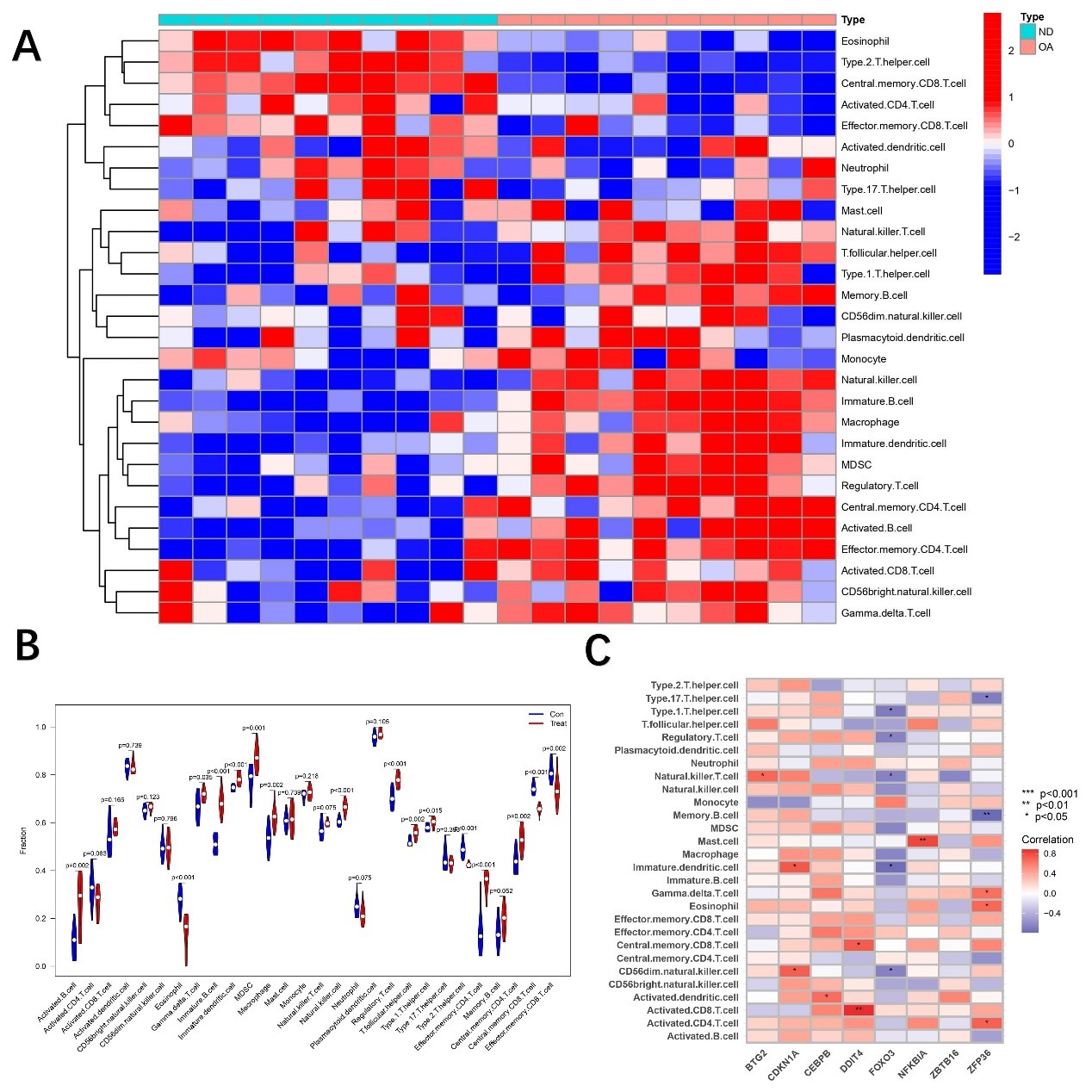

Supplement: Supplementary file 5 [file Image_1.JPEG]
